# Transcriptome-wide study of TNF-inhibitor therapy in rheumatoid arthritis reveals early signature of successful treatment

**DOI:** 10.1186/s13075-021-02451-9

**Published:** 2021-03-10

**Authors:** James OIiver, Nisha Nair, Gisela Orozco, Samantha Smith, Kimme L. Hyrich, Ann Morgan, John Isaacs, Anthony G. Wilson, BRAGGSS, Anne Barton, Darren Plant

**Affiliations:** 1 Versus Arthritis Centre for Genetics and Genomics, Centre for Musculoskeletal Research, Manchester Academic Health Sciences Centre, The University of Manchester, Manchester, UK; 2 NIHR Manchester BRC, Manchester University Foundation Trust, Manchester, UK; 3 Versus Arthritis Centre for Epidemiology, Centre for Musculoskeletal Research, Manchester Academic Health Sciences Centre, The University of Manchester, Manchester, UK; 4 Leeds Institute of Rheumatic and Musculoskeletal Medicine, University of Leeds and NIHR Leeds Musculoskeletal Biomedical Research Unit, Leeds Teaching Hospitals NHS Trust, Leeds, UK; 5 Institute of Cellular Medicine, Newcastle University, Newcastle upon Tyne, UK; 6 National Institute for Health Research Newcastle Biomedical Research Centre at Newcastle upon Tyne Hospitals NHS Foundation Trust and Newcastle University, Newcastle upon Tyne, UK; 7 UCD School of Medicine and Medical Science, Conway Institute, University College Dublin, Dublin, Ireland

**Keywords:** TNF-inhibitor, Adalimumab, Biomarkers, Gene expression, Longitudinal studies, Rheumatoid arthritis

## Abstract

**Background:**

Despite the success of TNF-inhibitor therapy in rheumatoid arthritis treatment, up to 40% of patients fail to respond adequately. This study aimed to identify transcriptome-based biomarkers of adalimumab response in rheumatoid arthritis (RA) to aid timely switching in non-responder patients and provide a better mechanistic understanding of the pathways involved in response/non-response.

**Methods:**

The Affymetrix Human Transcriptome Array 2.0 (HTA) was used to measure the transcriptome in whole blood at pre-treatment and at 3 months in EULAR good- and non-responders to adalimumab therapy. Differential expression of transcripts was analysed at the transcript level using multiple linear regression. Differentially expressed genes were validated in independent samples using OpenArray™ RT-qPCR.

**Results:**

In total, 813 transcripts were differentially expressed between pre-treatment and 3 months in adalimumab good-responders. No significant differential expression was observed between good- and non-responders at either time-point and no significant changes were observed in non-responders between time-points. OpenArray™ RT-qPCR was performed for 104 differentially expressed transcripts in good-responders, selected based on magnitude of effect or *p* value or based on prior association with RA or the immune system, validating differential expression for 17 transcripts.

**Conclusions:**

An early transcriptome signature of DAS28 response to adalimumab has been identified and replicated in independent datasets. Whilst treat-to-target approaches encourage early switching in non-responsive patients, registry evidence suggests that this does not always occur. The results herein could guide the development of a blood test to distinguish responders from non-responders at 3 months and support clinical decisions to switch non-responsive patients to an alternative therapy.

## Background

TNF-inhibitor (TNFi) therapies have revolutionised the treatment of rheumatoid arthritis (RA) for many patients, reducing synovial inflammation and long-term disability attributed to cartilage and bone destruction [1–3]. Despite their success, up to 40% of patients fail to respond adequately leaving them vulnerable to further disease progression and potential adverse effects of treatment [4, 5]. In addition, non-response is economically inefficient; the cost of TNFi therapy is estimated to be £3000–10,000 per patient per year. Thus, early identification of non-responders for switching to an alternative therapy is a research priority for improved long-term outcomes and the responsible use of limited healthcare resources.

Currently, non-responder patients can be identified and switched to an alternative therapy at 3 months, but many remain on an ineffective therapy for much longer periods [6]. Whilst clinical markers explain some of the variability in response, alone they offer insufficient predictive capability [7]. Ideally, a reliable biological biomarker or panel of biomarkers would be measured in newly diagnosed patients and throughout the treatment time-course to predict and monitor response to therapy. This would aid timely therapeutic switching in patients in whom the treatment is unlikely to be effective but requires the identification of reliable biomarkers and development of a statistical classifier of treatment response.

Prediction of TNFi response in RA has so far been disappointing with little evidence of replication between studies [8]. Progress has been hampered by small sample sizes, lack of replication, and a paucity of reliable biomarkers of TNFi response that are needed to drive advanced statistical approaches to develop robust classifiers [9–11].

The aim of this study was to discover and validate biomarkers that are associated with DAS28 response to TNFi by comparing changes in the transcriptome of peripheral blood from RA patients who were good- and non-responders to adalimumab therapy. Whilst previous studies have utilised microarrays to investigate TNF treatment response in RA [12], to our knowledge, this is the first to utilise the human transcriptome array (HTA), which facilitates study of both gene and exon-level data.

## Methods

### Patient selection

Seventy patients were selected from the Biologics in Rheumatoid Arthritis Genetics and Genomics Study Syndicate (BRAGGSS), previously described [7], from contributing UK centres. Inclusion criteria specified that participants provided informed written consent, were Caucasian, were over 18 years of age, and fulfilled the 2010 American College of Rheumatology (ACR)/European League Against Rheumatism (EULAR) criteria for RA [13].

All patients were biologic naïve, had received previous treatment with DMARDs, and were selected if they were treated with adalimumab and could be categorised as good or non-responders to treatment at 3 months. Stringent inclusion criteria were applied to select responder groups. Good-responders were defined by a 28-joint count disease activity score (DAS28) of < 2.6 at follow-up (i.e. clinical remission) and an improvement of > 1.2. Non-responders were included if their improvement in DAS28 was < 0.6 with an endpoint DAS28 of > 5.1 (i.e. high disease activity). Non-responder patients were excluded if anti-drug antibodies, measured by radioimmunoassay at 3-month follow-up, were detected in serum samples and/or if they self-reported non-adherence [14, 15].

### Blood collection

Pre-treatment and 3-month post-treatment blood samples were collected into Tempus™ Blood RNA Tubes (3 ml) (Applied Biosystems, Foster City, CA, USA). Once collected, samples were shipped to the Versus Arthritis Centre for Genetics and Genomics laboratory for central processing. Samples were logged onto the laboratory information management system (LIMS) and were stored at − 80 °C until RNA isolation.

### RNA isolation

Total RNA was isolated from Tempus™ whole blood using the MagMAX™ for Stabilised Blood Tubes RNA Isolation Kit, compatible with Tempus™ Blood RNA Tubes (Life Technologies, Carlsbad, CA, USA), according to manufacturer’s instructions. Extraction batches were mixed for responders and non-responders in order to reduce technical bias. Extracted RNA samples were treated with DNase to eliminate any potential genomic DNA contamination from downstream transcriptome measurement. RNA was quantified using the Nanodrop ND-1000 (Thermo Scientific, Waltham, MA, USA) and quality-assessed using the 2100 Bioanalyzer to generate an RNA integrity number (RIN; Agilent, Santa Clara, CA, USA).

### Transcriptome measurement

Total RNA (100 ng) was amplified and converted into biotinylated sense-strand cDNA targets using the Affymetrix WT PLUS kit according to the manufacturer’s instructions (Affymetrix, Santa Clara, CA, USA). All samples were collected using the same study protocol, irrespective of clinical features. We adhered to the manufacturer’s recommendations regarding RNA quality control (260/280 ratio between 1.7 and 2.1). The Nanodrop ND-1000 was used to monitor and normalise cDNA concentration across samples throughout the target preparation. Samples of different response status and time-point were arranged in the 96-well plate at random to avoid cDNA conversion bias. For each sample, 5-μg of fragmented, end-labelled sense-strand target cDNA was hybridised to a GeneChip Human Transcriptome Array (HTA) 2.0 before incubation for 16 h at 45 °C in the GeneChip® Hybridization Oven 645. Following hybridisation, arrays were washed and stained using the GeneChip® Fluidics Station 450 and scanned using the GeneChip® Scanner 3000 7G with Autoloader to generate a raw CEL data file for each sample.

### Statistical analysis of transcriptome

Raw CEL files were quality control assessed using the Affymetrix Expression Console software (version 1.1). All array files were then processed in the programming language ‘R’ using Bioconductor packages: The *pd.hta.2.0* package was used for platform design information annotation and the *affy* package was used to summarise probe-level data into a single expression measure for each individual transcript and pre-process the data. The *affy* package was used to perform normalisation and probe specific background correction before summarising the probe set values into a single expression measure according to default settings. Highly variable probes were used to cluster samples and produce a dissimilarity matrix. The highly variable probes were used to create a cluster dendrogram of samples based on both transcript- and probe-level differences and illustrate how samples cluster to identify any major outliers of which there were none. *The PCAmethods* library was used to conduct principal component analysis (PCA) to test for run order effects and *limma* was used for differential expression analysis. The arrayWeights function in limma was used to assess array quality using default parameters. Differential expression analysis was adjusted for baseline DAS28, age, gender, concurrent DMARD use, and array weights. Pathway analysis was performed using the Ingenuity Pathway Analysis (IPA) tool (version 33559992) according to default settings.

### RT-qPCR validation

Results from the discovery analyses were ranked according to *p* value and also fold change. The top 10 hits according to most significant *p* value and the top 10 according to largest fold change were selected for validation. A further 84 transcripts that satisfied a fold change > 1.2 and a false discovery rate (FDR) < 0.05 were selected according to a known biological association with RA or the immune system. TaqMan™ assays were selected for a total of 104 transcripts from the initial adalimumab transcriptome study for custom design of a gene expression OpenArray™ Plate, 112 assay format. Housekeeping genes were used to normalise targets and calculate relative expression values: *ACTB* (Hs99999903_m1), *B2M* (Hs00187842_m1), *GAPDH* (Hs99999905_m1), and *HPRT1* (Hs02800695_m1). Responder groups were less stringently defined than in the discovery study. That is to say good-response to adalimumab were not necessarily in clinical remission at follow-up but did show large DAS28 improvements and were in low disease activity states (change in DAS28 > 2.78; mean = 3.08; *n* = 11) versus poor-responders (change in DAS28 < 1.04; mean = 0.83; *n* = 11). This enabled us to test whether the changes identified in the discovery cohort could be identified in patients with a less extreme response. Total RNA was reverse transcribed into single-stranded cDNA using the High Capacity cDNA Reverse Transcription Kit (Applied Biosystems) according to manufacturer’s instructions. Sample cDNA and TaqMan™ OpenArray™ Gene Expression Master Mix were loaded on OpenArray™ plates using the OpenArray™ AccuFill® software and OpenArray™ AccuFill® system (Applied Biosystems). RT-qPCR was performed on the QuantStudio™ 12 K Flex real-time qPCR system (Applied Biosystems) and analysed in the Relative Quantification Application on the Thermo Fisher Cloud (Thermo Scientific).

## Results

### Cohort characteristics

Seventy patients receiving adalimumab therapy were included in the initial study. Baseline characteristics for the patients are presented in Table 1.

**Table 1:** Baseline characteristics of RA patients included in the discovery study

Characteristic	Good-responders (*n* = 50)	Non-responders (*n* = 20)	*p* value
Age, mean (SD)	58.1 (13.1)	55.3 (13)	0.42^a^
Female, *n* (%)	31 (62)	15 (75)	0.30^b^
Days on drug at outcome, median (IQR)	113 (92, 147)	109 (93, 147)	0.74^c^
Baseline DAS28, mean (SD)	5.07 (0.90)	5.09 (0.90)	0.93^a^
Concurrent DMARD therapy, *n* (%)	46 (92)	15 (75)	0.06^b^
Swollen joint count, median (IQR)	8 (5, 11)	6 (3, 8)	0.13^c^
Tender joint count, median (IQR)	12 (6, 17)	17 (9, 23)	0.15^c^
Baseline HAQ score, median (IQR)	1.5 (1, 2.13)	1.8 (1.4, 2.3)	0.21^c^

Patients were stratified by EULAR response at 3 months. All patients receiving concurrent DMARD therapy were receiving methotrexate. *DAS28* 28-joint count disease activity score, *DMARD* disease modifying anti-rheumatic drugs, *HAQ* health assessment questionnaire, *SD* standard deviation, *IQR* interquartile range. *p* value: ^a^two-sample *t* test, ^b^chi-squared test, ^c^Wilcoxon rank-sum test

### Transcriptome measurement

PCA of the dataset revealed one principal component contributing significantly to sample variance (> 10%) which was adjusted for during downstream differential expression analysis. There was no correlation between this principal component and age, gender, DMARD use, baseline DAS28, DAS28 components, RIN, or RNA extraction batch. Differentially expressed transcripts between response groups and time-points were defined by a fold change > 1.2 and a false discovery rate (FDR) < 0.05. No significant differential expression was observed between good- and non-responders at either pre-treatment or 3 months and no significant changes were observed in non-responders between pre-treatment and 3 months. However, 813 transcripts were differentially expressed between time-points in good-responders, mapping to 491 unique genes. This comprised 202 transcripts that were more- and 611 transcripts that were less-abundant at 3 months, compared with the pre-treatment sample (Additional file 1: Table S1). Testing was performed to identify whether adjusting for RIN in the analysis was important, but this adjustment did not qualitatively change the results. RIN was not included in the final analysis because it was not available for all samples (*n* = 20).

Ingenuity pathway analysis was performed to identify enrichment of relevant pathways involving differentially expressed transcripts, which are either up- or downregulated (Table 2 or Additional file 2: Table S2). The most significantly enriched pathway within the differential expression dataset for adalimumab good-responders was in ‘B and T cell signalling in rheumatoid arthritis’ (*p* = 1.4E− 10) with TNF identified as one of the top upstream regulators (*p* = 1.53E− 06).

**Table 2:** Ingenuity pathway analysis (IPA)

Top canonical pathways		
Name	*p* value	Overlap
Altered T cell and B cell signalling in rheumatoid arthritis	1.41E− 10	17.3%, 14/81
TREM1 signalling	2.53E− 10	18.6%, 13/70
Dendritic cell maturation	5.68E− 08	9.5%, 16/169
Allograft rejection signalling	1.39E− 07	18.8%, 9/48
Communication between innate and adaptive immune cells	2.06E− 07	13.4%, 11/82
Top upstream regulators		
Upstream regulator	*p* value of overlap	
TGM2	4.66E− 15	
CEBPA	1.16E− 08	
CSF2	1.70E− 08	
TNF	1.53E− 06	
IRF4	2.33E− 06	
Top diseases and disorders		
Name	*p* value	Molecules
Immunological disease	6.30E− 03 to 1.20E− 22	151
Connective tissue disorders	5.32E− 03 to 3.31E− 21	81
Inflammatory disease	6.18E− 03 to 3.31E− 21	116
Skeletal and muscular disorders	5.32E− 03 to 3.31E− 21	106
Infectious diseases	5.76E− 03 to 5.84E− 20	76
Top molecular and cell functions		
Name	*p* value	Molecules
Cell-to-cell signalling and interaction	6.18E− 03 to 1.99E− 14	100
Cell death and survival	7.09E− 03 to 3.79E− 10	97
Cellular movement	6.44E− 03 to 4.26E− 10	55
Cellular development	6.48E− 03 to 5.54E− 10	49
Cellular growth and proliferation	6.18E− 03 to 5.54E− 10	84
Top physiological system development and function		
Name	*p* value	Molecules
Haematological system development and function	6.48E− 03 to 6.44E− 13	72
Immune cell trafficking	6.18E− 03 to 6.44E− 13	47
Embryonic development	1.86E− 05 to 1.86E− 05	4
Haematopoiesis	6.48E− 03 to 2.86E− 05	24
Hair and skin development and function	6.18E− 03 to 4.26E− 05	10

The differentially expressed genes in adalimumab good-responders (baseline versus 3 months) were analysed using Ingenuity Pathway Analysis (IPA) software. The top associated terms for the output themes, which were either up- or downregulated, are presented with associated *p* values. The table also displays the number of molecules, or the number of differentially expressed transcripts within the dataset associated with each term

### RT-qPCR validation

**Fig. 1:**
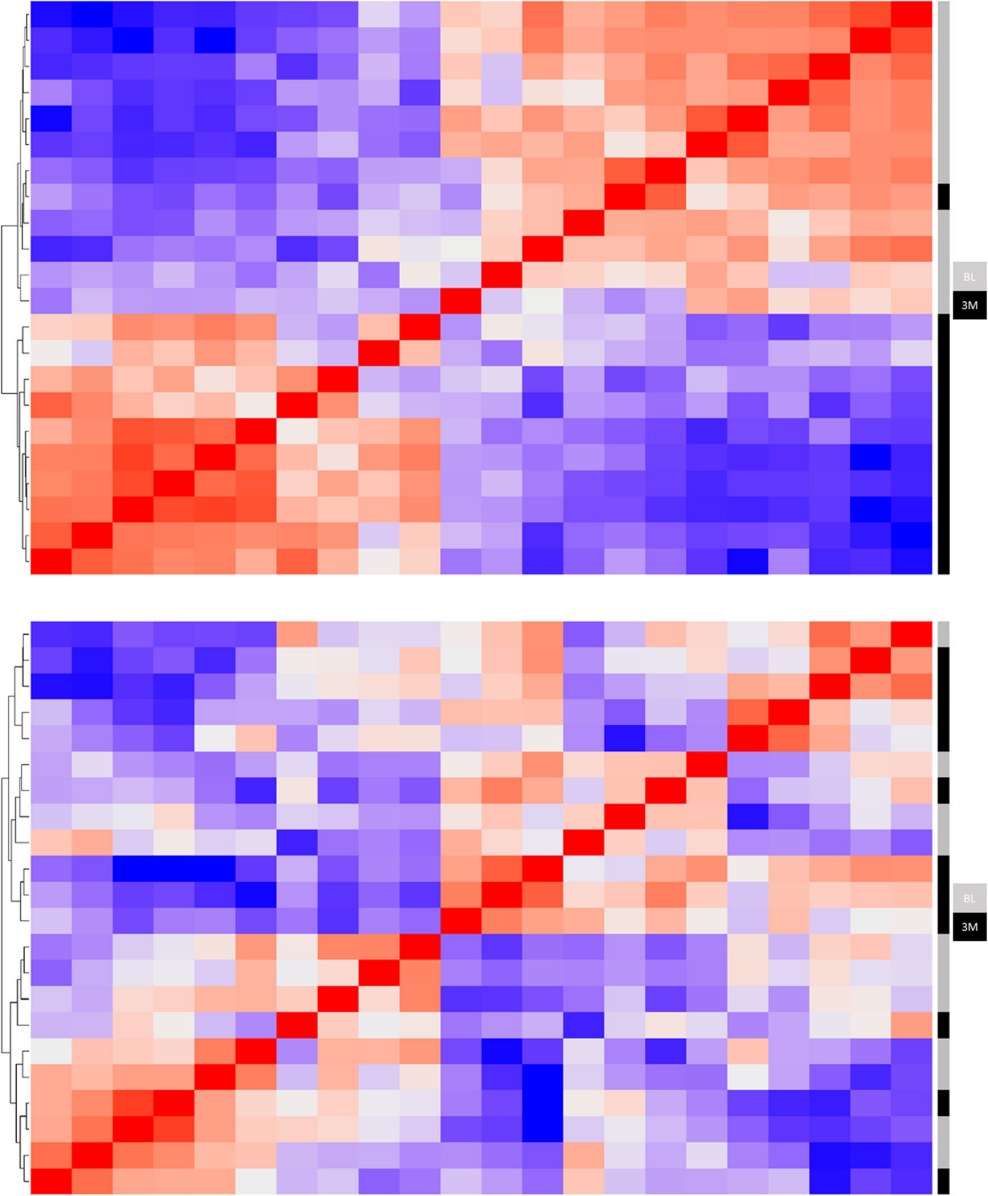
Heatmap of adalimumab good-responders and non-responders following real-time quantitative polymerase chain reaction (RT-qPCR) analysis of gene expression. Heatmap and hierarchal clustering is based on pairwise similarity in gene expression for the 104 transcripts measured in the RT-qPCR validation study. Good-responders and non-responders are represented in the upper and lower heatmaps, respectively. Heatmap rows and columns represent baseline and 3 months samples for 11 rheumatoid arthritis patients on adalimumab TNF-inhibitor therapy. BL = baseline and 3M = 3-months

A total of 11 good- and 11 non-responders were available to validate the top discovery results. Cohort characteristics were broadly similar to the discovery cohort with a minor difference in the mean baseline DAS28 (good-responders, mean = 6.27; non-responders, mean = 5.33; *p* = 0.0005). Relative quantification was used to compare expression between baseline and 3 months of adalimumab treatment for both response groups (Fig. 1). In good-responders differential expression was validated for 17 transcripts in the same direction of change as the initial discovery cohort (fold change > 1.2, FDR *p* < 0.05; Table 3). In the equivalent analysis of non-responders, no transcripts were differentially expressed at a significant level.

**Table 3:** Transcripts validated in adalimumab good-responders by real-time quantitative polymerase chain reaction (RT-QPCR)

Rank	Target name	Gene	Rq	Rq min	Rq max	Adjusted *p* value
1	Hs00190574_m1	*LIN7A*	0.69	0.59	0.8	0.01
2	Hs00191719_m1	*CREB5*	0.62	0.51	0.76	0.014
3	Hs00969559_m1	*ENTPD1*	0.79	0.71	0.88	0.019
4	Hs01565750_m1	*ITGB7*	1.51	1.25	1.82	0.02
5	Hs00185435_m1	*HLA-DMA*	1.34	1.17	1.52	0.023
6	Hs01075666_m1	*IL6R*	0.76	0.67	0.87	0.023
7	Hs01062258_m1	*SLC8A1*	0.68	0.59	0.79	0.025
8	Hs01555410_m1	*IL1B*	0.65	0.52	0.82	0.027
9	Hs00157950_m1	*HLA-DOB*	1.98	1.5	2.61	0.03
10	Hs01090216_m1	*MGAM*	0.56	0.41	0.76	0.03
11	Hs01072219_m1	*TRAF5*	1.47	1.27	1.69	0.031
12	Hs00171280_m1	*AES*	1.4	1.24	1.59	0.032
13	Hs00231092_m1	*E2F5*	1.61	1.26	2.07	0.035
14	Hs00207105_m1	*ZFYVE16*	0.8	0.71	0.9	0.037
15	Hs01109372_g1	*HLA-DOA*	1.63	1.3	2.03	0.037
16	Hs00152972_m1	*TLR8*	0.68	0.55	0.83	0.041
17	Hs00201585_m1	*STAP1*	1.51	1.22	1.86	0.042

The relative quantification (Rq) values are equivalent to linear fold changes for each transcript defined as the inverse log2 of the change in relative cycle threshold (∆∆Crt) values between pre-treatment and 3 months. Minimum and maximum RQ values and *p* value are presented for each transcript along with *p* values adjusted for multiple tests. Transcripts with an Rq value below 1 exhibited reduced expression at 3 months compared to pre-treatment. Transcripts with an Rq value above 1 exhibited increased expression at 3 months compared to pre-treatment

## Discussion

This study sought to identify biomarkers of adalimumab TNFi therapy. We observed a disease-relevant panel of transcriptomic changes in good-responders. In particular, significant differential expression was observed in good-responders between pre-treatment and 3 months, attributed to genes in RA-associated pathways that are responsive to relevant upstream regulators including TNF and CSF2 (otherwise known as Granulocyte-macrophage colony-stimulating factor (GM-CSF)), a promising target for therapy in RA [16].

Differential expression of seventeen transcripts observed in good-responders were validated in a small independent sample (*n* = 22) using OpenArray qPCR analysis. The validated transcripts were differentially expressed in the same direction as the initial discovery cohort and included *ENTPD1* (otherwise known as CD39), which is primarily expressed on activated lymphoid cells. A previous study reported an expansion of CD39 positive regulatory T cells following successful treatment with methotrexate [17], whilst a more recent study reported that a higher genetic score for CD39 expression on T cells at the *ENTPD1* locus was associated with a poor response to TNFi [18]. Here, we observed relatively higher levels of *ENTPD1* at pre-treatment in good-responders compared with the 3-month sample. However, in the current study, we cannot say whether the observed change in *ENTPD1* expression is reflected at the protein level, if a particular cell type is prominently affected or if expression is modified by genotype. Further studies of *ENTPD1* locus in relevant cell populations are now required in RA to resolve these interesting observations. In addition, this study identified increased expression of *CD40LG* in good-responders. The *CD40LG* gene encodes the complementary ligand for the CD40 transmembrane protein which is expressed on both B cells and antigen-presenting cells (APCs) and has been associated with increased TNF expression. Furthermore, transcription of both genes is reportedly elevated in the synovial tissue of RA patients [19]. More recently, increased CD40 transcription and lower CD40 methylation in whole blood were associated with improved TNFi response [18].

High expression of type I interferon genes has previously been associated with improved response to TNFi therapy [20] and, more recently, non-response to methotrexate therapy [21]. However, whilst expression of a number of interferon genes was initially associated with good-response in this study (*IFNG*, *IFNG-AS1*, *LY6E*, *MX2*, *SERPING1*, *OAS2*), only an association with *IFNG-AS1* was retained following adjustment for baseline DAS28 score and none were validated by RT-qPCR. Overall, this study identified many immune-related genes with increased expression in the whole blood of good-responders, whilst seemingly paradoxical, this may represent migration of RA-associated inflammatory factors out of the affected joint and into the peripheral blood in a positive response to TNFi therapy.

No differential expression was observed at pre-treatment when good- and non-responders were analysed, in keeping with previous results [8, 22]. One possible reason could be that immune pathways are saturated at baseline since patients included the study had very high disease activity at baseline. Only after treatment is administered do the different gene expression groups emerge. We found that good-responders showed enrichment in pathways linked to TNF, supporting the hypothesis that there are different key drivers in different subgroups of patients. In future studies, it will be important to test the differentially expressed transcripts identified herein against different drugs in order to elucidate whether they are general markers of response or specific to this class of drug. The inability to identify baseline markers suggests that further discovery studies should redirect efforts to other data types or include on-treatment sampling as part of the study design. It is possible that the high levels of disease activity seen at pre-treatment in all study participants may be obscuring detection of pre-treatment gene expression signatures that are relevant to future treatment response.

This study benefited from the use of a stringent sample selection process including good-responders in clinical remission with absence of anti-drug antibody or inadequate adherence explaining non-response. In addition, the use of a longitudinal approach has allowed the identification of transcripts and pathways that change in response to successful treatment. The signatures were observed in whole blood which requires fewer sample processing steps. However, this may dilute subtle changes that occur in cell types of low abundance that would otherwise be apparent in cell-specific studies. This is most likely pertinent to B and T cells, which this study has highlighted as important contributors to the transcriptomic response in good-responders. Whilst it would be interesting to identify the cell types in which these gene expression changes occur, to our knowledge, the reference datasets required to resolve cell subtypes from whole blood are not currently available for the array type used in this investigation.

We recognise that the initial discovery cohort contained more responders than non-responders, which may contribute towards a lack of power for finding significant changes between baseline and 3 months in non-responders. However, in order to maximise power and mimic a case-control approach, the replication phase examined an equal number of responders and non-responders. Furthermore, significant changes were identified only in the good-responder group, in keeping with findings in the discovery cohort. Whilst there was no statistically significant difference in concurrent DMARD use at baseline, we recognise that confounding could still occur and so adjusted for this during differential expression analysis.

Whilst 813 differentially expressed transcripts were found in responders between baseline and 3 months, no significant differences in gene expression were identified between responders and non-responders at 3 months. This is likely due to power and inter-patient differences in baseline gene expression levels. We also tested for correlation between change in transcript abundance and change in DAS28 and sub-components in the good-responder subgroup (Additional file 3: Figure S1–S4). In this unadjusted analysis, no significant *p* values were observed; however, the sample size was small. These analyses are based on good- and poor-EULAR response. Future studies should extend this analysis to include patients across the full spectrum of response, including intermediate responders, and analyse continuous measures of response, including the DAS28 composite score and its sub-components, as secondary outcomes.

We cannot exclude the possibility that other true associations were not detected in the replication phase of the study as the sample size was small, limiting power. Nonetheless, the findings support further testing of transcripts that have been independently replicated to develop a statistical model to stratify patients according to likely treatment response. Whilst the transcriptomic response was detected at 3 months, it is possible that changes in transcript levels could manifest much earlier. Furthermore, it will be important to test if these findings are specific to adalimumab response or generalise to other therapies licenced for use in RA. Future similar studies should therefore seek to collect samples at earlier time-points and from patients treated with other classes of therapy to address these important questions. Whilst the inclusion of the extremes of response phenotype in the current study maximised the power to detect associations, the inclusion of intermediate DAS28 responders in future studies will be necessary to allow an estimation of the predictive ability of the biomarker panel with DAS28 response across the full spectrum of response.

## Conclusions

This study has discovered biomarkers that could potentially be used to identify patients destined not to respond adequately to adalimumab treatment at an early stage and to support treat-to-target approaches. Whilst patients can be withdrawn from a therapy according to a limited change in DAS28 at 3 months, in reality, many patients remain on ineffective treatment for longer periods. Biomarkers could therefore aid clinical decisions to rapidly switch non-responder patients at 3 months or earlier to an alternative therapy. Much larger studies are now needed to test the utility of the identified biomarkers as a classifier of future response.

## Supplementary Information

**Additional file 1: Table S1.** Differentially expressed transcripts in adalimumab good-responders between baseline (pre-treatment) and 3-months of adalimumab treatment. The annotation for each probe was retrieved from the Affymetrix array manifest [MacDonald JW (2017). pd.hta.2.0: Platform Design Info for Affymetrix HTA-2_0. R package version 3.12.2]. Transcripts with a positive fold-change exhibited increased expression at 3-months compared to baseline. Transcripts with a negative fold-change exhibited reduced expression at 3-months compared to baseline.

**Additional file 2: Table S2.** The differentially expressed genes in adalimumab good-responders (baseline versus 3-months) as analysed using Ingenuity Pathway Analysis (IPA) software. The -log(*p*-value) for each pathway association, the ratio of the number of differentially expressed genes within each pathway, and a list of the differentially expressed genes (molecules) within each pathway are shown.

**Additional file 3: Figure S1.** Correlation between change in transcript expression level and change in DAS28 score in good responders for the top 10 differentially expressed transcripts according to *p*-value. **Figure S2.** Correlation between change in transcript expression level and change in swollen joint count (SJC) in good responders for the top 10 differentially expressed transcripts according to *p*-value. **Figure S3.** Correlation between change in transcript expression level and change in tender joint count (TJC) in good responders for the top 10 differentially expressed transcripts according to *p*-value. **Figure S4.** Correlation between change in transcript expression level and change in C-reactive protein (CRP) levels in good responders for the top 10 differentially expressed transcripts according to p-value.

## Data Availability

The datasets used and/or analysed during the current study are available from the corresponding author on reasonable request.

## Abbreviations

ACR: American College of Rheumatology
APC: Antigen-presenting cell
BRAGGSS: Biologics in Rheumatoid Arthritis Genetics and Genomics Study Syndicate
DAS28: 28-joint count disease activity score
DMARDs: Disease modifying anti-rheumatic drugs
EULAR: European League Against Rheumatism
FDR: False discovery rate
GM-CSF: Granulocyte-macrophage colony-stimulating factor
HTA: Human transcriptome array
IPA: Ingenuity Pathway Analysis
IQR: Interquartile range
LIMS: Laboratory information management system
PCA: Principal component analysis
RA: Rheumatoid arthritis
RIN: RNA integrity number
RQ: Relative quantification
RT-qPCR: Real-time quantitative polymerase chain reaction
SD: Standard deviation
TNF: Tumour necrosis factor
TNFi: TNF-inhibitor
∆∆Crt: Change in relative cycle threshold
