# Correction to: Transcriptome-wide study of TNF-inhibitor therapy in rheumatoid arthritis reveals early signature of successful treatment

**DOI:** 10.1186/s13075-021-02519-6

**Published:** 2021-05-08

**Authors:** James Oliver, Nisha Nair, Gisela Orozco, Samantha Smith, Kimme L. Hyrich, Ann Morgan, John Isaacs, Anthony G. Wilson, BRAGGSS, Anne Barton, Darren Plant

**Affiliations:** 1 Versus Arthritis Centre for Genetics and Genomics, Centre for Musculoskeletal Research, Manchester Academic Health Sciences Centre, The University of Manchester, Manchester, UK; 2 NIHR Manchester BRC, Manchester University Foundation Trust, Manchester, UK; 3 Versus Arthritis Centre for Epidemiology, Centre for Musculoskeletal Research, Manchester Academic Health Sciences Centre, The University of Manchester, Manchester, UK; 4 Leeds Institute of Rheumatic and Musculoskeletal Medicine, University of Leeds and NIHR Leeds Musculoskeletal Biomedical Research Unit, Leeds Teaching Hospitals NHS Trust, Leeds, UK; 5 Institute of Cellular Medicine, Newcastle University, Newcastle upon Tyne, UK; 6 National Institute for Health Research Newcastle Biomedical Research Centre at Newcastle upon Tyne Hospitals NHS Foundation Trust and Newcastle University, Newcastle upon Tyne, UK; 7 UCD School of Medicine and Medical Science, Conway Institute, University College Dublin, Dublin, Ireland

**Correction to: Arthritis Res Ther 23, 80 (2021)**

**https://doi.org/10.1186/s13075-021-02451-9**

Following publication of the original article [1], the authors reported an error in the spelling of the first author. The surname “Oliver” has been spelt with capital ‘I’ for India instead of an ‘l’ for Lima.

The original article has been corrected.
