# Supplementary material for: Transcriptome-wide study of TNF-inhibitor therapy in rheumatoid arthritis reveals early signature of successful treatment
Source: Arthritis Res Ther. 2021 Mar 10;23:80. doi: 10.1186/s13075-021-02451-9 (PMC7948368; doi:10.1186/s13075-021-02451-9)
Supplement: Supplementary file 1 — Additional file 1: Table S1. Differentially expressed transcripts in adalimumab good-responders between baseline (pre-treatment) and 3-months of adalimumab treatment. The annotation for each probe was retrieved from the Affymetrix array manifest [MacDonald JW (2017). pd.hta.2.0: Platform Design Info for Affymetrix HTA-2_0. R package version 3.12.2]. Transcripts with a positive fold-change exhibited increased expression at 3-months compared to baseline. Transcripts with a negative fold-change exhibited reduced expression at 3-months compared to baseline. [file 13075_2021_2451_MOESM1_ESM.pdf]

transcriptclusterid	Fold change	AveExpr	t	P.Value	adj.P.Val	B	probesetid	seqname	strand	start	stop	totalprobes	geneassignment	mrnaassignment
TC11001447.hg.1	-1.6	6.64	-4.66	7.46E-06	3.58E-04	3.49	TC11001447.	chr11	-	15593569	15643937	30		ENSEMBL
TC09001921.hg.1	-1.6	7.31	-4.31	3.09E-05	9.22E-04	2.17	TC09001921.	chr9	+	40332917	40337603	30		Broad
TC09001920.hg.1	-1.6	8.1	-4.08	7.65E-05	1.74E-03	1.33	TC09001920.	chr9	+	40308216	40324812	58		Broad
TC09000274.hg.1	-1.6	7.63	-4.1	6.99E-05	1.63E-03	1.42	TC09000274.	chr9	+	65647281	65664071	50	CNTP3B	GenBank
TC09001960.hg.1	-1.6	7.88	-4.28	3.47E-05	9.93E-04	2.06	TC09001960.	chr9	+	65629315	65635808	30		Broad
TC20000363.hg.1	-1.6	9.71	-6.57	9.48E-10	6.72E-06	11.9	TC20000363.	chr20	+	44637547	44645200	180	MMP9	RefSeq
TC07002629.hg.1	-1.6	7.25	-5.92	2.48E-08	1.54E-05	8.82	TC07002629.	chr7	+	141870970	141923474	280		Rinn
TC03003258.hg.1	-1.5	6.08	-4.73	5.48E-06	2.98E-04	3.77	TC03003258.	chr3	-	187553660	187574106	18		Rinn
TC01000272.hg.1	-1.5	9.31	-4.74	5.26E-06	2.93E-04	3.81	TC01000272.	chr1	+	21835858	21904905	185	ALPL	RefSeq
TC07000902.hg.1	-1.5	6.72	-5.81	4.20E-08	2.01E-05	8.33	TC07000902.	chr7	+	141811549	141921088	570	LOC100124692	RefSeq
TC09001097.hg.1	-1.5	8.17	-4.4	2.18E-05	7.26E-04	2.5	TC09001097.	chr9	-	40498481	40633261	80	CNTP3P2	GenBank
TC09002504.hg.1	-1.5	8.68	-4.31	3.14E-05	9.30E-04	2.16	TC09002504.	chr9	-	40501701	40503928	40		NONCODE
TC02003445.hg.1	-1.5	6.91	-5.44	2.36E-07	4.86E-05	6.71	TC02003445.	chr2	+	102661125	102674449	30		Rinn
TC09000231.hg.1	-1.5	6.05	-4.34	2.75E-05	8.49E-04	2.28	TC09000231.	chr9	+	40028620	40032417	30	AL353791.1	Havana
TC09001092.hg.1	-1.5	7.96	-4.41	2.08E-05	7.01E-04	2.54	TC09001092.	chr9	-	39072764	39288456	446	CNTP3	RefSeq
TC06000983.hg.1	-1.5	6.76	-4.88	2.84E-06	2.03E-04	4.39	TC06000983.	chr6	+	131894284	131905472	179	ARG1	RefSeq
TC04002881.hg.1	-1.5	7.83	-5.45	2.22E-07	4.70E-05	6.77	TC04002881.	chr4	-	185719451	185720200	30	SLED1	NONCODE
TC19002237.hg.1	-1.5	9.35	-5.94	2.22E-08	1.48E-05	8.93	TC19002237.	chr19	+	55396829	55401144	30	FCAR	NONCODE
TC07000899.hg.1	-1.5	10.14	-5.91	2.55E-08	1.54E-05	8.8	TC07000899.	chr7	+	141607613	141806547	844	MGAM	RefSeq
TC01003260.hg.1	-1.5	10.62	-5.73	6.14E-08	2.29E-05	7.97	TC01003260.	chr1	-	153346181	153348125	50	S100A12	RefSeq
TC15000927.hg.1	-1.5	6.03	-3.97	1.14E-04	2.35E-03	0.97	TC15000927.	chr15	+	94406338	94421585	80	LOC101927153	ENSEMBL
TC15001935.hg.1	-1.5	5.38	-5.13	9.67E-07	1.10E-04	5.39	TC15001935.	chr15	-	97316932	97316961	6		GenBank
TC06002119.hg.1	-1.5	7.45	-3.86	1.74E-04	3.23E-03	0.58	TC06002119.	chr6	-	133001997	133035194	110	VNN1	RefSeq
TC09000232.hg.1	-1.5	9	-4.56	1.14E-05	4.72E-04	3.1	TC09000232.	chr9	+	40311210	40311239	6		GenBank
TC04001810.hg.1	-1.5	7.84	-5.38	3.13E-07	5.69E-05	6.45	TC04001810.	chr4	-	185719450	185720200	30	SLED1	RefSeq
TC09001937.hg.1	-1.5	8.06	-4.42	1.97E-05	6.78E-04	2.59	TC09001937.	chr9	+	43685063	43890529	236		NONCODE
TC20000341.hg.1	-1.5	9.17	-3.8	2.14E-04	3.74E-03	0.39	TC20000341.	chr20	+	43803517	43805185	30	PI3	RefSeq
TC09001141.hg.1	-1.5	6.98	-4.18	5.08E-05	1.31E-03	1.71	TC09001141.	chr9	-	47295855	47314322	190	LOC643792	ENSEMBL
TC0X001610.hg.1	-1.5	7.23	-6.12	9.08E-09	1.26E-05	9.77	TC0X001610.	chrX	+	17168618	17171104	30	REPS2	NONCODE
TC04000437.hg.1	-1.5	7.9	-4.47	1.62E-05	5.95E-04	2.77	TC04000437.	chr4	+	79472673	79531605	233	ANXA3	RefSeq
TC09002501.hg.1	-1.5	8.81	-4.57	1.09E-05	4.57E-04	3.14	TC09002501.	chr9	-	39722426	39817486	40		Broad
TC02000619.hg.1	-1.5	7.66	-4.92	2.41E-06	1.83E-04	4.54	TC02000619.	chr2	+	102608306	102645006	200	IL1R2	RefSeq
TC0X000067.hg.1	-1.5	6.04	-5.84	3.66E-08	1.89E-05	8.46	TC0X000067.	chrX	+	15482369	15574652	257	BMX	RefSeq
TC07002628.hg.1	-1.5	6.33	-5.87	3.04E-08	1.74E-05	8.63	TC07002628.	chr7	+	141811549	141843783	130	LOC93432	NONCODE
TC15000928.hg.1	-1.4	5.33	-3.92	1.38E-04	2.70E-03	0.79	TC15000928.	chr15	+	94425311	94429972	30		ENSEMBL
TC09001919.hg.1	-1.4	6.96	-4.43	1.91E-05	6.65E-04	2.62	TC09001919.	chr9	+	40293022	40308427	60		Broad
TC09000253.hg.1	-1.4	7.52	-4.37	2.41E-05	7.75E-04	2.4	TC09000253.	chr9	+	43684885	43924049	440	CNTP3B	RefSeq
TC07002513.hg.1	-1.4	7.73	-4.38	2.32E-05	7.59E-04	2.44	TC07002513.	chr7	+	106065909	106151990	25		Rinn
TC12001253.hg.1	-1.4	8.06	-6.55	1.07E-09	6.72E-06	11.78	TC12001253.	chr12	-	12482198	12503475	110	MANSC1	RefSeq
TC12000130.hg.1	-1.4	7.85	-4.38	2.38E-05	7.71E-04	2.41	TC12000130.	chr12	+	8666136	8674962	60	CLEC4D	RefSeq
TC19000134.hg.1	-1.4	8.18	-4.87	3.03E-06	2.12E-04	4.33	TC19000134.	chr19	+	7741943	7744719	80	MCEMP1	RefSeq
TC07003054.hg.1	-1.4	7.9	-6.07	1.15E-08	1.43E-05	9.54	TC07003054.	chr7	-	87900207	87903065	30		NONCODE
TC02003322.hg.1	-1.4	5.65	-6.06	1.26E-08	1.43E-05	9.47	TC02003322.	chr2	+	71916293	71918581	30		NONCODE
TC05000097.hg.1	-1.4	6.97	-4.57	1.08E-05	4.54E-04	3.14	TC05000097.	chr5	+	17240720	17240824	30	RNU6-1003P	ENSEMBL
TC02000620.hg.1	-1.4	6.75	-5.74	5.86E-08	2.29E-05	8.02	TC02000620.	chr2	+	102681004	102796334	326	IL1R1	RefSeq
TC11002231.hg.1	-1.4	5.78	-2.86	4.96E-03	3.95E-02	-2.44	TC11002231.	chr11	-	102582526	102595685	174	MMP8	RefSeq
TC10000058.hg.1	-1.4	7.16	-4.37	2.44E-05	7.79E-04	2.39	TC10000058.	chr10	+	6392278	6394723	30	DKFZp667F0711	ENSEMBL

TC01003498.hg.1	-1.4	7.97	-5.1	1.12E-06	1.19E-04	5.26	TC01003498.	chr1	-	169481192	169555826	320	F5	RefSeq
TC02000937.hg.1	-1.4	8.09	-3.52	5.77E-04	7.75E-03	-0.51	TC02000937.	chr2	+	152214106	152236562	98	TNFAIP6	RefSeq
TC19001576.hg.1	-1.4	7.57	-3.03	2.96E-03	2.69E-02	-1.98	TC19001576.	chr19	-	43084393	43099082	78	CEACAM8	RefSeq
TC22000191.hg.1	-1.4	8.06	-4.39	2.21E-05	7.34E-04	2.48	TC22000191.	chr22	+	29469066	29564321	180	KREMEN1	RefSeq
TC05000724.hg.1	-1.4	6.75	-4.97	1.96E-06	1.64E-04	4.73	TC05000724.	chr5	+	139579748	139582772	30	CTB-131B5.2	Havana
TC05001068.hg.1	-1.4	5.29	-5.38	3.07E-07	5.62E-05	6.46	TC05001068.	chr5	+	180336838	180336927	30		ENSEMBL
TC15002385.hg.1	-1.4	6.75	-5.39	2.97E-07	5.60E-05	6.5	TC15002385.	chr15	+	94785816	94821492	30		NONCODE
TC04001809.hg.1	-1.4	10.6	-5.32	4.06E-07	6.61E-05	6.2	TC04001809.	chr4	-	185676749	185747972	344	ACSL1	RefSeq
TC16000494.hg.1	-1.4	9.71	-5.26	5.28E-07	8.00E-05	5.96	TC16000494.	chr16	+	57702157	57723975	160	GPR97	RefSeq
TC04001267.hg.1	-1.4	8.45	-5.54	1.48E-07	3.84E-05	7.15	TC04001267.	chr4	-	70592566	70653679	150	SULT1B1	RefSeq
TC06002274.hg.1	-1.4	5.66	-3.78	2.33E-04	3.97E-03	0.31	TC06002274.	chr6	-	160060339	160061133	30		ENSEMBL
TC07003216.hg.1	-1.4	5.92	-3.84	1.84E-04	3.36E-03	0.53	TC07003216.	chr7	-	142917561	142919360	30		NONCODE
TC07003292.hg.1	-1.4	6.13	-6.24	5.00E-09	1.18E-05	10.33	TC07003292.	chr7	+	112120908	112130943	160	LSMEM1	RefSeq
TC20000552.hg.1	-1.4	6.4	-6.03	1.41E-08	1.43E-05	9.35	TC20000552.	chr20	-	1509702	1509805	30	RNU6-917P	ENSEMBL
TC19002493.hg.1	-1.4	7.07	-2.96	3.65E-03	3.14E-02	-2.17	TC19002493.	chr19	-	43084396	43099066	70	CEACAM8	NONCODE
TC19000885.hg.1	-1.4	7.95	-6.17	7.34E-09	1.26E-05	9.97	TC19000885.	chr19	+	55385549	55403244	152	FCAR	RefSeq
TC14001125.hg.1	-1.4	6.19	-4.61	9.24E-06	4.08E-04	3.29	TC14001125.	chr14	-	51350294	51353947	30		ENSEMBL
TC06003963.hg.1	-1.4	5.65	-3.79	2.21E-04	3.81E-03	0.36	TC06003963.	chr6	-	160060339	160061133	30		Rinn
TC03000846.hg.1	-1.4	8.96	-5.31	4.25E-07	6.80E-05	6.16	TC03000846.	chr3	+	154741913	154901518	480	MME	RefSeq
TC02004178.hg.1	-1.4	5.72	-4.73	5.47E-06	2.98E-04	3.78	TC02004178.	chr2	-	40324916	40328505	30	SLC8A1	NONCODE
TC03001550.hg.1	-1.4	9.87	-4.88	2.94E-06	2.07E-04	4.36	TC03001550.	chr3	-	71820806	71834357	70	PROK2	RefSeq
TC01000232.hg.1	-1.4	8.83	-5.87	3.10E-08	1.76E-05	8.62	TC01000232.	chr1	+	17634690	17690499	220	PADI4	RefSeq
TC07002731.hg.1	-1.4	7.6	-5.02	1.61E-06	1.45E-04	4.92	TC07002731.	chr7	-	29246	31980	30		NONCODE
TC16000660.hg.1	-1.3	9.09	-5.11	1.08E-06	1.17E-04	5.29	TC16000660.	chr16	+	84853537	84943116	220	CRISPLD2	RefSeq
TC02000432.hg.1	-1.3	9.23	-5.56	1.36E-07	3.68E-05	7.23	TC02000432.	chr2	+	71680753	71913898	713	DYSF	RefSeq
TC02000237.hg.1	-1.3	8.24	-6.04	1.37E-08	1.43E-05	9.38	TC02000237.	chr2	+	37571717	37600465	170	QPCT	RefSeq
TC0X000082.hg.1	-1.3	8.95	-6.75	3.86E-10	5.23E-06	12.75	TC0X000082.	chrX	+	16964814	17171403	280	REPS2	RefSeq
TC06001269.hg.1	-1.3	6.69	-4.54	1.19E-05	4.86E-04	3.05	TC06001269.	chr6	-	10762956	10838788	216	MAK	RefSeq
TC01003677.hg.1	-1.3	6.73	-5.54	1.46E-07	3.81E-05	7.16	TC01003677.	chr1	-	200311672	200343482	80	LINC00862	RefSeq
TC09002907.hg.1	-1.3	6.51	-4.77	4.60E-06	2.71E-04	3.94	TC09002907.	chr9	+	117085303	117088759	130	ORM1	RefSeq
TC20000876.hg.1	-1.3	8.04	-4.21	4.57E-05	1.22E-03	1.81	TC20000876.	chr20	-	43880879	43883206	40	SLPI	RefSeq
TC12001774.hg.1	-1.3	5.1	-5.01	1.66E-06	1.48E-04	4.89	TC12001774.	chr12	-	81329515	81329612	30	MIR618	RefSeq
TC01001820.hg.1	-1.3	7.1	-6.33	3.32E-09	1.10E-05	10.72	TC01001820.	chr1	+	220960039	220987741	170	Mar-01	RefSeq
TC12003061.hg.1	-1.3	8.57	-5.22	6.55E-07	8.91E-05	5.76	TC12003061.	chr12	-	106631814	106632649	30		NONCODE
TC09001325.hg.1	-1.3	7.36	-6.17	7.04E-09	1.26E-05	10.01	TC09001325.	chr9	-	94171327	94186144	59	NFIL3	RefSeq
TC10002758.hg.1	-1.3	9.35	-4.93	2.31E-06	1.78E-04	4.58	TC10002758.	chr10	-	89832454	89840364	30		Rinn
TC05001587.hg.1	-1.3	6.83	-4.66	7.28E-06	3.52E-04	3.51	TC05001587.	chr5	-	90522292	90522888	30	RP11-414H23.3	Havana
TC06001669.hg.1	-1.3	6.71	-4.54	1.20E-05	4.87E-04	3.05	TC06001669.	chr6	-	35911291	35992645	330	SLC26A8	RefSeq
TC14001124.hg.1	-1.3	10.1	-5.95	2.10E-08	1.43E-05	8.98	TC14001124.	chr14	-	51324609	51411454	250	PYGL	RefSeq
TC10000629.hg.1	-1.3	6.82	-3.06	2.69E-03	2.50E-02	-1.9	TC10000629.	chr10	+	90519952	90537999	110	LIPN	RefSeq
TC01006029.hg.1	-1.3	6.29	-5.45	2.22E-07	4.70E-05	6.77	TC01006029.	chr1	-	200298333	200343319	89	LINC00862	NONCODE
TC03002534.hg.1	-1.3	7.34	-5.68	7.79E-08	2.63E-05	7.75	TC03002534.	chr3	+	128949393	128955149	40		Rinn
TC01000008.hg.1	-1.3	9.35	-4.9	2.67E-06	1.97E-04	4.45	TC01000008.	chr1	+	334140	342806	30	LOC101928706	ENSEMBL
TC10000258.hg.1	-1.3	10.01	-4.81	3.85E-06	2.45E-04	4.1	TC10000258.	chr10	+	38712553	38712656	30	RNU6-1118P	ENSEMBL
TC04001138.hg.1	-1.3	6.5	-3.97	1.14E-04	2.34E-03	0.97	TC04001138.	chr4	-	40504057	40504136	30	MIR4802	RefSeq
TC01002055.hg.1	-1.3	10.01	-4.8	4.04E-06	2.52E-04	4.06	TC01002055.	chr1	-	157784	157887	30	RNU6-1100P	ENSEMBL
TC01003865.hg.1	-1.3	10.01	-4.8	4.04E-06	2.52E-04	4.06	TC01003865.	chr1	-	222676974	222677077	30	RNU6-791P	ENSEMBL
TC04001502.hg.1	-1.3	10.01	-4.8	4.04E-06	2.52E-04	4.06	TC04001502.	chr4	-	120355160	120355263	30	RNU6-1217P	ENSEMBL

TC09000858.hg.1	-1.3	10.01	-4.8	4.04E-06	2.52E-04	4.06	TC09000858.	chr9	+	141150045	141150148	30	RNU6-785P	ENSEMBL
TC16000715.hg.1	-1.3	10.01	-4.8	4.04E-06	2.52E-04	4.06	TC16000715.	chr16	+	90211000	90211103	30	RNU6-355P	ENSEMBL
TC19000980.hg.1	-1.3	10.01	-4.8	4.04E-06	2.52E-04	4.06	TC19000980.	chr19	-	223158	223261	30	RNU6-1076P	ENSEMBL
TC11001227.hg.1	-1.3	10.01	-4.8	4.11E-06	2.54E-04	4.04	TC11001227.	chr11	-	152999	153102	30	RNU6-447P	ENSEMBL
TC11000812.hg.1	-1.3	9.31	-5.97	1.96E-08	1.43E-05	9.05	TC11000812.	chr11	+	75479777	75512581	120	DGAT2	RefSeq
TC01000261.hg.1	-1.3	9.46	-5.8	4.37E-08	2.06E-05	8.29	TC01000261.	chr1	+	20915441	20945401	78	CDA	RefSeq
TC05001087.hg.1	-1.3	9.16	-4.73	5.55E-06	3.00E-04	3.76	TC05001087.	chr5	+	180728968	180729071	30	RNU6-705P	ENSEMBL
TC04000776.hg.1	-1.3	6.05	-4.08	7.46E-05	1.71E-03	1.36	TC04000776.	chr4	+	154641479	154648874	50	RP11-153M7.5	Havana
TC13001678.hg.1	-1.3	3.95	-3.56	5.12E-04	7.09E-03	-0.4	TC13001678.	chr13	-	110053200	110054833	28		Rinn
TC14002079.hg.1	-1.3	8.57	-5.1	1.10E-06	1.18E-04	5.27	TC14002079.	chr14	-	73925556	73932873	82		Rinn
TC09001263.hg.1	-1.3	3.97	-4.32	2.93E-05	8.89E-04	2.22	TC09001263.	chr9	-	84534684	84534718	11		GenBank
TC09001269.hg.1	-1.3	3.97	-4.32	2.93E-05	8.89E-04	2.22	TC09001269.	chr9	-	84549756	84549790	11		GenBank
TC12001772.hg.1	-1.3	8.15	-6.48	1.55E-09	8.73E-06	11.43	TC12001772.	chr12	-	81186299	81331704	120	LIN7A	RefSeq
TC04000826.hg.1	-1.3	8.03	-5.38	3.06E-07	5.62E-05	6.47	TC04000826.	chr4	+	166128770	166244308	310	KLHL2	RefSeq
TC01003974.hg.1	-1.3	7.57	-4.67	7.00E-06	3.43E-04	3.55	TC01003974.	chr1	-	232533711	232697304	330	SIPA1L2	RefSeq
TC03002096.hg.1	-1.3	10.54	-5.33	3.93E-07	6.49E-05	6.23	TC03002096.	chr3	-	187439165	187463515	243	BCL6	RefSeq
TC12002978.hg.1	-1.3	7.37	-5.48	1.99E-07	4.45E-05	6.87	TC12002978.	chr12	-	81186299	81189685	30		NONCODE
TC10001860.hg.1	-1.3	9.87	-4.5	1.45E-05	5.53E-04	2.87	TC10001860.	chr10	+	6266130	6275053	30	PFKFB3	NONCODE
TC05003140.hg.1	-1.3	7.18	-4.54	1.22E-05	4.92E-04	3.03	TC05003140.	chr5	-	90538753	90560495	30		Rinn
TC12002425.hg.1	-1.3	9.03	-4.72	5.67E-06	3.03E-04	3.74	TC12002425.	chr12	+	66645119	66647711	30	IRAK3	NONCODE
TC01002071.hg.1	-1.3	9.6	-4.76	4.80E-06	2.78E-04	3.9	TC01002071.	chr1	-	693613	693716	30	RNU6-1199P	ENSEMBL
TC06002120.hg.1	-1.3	8.13	-4.51	1.40E-05	5.39E-04	2.9	TC06002120.	chr6	-	133043923	133055904	174	VNN3	RefSeq
TC12001842.hg.1	-1.3	8.57	-5.11	1.07E-06	1.16E-04	5.3	TC12001842.	chr12	-	96366440	96390143	274	HAL	RefSeq
TC20000726.hg.1	-1.3	8.87	-6.16	7.63E-09	1.26E-05	9.93	TC20000726.	chr20	-	24943561	24973615	120	APMAP	RefSeq
TC05000633.hg.1	-1.3	7.83	-5.33	4.00E-07	6.56E-05	6.22	TC05000633.	chr5	+	131630136	131679899	140	SLC22A4	RefSeq
TC01005541.hg.1	-1.3	7.27	-5.37	3.31E-07	5.89E-05	6.39	TC01005541.	chr1	-	65450881	65451399	29		Broad
TC20001235.hg.1	-1.3	9	-3.77	2.43E-04	4.09E-03	0.28	TC20001235.	chr20	+	43808589	43821571	82		Rinn
TC08000873.hg.1	-1.3	8.79	-4.59	9.77E-06	4.24E-04	3.24	TC08000873.	chr8	-	142091	150563	30		ENSEMBL
TC19000274.hg.1	-1.3	9.01	-5.6	1.11E-07	3.24E-05	7.42	TC19000274.	chr19	+	15751695	15773634	235	CYP4F3	RefSeq
TC18000436.hg.1	-1.3	7.79	-3.72	2.86E-04	4.59E-03	0.13	TC18000436.	chr18	-	28645940	28682388	240	DSC2	RefSeq
TC10000057.hg.1	-1.3	6.93	-4.9	2.65E-06	1.96E-04	4.45	TC10000057.	chr10	+	6319650	6377938	70	LOC399715	RefSeq
TC15000930.hg.1	-1.3	8.72	-5.43	2.54E-07	5.02E-05	6.64	TC15000930.	chr15	+	94774767	95027181	414	MCTP2	RefSeq
TC04001355.hg.1	-1.3	8.84	-5.14	9.44E-07	1.10E-04	5.41	TC04001355.	chr4	-	85590693	85887544	888	WDFY3	RefSeq
TC07000669.hg.1	-1.3	7	-4.5	1.45E-05	5.53E-04	2.87	TC07000669.	chr7	+	102613969	102629303	40	NFE4	ENSEMBL
TC07001582.hg.1	-1.3	9.72	-5.49	1.87E-07	4.29E-05	6.93	TC07001582.	chr7	-	87900207	87936228	90	STEAP4	RefSeq
TC09000344.hg.1	-1.3	6.48	-5.01	1.68E-06	1.49E-04	4.88	TC09000344.	chr9	+	77812987	77813093	30	RNU6-1228P	ENSEMBL
TC12002238.hg.1	-1.3	4.11	-6.27	4.48E-09	1.18E-05	10.44	TC12002238.	chr12	+	10393135	10412929	30		Rinn
TC12002078.hg.1	-1.3	9.03	-5.15	8.74E-07	1.06E-04	5.49	TC12002078.	chr12	-	123185840	123187904	40	HCAR2	RefSeq
TC09002505.hg.1	-1.3	8.28	-4.53	1.28E-05	5.08E-04	2.99	TC09002505.	chr9	-	40610094	40633391	30		NONCODE
TC07002504.hg.1	-1.3	6.78	-4.28	3.47E-05	9.94E-04	2.06	TC07002504.	chr7	+	102613969	102629303	40	NFE4	NONCODE
TC15000441.hg.1	-1.3	11.01	-5.68	7.79E-08	2.63E-05	7.75	TC15000441.	chr15	+	58430368	58478110	134	AQP9	RefSeq
TC04000606.hg.1	-1.3	9.71	-4.44	1.81E-05	6.42E-04	2.67	TC04000606.	chr4	+	119527605	119527708	30	RNU6-1054P	ENSEMBL
TC19001640.hg.1	-1.3	7.79	-5.26	5.50E-07	8.12E-05	5.92	TC19001640.	chr19	-	46522412	46526556	50	PGLYRP1	RefSeq
TC06003619.hg.1	-1.3	9.04	-5.07	1.28E-06	1.28E-04	5.13	TC06003619.	chr6	-	35800811	35888957	152	SRPK1	NONCODE
TC10001861.hg.1	-1.3	6.89	-4.54	1.21E-05	4.89E-04	3.04	TC10001861.	chr10	+	6319650	6377937	120	LOC399715	NONCODE
TC09001435.hg.1	-1.3	5.26	-5.36	3.39E-07	5.89E-05	6.37	TC09001435.	chr9	-	107606051	107606603	30		GenBank
TC12000745.hg.1	-1.3	5.61	-4.3	3.19E-05	9.42E-04	2.14	TC12000745.	chr12	+	96419101	96421749	30		ENSEMBL
TC17000736.hg.1	-1.3	6.77	-6.71	4.65E-10	5.23E-06	12.57	TC17000736.	chr17	+	58227297	58248260	110	CA4	RefSeq

TC06003854.hg.1	-1.3	8.74	-4.47	1.63E-05	5.97E-04	2.76	TC06003854.	chr6	-	133043926	133055904	70	VNN3	NONCODE
TC06001668.hg.1	-1.3	8.22	-5.26	5.32E-07	8.01E-05	5.95	TC06001668.	chr6	-	35800743	35889119	364	SRPK1	RefSeq
TC05001601.hg.1	-1.3	7.08	-4.62	8.66E-06	3.92E-04	3.35	TC05001601.	chr5	-	93486556	93954309	302	KIAA0825	RefSeq
TC11003373.hg.1	-1.3	8.03	-4.76	4.87E-06	2.80E-04	3.88	TC11003373.	chr11	-	118097406	118099840	30		NONCODE
TC05003444.hg.1	-1.3	8.81	-4.82	3.77E-06	2.43E-04	4.12	TC05003444.	chr5	+	180756242	180769214	30	AC138035.1	Havana
TC12000591.hg.1	-1.3	9.31	-5	1.74E-06	1.53E-04	4.84	TC12000591.	chr12	+	66582659	66648402	183	IRAK3	RefSeq
TC04000248.hg.1	-1.3	5.85	-4.45	1.75E-05	6.27E-04	2.7	TC04000248.	chr4	+	40751914	40812002	220	NSUN7	RefSeq
TC01005194.hg.1	-1.3	8.92	-4.65	7.78E-06	3.67E-04	3.45	TC01005194.	chr1	-	684275	703869	100	RP11-206L10.2	NONCODE
TC08001996.hg.1	-1.3	4.34	-4.19	4.90E-05	1.28E-03	1.74	TC08001996.	chr8	+	82066546	82075134	50		Rinn
TC02002219.hg.1	-1.3	8.5	-5.31	4.24E-07	6.80E-05	6.16	TC02002219.	chr2	-	113587328	113594480	197	IL1B	RefSeq
TC05000983.hg.1	-1.3	7.5	-4.3	3.16E-05	9.33E-04	2.15	TC05000983.	chr5	+	175130901	175131007	30	RNU6-226P	ENSEMBL
TC20000928.hg.1	-1.3	9.75	-5.37	3.32E-07	5.89E-05	6.39	TC20000928.	chr20	-	48249482	48330421	120	B4GALT5	RefSeq
TC08001654.hg.1	-1.3	6.96	-4.12	6.54E-05	1.55E-03	1.48	TC08001654.	chr8	-	133584320	133687838	267	LRRC6	RefSeq
TC16000098.hg.1	-1.3	8.84	-5.48	1.95E-07	4.41E-05	6.89	TC16000098.	chr16	+	3096682	3110724	140	MMP25	RefSeq
TC15001837.hg.1	-1.3	10.58	-5.46	2.18E-07	4.67E-05	6.78	TC15001837.	chr15	-	90328120	90358094	230	ANPEP	RefSeq
TC12001027.hg.1	-1.3	9.94	-5.76	5.29E-08	2.18E-05	8.11	TC12001027.	chr12	+	129337979	129469509	170	GLT1D1	RefSeq
TC07002222.hg.1	-1.3	10.74	-5.47	2.04E-07	4.49E-05	6.85	TC07002222.	chr7	+	28863333	28865505	30	CREB5	NONCODE
TC07002730.hg.1	-1.3	9.24	-5.25	5.69E-07	8.23E-05	5.89	TC07002730.	chr7	-	26392	35472	30		Rinn
TC02003748.hg.1	-1.3	7.72	-4.57	1.08E-05	4.54E-04	3.14	TC02003748.	chr2	+	192556795	192583257	110		NONCODE
TC07000405.hg.1	-1.3	7.15	-4.21	4.51E-05	1.21E-03	1.82	TC07000405.	chr7	+	65670186	65885530	149	TPST1	RefSeq
TC07001067.hg.1	-1.3	9.88	-4.94	2.26E-06	1.77E-04	4.6	TC07001067.	chr7	-	19757	35479	40		ENSEMBL
TC06000699.hg.1	-1.3	4.52	-5.73	5.99E-08	2.29E-05	8	TC06000699.	chr6	+	64516729	64532535	30	RP11-59D5__B.2	Havana
TC05000075.hg.1	-1.3	7.46	-6.01	1.62E-08	1.43E-05	9.23	TC05000075.	chr5	+	10441636	10472141	133	ROPN1L	RefSeq
TC10000053.hg.1	-1.3	8.4	-4.82	3.81E-06	2.44E-04	4.11	TC10000053.	chr10	+	6186843	6277507	325	PFKFB3	RefSeq
TC03000423.hg.1	-1.3	7.66	-6.03	1.41E-08	1.43E-05	9.36	TC03000423.	chr3	+	71803201	71805647	30	GPR27	RefSeq
TC01001738.hg.1	-1.3	10.48	-4.75	5.08E-06	2.87E-04	3.84	TC01001738.	chr1	+	207669473	207815110	560	CR1	RefSeq
TC07001920.hg.1	-1.3	7.52	-5.56	1.36E-07	3.68E-05	7.23	TC07001920.	chr7	-	139993493	140104233	495	SLC37A3	RefSeq
TC12001179.hg.1	-1.3	7.32	-2.74	6.88E-03	5.06E-02	-2.73	TC12001179.	chr12	-	8700957	8720209	30		ENSEMBL
TC05001641.hg.1	-1.3	8.95	-4.13	6.19E-05	1.49E-03	1.53	TC05001641.	chr5	-	101569690	101632253	150	SLCO4C1	RefSeq
TC19001241.hg.1	-1.3	10.35	-6.02	1.51E-08	1.43E-05	9.29	TC19001241.	chr19	-	14729929	14785730	190	EMR3	RefSeq
TC01005706.hg.1	-1.3	8.07	-5.15	8.80E-07	1.06E-04	5.48	TC01005706.	chr1	-	112265426	112268060	30	FAM212B	NONCODE
TC07001714.hg.1	-1.3	6.16	-4	1.03E-04	2.16E-03	1.06	TC07001714.	chr7	-	102453308	102715288	335	FBXL13	RefSeq
TC20001489.hg.1	-1.3	7.83	-3.42	8.30E-04	1.02E-02	-0.84	TC20001489.	chr20	-	23052184	23053359	30		NONCODE
TC08002537.hg.1	-1.3	9.19	-3.85	1.83E-04	3.35E-03	0.53	TC08002537.	chr8	-	133697316	133699876	27		Rinn
TC01003878.hg.1	-1.3	9.21	-4.57	1.08E-05	4.53E-04	3.15	TC01003878.	chr1	-	224163848	224163951	30	RNU6-1319P	ENSEMBL
TC01004037.hg.1	-1.3	9.21	-4.57	1.08E-05	4.53E-04	3.15	TC01004037.	chr1	-	243244458	243244561	30	RNU6-747P	ENSEMBL
TC07000284.hg.1	-1.3	9.21	-4.57	1.08E-05	4.53E-04	3.15	TC07000284.	chr7	+	45829184	45829287	30	RNU6-241P	ENSEMBL
TC08000908.hg.1	-1.3	6.43	-2.91	4.27E-03	3.53E-02	-2.31	TC08000908.	chr8	-	6793344	6795860	60	DEFA4	RefSeq
TC01005186.hg.1	-1.3	9.04	-4.72	5.78E-06	3.07E-04	3.72	TC01005186.	chr1	-	154268	163727	70	RP11-34P13.13	NONCODE
TC06004062.hg.1	-1.3	8.28	-5.68	7.88E-08	2.64E-05	7.74	TC06004062.	chr6	+	2994232	3019996	150	NQO2	RefSeq
TC04001442.hg.1	-1.3	6.39	-3.83	1.96E-04	3.50E-03	0.47	TC04001442.	chr4	-	106058437	106061776	30	LOC101929491	ENSEMBL
TC05001766.hg.1	-1.3	8.11	-5.84	3.60E-08	1.88E-05	8.48	TC05001766.	chr5	-	131755108	131762406	40	AC116366.5	Havana
TC09001434.hg.1	-1.3	7.67	-4.33	2.90E-05	8.84E-04	2.23	TC09001434.	chr9	-	107543283	107690527	579	ABCA1	RefSeq
TC20001380.hg.1	-1.3	4.87	-4.82	3.81E-06	2.44E-04	4.11	TC20001380.	chr20	-	1732662	1746252	40	RP5-968J1.1	NONCODE
TC04001356.hg.1	-1.3	7.04	-4.85	3.29E-06	2.24E-04	4.25	TC04001356.	chr4	-	85807539	85807639	30	RNU6-469P	ENSEMBL
TC02001300.hg.1	-1.3	9.61	-5.72	6.52E-08	2.36E-05	7.92	TC02001300.	chr2	+	219246752	219261617	446	SLC11A1	RefSeq
TC01002419.hg.1	-1.3	8.41	-5.2	6.95E-07	9.12E-05	5.7	TC01002419.	chr1	-	28469248	28469352	30	RNU6-176P	ENSEMBL
TC06001785.hg.1	-1.3	5.56	-3.62	4.12E-04	6.09E-03	-0.21	TC06001785.	chr6	-	49695092	49712168	128	CRISP3	RefSeq

TC07000776.hg.1	-1.3	7.99	-4.67	6.99E-06	3.43E-04	3.55	TC07000776.	chr7	+	128267226	128267329	30	RNU6-177P	ENSEMBL
TC02004035.hg.1	-1.3	6.74	-4.8	4.13E-06	2.55E-04	4.04	TC02004035.	chr2	-	8992820	8994376	30	MBOAT2	NONCODE
TC12001170.hg.1	-1.3	10.35	-5.39	2.95E-07	5.58E-05	6.5	TC12001170.	chr12	-	8071824	8088892	240	SLC2A3	RefSeq
TC19000788.hg.1	-1.3	8.44	-4.62	8.69E-06	3.93E-04	3.35	TC19000788.	chr19	+	52264183	52273779	59	FPR2	RefSeq
TC01004862.hg.1	-1.3	5.29	-5.04	1.43E-06	1.37E-04	5.03	TC01004862.	chr1	+	180100864	180103986	21		Rinn
TC17001903.hg.1	-1.3	8.68	-5.42	2.59E-07	5.07E-05	6.62	TC17001903.	chr17	-	74561461	74582145	120	ST6GALC2	RefSeq
TC02004438.hg.1	-1.3	5.67	-3.77	2.40E-04	4.06E-03	0.29	TC02004438.	chr2	-	101845073	101851475	35		NONCODE
TC10002530.hg.1	-1.3	8.42	-4.83	3.65E-06	2.38E-04	4.15	TC10002530.	chr10	-	29933707	29935702	30		NONCODE
TC08001844.hg.1	-1.3	7.95	-4.89	2.82E-06	2.03E-04	4.39	TC08001844.	chr8	+	25272426	25273231	30		NONCODE
TC20001393.hg.1	-1.3	9.99	-5.14	9.41E-07	1.10E-04	5.42	TC20001393.	chr20	-	3907958	3911562	30		NONCODE
TC11002997.hg.1	-1.3	6.79	-5.83	3.71E-08	1.89E-05	8.45	TC11002997.	chr11	-	1792623	1793111	30		Rinn
TC01006030.hg.1	-1.3	6.72	-4.94	2.22E-06	1.75E-04	4.61	TC01006030.	chr1	-	200374083	200375228	30	ZNF281	NONCODE
TC06003141.hg.1	-1.3	6.42	-4.28	3.49E-05	9.96E-04	2.06	TC06003141.	chr6	+	155569226	155575172	60	TIAM2	NONCODE
TC01004955.hg.1	-1.3	5.72	-4.15	5.77E-05	1.42E-03	1.59	TC01004955.	chr1	+	202530389	202530835	30		NONCODE
TC07000406.hg.1	-1.3	4.42	-3.04	2.85E-03	2.62E-02	-1.95	TC07000406.	chr7	+	65809291	65809401	30	RNU6-313P	ENSEMBL
TC06002755.hg.1	-1.3	6.54	-4.07	7.80E-05	1.76E-03	1.32	TC06002755.	chr6	+	36084167	36091301	23		Rinn
TC11002689.hg.1	-1.3	8.78	-5.11	1.06E-06	1.16E-04	5.31	TC11002689.	chr11	+	59570202	59573350	30	STX3	NONCODE
TC08001264.hg.1	-1.3	6.21	-4.85	3.31E-06	2.24E-04	4.24	TC08001264.	chr8	-	62413115	62627199	689	ASPH	RefSeq
TC06003643.hg.1	-1.3	7.29	-4.73	5.50E-06	2.99E-04	3.77	TC06003643.	chr6	-	41215572	41216548	40	ADCY10P1	NONCODE
TC02000412.hg.1	-1.3	9.4	-4.47	1.60E-05	5.92E-04	2.78	TC02000412.	chr2	+	70157400	70160078	30		UCSC
TC22000224.hg.1	-1.3	8.91	-4.98	1.91E-06	1.62E-04	4.76	TC22000224.	chr22	+	31608225	31676066	320	LIMK2	RefSeq
TC21000718.hg.1	-1.3	9.96	-3.02	3.04E-03	2.75E-02	-2.01	TC21000718.	chr21	+	39610955	39617014	30	BRWD1-AS1	NONCODE
TC14000781.hg.1	-1.3	8.54	-5.69	7.54E-08	2.59E-05	7.78	TC14000781.	chr14	+	102829299	102968818	319	TECPR2	RefSeq
TC19002672.hg.1	-1.3	8.18	-5.27	5.23E-07	7.97E-05	5.97	TC19002672.	chr19	-	4537227	4540092	60	LRG1	RefSeq
TC01002287.hg.1	-1.3	9.16	-3.83	1.94E-04	3.48E-03	0.48	TC01002287.	chr1	-	17393256	17445948	240	PADI2	RefSeq
TC01000014.hg.1	-1.3	6.18	-4.48	1.57E-05	5.84E-04	2.8	TC01000014.	chr1	+	696291	697369	30	RP11-206L10.4	Havana
TC02003610.hg.1	-1.3	7	-5.15	8.96E-07	1.07E-04	5.46	TC02003610.	chr2	+	139362080	139370737	30		Rinn
TC01004100.hg.1	-1.3	5.73	-4.1	7.07E-05	1.64E-03	1.41	TC01004100.	chr1	+	160446	161525	30		Rinn
TC01000004.hg.1	-1.3	6.22	-4.06	8.18E-05	1.83E-03	1.27	TC01000004.	chr1	+	160446	161525	30	RP11-34P13.9	Havana
TC12002520.hg.1	-1.3	10.33	-5.95	2.10E-08	1.43E-05	8.98	TC12002520.	chr12	+	94656297	94701451	118	PLXNC1	NONCODE
TC01004103.hg.1	-1.3	9.23	-4.69	6.49E-06	3.32E-04	3.62	TC01004103.	chr1	+	329784	342806	32		Broad
TC20000553.hg.1	-1.3	8.1	-6.74	3.99E-10	5.23E-06	12.71	TC20000553.	chr20	-	1514897	1539489	70	SIRPD	RefSeq
TC03002601.hg.1	-1.3	5.98	-5.13	9.70E-07	1.10E-04	5.39	TC03002601.	chr3	+	150608647	150610167	30		Rinn
TC01001882.hg.1	-1.3	10.09	-4.68	6.89E-06	3.41E-04	3.56	TC01001882.	chr1	+	228160169	228160213	21		GenBank
TC01003855.hg.1	-1.3	10.09	-4.68	6.89E-06	3.41E-04	3.56	TC01003855.	chr1	-	222646723	222646767	21	LOC100132062	GenBank
TC10000547.hg.1	-1.3	8.08	-4.62	8.86E-06	3.97E-04	3.33	TC10000547.	chr10	+	79540986	79541016	7		GenBank
TC02003274.hg.1	-1.3	9.15	-4.39	2.27E-05	7.48E-04	2.45	TC02003274.	chr2	+	64501019	64550940	30		Rinn
TC19001261.hg.1	-1.3	7.59	-6.02	1.48E-08	1.43E-05	9.31	TC19001261.	chr19	-	15988834	16008884	160	CYP4F2	RefSeq
TC06001717.hg.1	-1.3	10.11	-4.49	1.49E-05	5.62E-04	2.84	TC06001717.	chr6	-	41242999	41254457	64	TREM1	RefSeq
TC02000895.hg.1	-1.3	7.07	-5.06	1.33E-06	1.30E-04	5.09	TC02000895.	chr2	+	139362080	139370737	30	AC092620.3	Havana
TC05000984.hg.1	-1.3	9.14	-4.47	1.65E-05	6.04E-04	2.75	TC05000984.	chr5	+	175136606	175137759	30		Havana
TC10001562.hg.1	-1.3	9.42	-6.03	1.43E-08	1.43E-05	9.35	TC10001562.	chr10	-	99092254	99094466	30	FRAT2	RefSeq
TC20001490.hg.1	-1.3	8.84	-3.7	3.12E-04	4.92E-03	0.05	TC20001490.	chr20	-	23053705	23061258	40	LINC00656	NONCODE
TC13001428.hg.1	-1.3	4.91	-5.88	2.92E-08	1.71E-05	8.67	TC13001428.	chr13	-	37717104	37720855	30		NONCODE
TC06003006.hg.1	-1.3	8.3	-4.91	2.56E-06	1.91E-04	4.48	TC06003006.	chr6	+	114178524	114182980	80	MARCKS	NONCODE
TC0X002222.hg.1	-1.3	4.24	-3.75	2.57E-04	4.26E-03	0.22	TC0X002222.	chrX	-	123509753	123510852	30	TENM1	NONCODE
TC02003749.hg.1	-1.3	8.6	-4.01	9.88E-05	2.11E-03	1.1	TC02003749.	chr2	+	192559982	192563100	30		Rinn
TC12000730.hg.1	-1.3	10.32	-5.98	1.87E-08	1.43E-05	9.09	TC12000730.	chr12	+	94542499	94701451	378	PLXNC1	RefSeq

TC10002842.hg.1	-1.3	6.16	-3.82	2.01E-04	3.57E-03	0.45	TC10002842.	chr10	-	116579688	116581199	23		Rinn
TC06000909.hg.1	-1.3	8.68	-4.95	2.14E-06	1.73E-04	4.65	TC06000909.	chr6	+	114178527	114184652	60	MARCKS	RefSeq
TC06001205.hg.1	-1.3	8.65	-4.94	2.24E-06	1.76E-04	4.61	TC06001205.	chr6	-	131910	144885	30	LINC00266-3	ENSEMBL
TC07000252.hg.1	-1.3	5.75	-4.67	7.10E-06	3.46E-04	3.53	TC07000252.	chr7	+	39807940	39808043	30	RNU6-719P	ENSEMBL
TC09002363.hg.1	-1.3	8.4	-4.62	8.67E-06	3.92E-04	3.35	TC09002363.	chr9	+	141139721	141142607	30		Rinn
TC02001910.hg.1	-1.3	4.48	-4.71	6.04E-06	3.16E-04	3.68	TC02001910.	chr2	-	64313484	64315380	30	AC012368.1	Havana
TC21000168.hg.1	-1.3	11.1	-3.77	2.40E-04	4.06E-03	0.29	TC21000168.	chr21	+	39645398	39647443	30		GenBank
TC21000167.hg.1	-1.3	9.56	-3.82	2.01E-04	3.57E-03	0.45	TC21000167.	chr21	+	39628663	39673748	240	KCNJ15	RefSeq
TC04002305.hg.1	-1.3	6.46	-5.07	1.27E-06	1.27E-04	5.14	TC04002305.	chr4	+	166143201	166144207	30		NONCODE
TC07003322.hg.1	-1.3	6.42	-4.95	2.17E-06	1.74E-04	4.64	TC07003322.	chr7	+	37888199	37940003	225	NME8	RefSeq
TC01004954.hg.1	-1.3	7.87	-3.74	2.66E-04	4.36E-03	0.19	TC01004954.	chr1	+	202518232	202523878	30		NONCODE
TC01005192.hg.1	-1.3	8.59	-4.68	6.74E-06	3.37E-04	3.58	TC01005192.	chr1	-	637316	659930	73		Broad
TC12000744.hg.1	-1.3	9.06	-4.55	1.17E-05	4.81E-04	3.07	TC12000744.	chr12	+	96390299	96405267	40		ENSEMBL
TC21000719.hg.1	-1.3	11.1	-3.73	2.79E-04	4.51E-03	0.15	TC21000719.	chr21	+	39645398	39647441	30		NONCODE
TC06001153.hg.1	-1.3	8.7	-4.67	7.13E-06	3.46E-04	3.53	TC06001153.	chr6	+	160514114	160517244	30	LOC729603	RefSeq
TC02000978.hg.1	-1.3	9.17	-5.66	8.60E-08	2.77E-05	7.66	TC02000978.	chr2	+	163175350	163228105	290	GCA	RefSeq
TC07001761.hg.1	-1.3	6.91	-3.47	6.91E-04	8.91E-03	-0.68	TC07001761.	chr7	-	111366164	111846466	809	DOCK4	RefSeq
TC04002929.hg.1	-1.3	8.29	-5.76	5.35E-08	2.18E-05	8.1	TC04002929.	chr4	+	15704573	15739936	179	BST1	RefSeq
TC09002715.hg.1	-1.3	5.33	-4.91	2.54E-06	1.90E-04	4.49	TC09002715.	chr9	-	107606051	107606603	30		NONCODE
TC12001773.hg.1	-1.3	5.3	-5.07	1.27E-06	1.27E-04	5.14	TC12001773.	chr12	-	81226312	81226408	30	MIR617	RefSeq
TC05002656.hg.1	-1.3	8.64	-4.41	2.09E-05	7.05E-04	2.53	TC05002656.	chr5	+	139554736	139623371	30	CYSTM1	NONCODE
TC20000959.hg.1	-1.3	6.31	-5.53	1.59E-07	3.99E-05	7.08	TC20000959.	chr20	-	52491040	52492248	30	SUMO1P1	RefSeq
TC09002184.hg.1	-1.3	8.87	-5.25	5.58E-07	8.17E-05	5.9	TC09002184.	chr9	+	115142189	115234685	100	HSDL2	NONCODE
TC11000751.hg.1	-1.3	7.64	-3.52	5.78E-04	7.77E-03	-0.51	TC11000751.	chr11	+	71846756	71850936	80	FOLR3	RefSeq
TC07001416.hg.1	-1.3	6.64	-4.73	5.43E-06	2.97E-04	3.78	TC07001416.	chr7	-	56469763	56469866	30	RNU6-1052P	ENSEMBL
TC04002243.hg.1	-1.3	7.51	-5.96	1.97E-08	1.43E-05	9.04	TC04002243.	chr4	+	146298061	146299109	30		NONCODE
TC02002670.hg.1	-1.3	8.86	-4.85	3.26E-06	2.23E-04	4.26	TC02002670.	chr2	-	201838441	201936394	355	FAM126B	RefSeq
TC13000644.hg.1	-1.3	7.67	-4.77	4.73E-06	2.75E-04	3.91	TC13000644.	chr13	-	46916137	47012325	314	KIAA0226L	RefSeq
TC05002606.hg.1	-1.3	5.34	-4.44	1.83E-05	6.46E-04	2.66	TC05002606.	chr5	+	126190650	126192242	30		NONCODE
TC07003321.hg.1	-1.3	7.15	-5.21	6.66E-07	9.02E-05	5.74	TC07003321.	chr7	+	37723379	37875470	127	GPR141	RefSeq
TC01004114.hg.1	-1.3	6.15	-4.01	9.88E-05	2.11E-03	1.1	TC01004114.	chr1	+	696291	697369	30		Rinn
TC08000490.hg.1	-1.3	4.68	-4.96	2.03E-06	1.68E-04	4.7	TC08000490.	chr8	+	74991772	74991878	30	RNU6-1197P	ENSEMBL
TC08001979.hg.1	-1.3	5.85	-4.33	2.83E-05	8.67E-04	2.25	TC08001979.	chr8	+	74964575	75012088	30		NONCODE
TC06002121.hg.1	-1.3	11.14	-5.09	1.18E-06	1.22E-04	5.21	TC06002121.	chr6	-	133065009	133084598	180	VNN2	RefSeq
TC01004796.hg.1	-1.3	8.68	-3.93	1.32E-04	2.61E-03	0.84	TC01004796.	chr1	+	161496264	161497073	30	RP11-77M5.1	NONCODE
TC08001265.hg.1	-1.3	5.23	-4.63	8.45E-06	3.86E-04	3.37	TC08001265.	chr8	-	62544239	62544571	30	RN7SKP97	ENSEMBL
TC04000607.hg.1	-1.3	10.85	-4.9	2.67E-06	1.97E-04	4.44	TC04000607.	chr4	+	119555288	119555319	8		GenBank
TC08002047.hg.1	-1.3	6.32	-4.37	2.41E-05	7.76E-04	2.4	TC08002047.	chr8	+	103268620	103270049	30		NONCODE
TC21000717.hg.1	-1.3	10.9	-3.31	1.21E-03	1.36E-02	-1.18	TC21000717.	chr21	+	39601885	39663669	30	KCNJ15	NONCODE
TC19000676.hg.1	-1.3	9.47	-5.2	7.16E-07	9.32E-05	5.67	TC19000676.	chr19	+	47813075	47825327	42	C5AR1	RefSeq
TC12002828.hg.1	-1.3	7.35	-5.52	1.68E-07	4.07E-05	7.03	TC12002828.	chr12	-	32111689	32112260	30		Rinn
TC06003855.hg.1	-1.3	11.73	-4.84	3.38E-06	2.27E-04	4.23	TC06003855.	chr6	-	133065009	133079033	80	VNN2	NONCODE
TC18000318.hg.1	-1.3	9.35	-5.13	9.49E-07	1.10E-04	5.41	TC18000318.	chr18	-	9546789	9614600	257	PPP4R1	RefSeq
TC19002489.hg.1	-1.3	8.86	-5.35	3.65E-07	6.23E-05	6.3	TC19002489.	chr19	-	42125344	42133428	78	CEACAM4	NONCODE
TC01002054.hg.1	-1.3	10.38	-4.58	1.01E-05	4.34E-04	3.2	TC01002054.	chr1	-	141474	149707	60	LOC101928670	ENSEMBL
TC08001408.hg.1	-1.3	8.15	-6.27	4.29E-09	1.18E-05	10.48	TC08001408.	chr8	-	92006024	92053292	150	TMEM55A	RefSeq
TC13000871.hg.1	-1.2	7.91	-3.72	2.88E-04	4.61E-03	0.12	TC13000871.	chr13	-	110406184	110438915	30	IRS2	RefSeq
TC01006214.hg.1	-1.2	6.54	-5.85	3.43E-08	1.85E-05	8.52	TC01006214.	chr1	-	235087871	235105809	183		Rinn

TC09002906.hg.1	-1.2	6.37	-4.73	5.44E-06	2.97E-04	3.78	TC09002906.	chr9	+	117092069	117095536	90	ORM2	RefSeq
TC0X000007.hg.1	-1.2	7.15	-4.26	3.73E-05	1.05E-03	2	TC0X000007.	chrX	+	1412811	1412885	30	MIR3690	RefSeq
TC0Y000007.hg.1	-1.2	7.15	-4.26	3.73E-05	1.05E-03	2	TC0Y000007.	chrY	+	1362811	1362885	30	MIR3690	RefSeq
TC13001241.hg.1	-1.2	7.99	-4.48	1.55E-05	5.77E-04	2.81	TC13001241.	chr13	+	96444953	96445186	30	DJC3	NONCODE
TC01003871.hg.1	-1.2	8.02	-4.27	3.59E-05	1.02E-03	2.03	TC01003871.	chr1	-	223282748	223316624	150	TLR5	RefSeq
TC10002195.hg.1	-1.2	4.41	-4.3	3.27E-05	9.58E-04	2.12	TC10002195.	chr10	+	89739717	89740717	30		Rinn
TC07001921.hg.1	-1.2	6.43	-4.51	1.36E-05	5.27E-04	2.93	TC07001921.	chr7	-	140075363	140075466	30		ENSEMBL
TC09000565.hg.1	-1.2	8.21	-5.39	2.94E-07	5.58E-05	6.51	TC09000565.	chr9	+	115142189	115234690	189	HSDL2	RefSeq
TC13000228.hg.1	-1.2	5.94	-3.23	1.57E-03	1.65E-02	-1.41	TC13000228.	chr13	+	53602830	53626196	110	OLFM4	RefSeq
TC10002048.hg.1	-1.2	6.96	-4.72	5.65E-06	3.03E-04	3.75	TC10002048.	chr10	+	45931684	45934607	30		NONCODE
TC01005185.hg.1	-1.2	10.56	-4.48	1.57E-05	5.83E-04	2.8	TC01005185.	chr1	-	141474	149707	60		Rinn
TC01001047.hg.1	-1.2	4.85	-3.19	1.77E-03	1.81E-02	-1.52	TC01001047.	chr1	+	120906461	120906507	23		UCSC
TC12001651.hg.1	-1.2	4.63	-2.73	7.09E-03	5.17E-02	-2.75	TC12001651.	chr12	-	62511668	62511739	30		ENSEMBL
TC17000813.hg.1	-1.2	8.58	-4.16	5.51E-05	1.38E-03	1.64	TC17000813.	chr17	+	68164814	68176183	50	KCNJ2	RefSeq
TC19000677.hg.1	-1.2	8.71	-5.91	2.54E-08	1.54E-05	8.81	TC19000677.	chr19	+	47835404	47845272	40	C5AR2	RefSeq
TC02002639.hg.1	-1.2	5.55	-4.31	3.14E-05	9.30E-04	2.16	TC02002639.	chr2	-	197079943	197080046	30		ENSEMBL
TC08000487.hg.1	-1.2	6.57	-4.33	2.82E-05	8.64E-04	2.26	TC08000487.	chr8	+	74903564	74941322	60	LY96	RefSeq
TC12002734.hg.1	-1.2	11.43	-4.92	2.48E-06	1.87E-04	4.51	TC12002734.	chr12	-	8074143	8078504	40	SLC2A3	NONCODE
TC04001804.hg.1	-1.2	6.82	-4.76	4.85E-06	2.80E-04	3.89	TC04001804.	chr4	-	185286341	185291024	30	RP11-290F5.1	Havana
TC0X000143.hg.1	-1.2	6.76	-3.07	2.60E-03	2.44E-02	-1.86	TC0X000143.	chrX	+	30716324	30740049	30	RP11-242C19.2	Havana
TC06003169.hg.1	-1.2	8.72	-4.53	1.26E-05	5.03E-04	3	TC06003169.	chr6	+	160514114	160517244	30	LOC729603	NONCODE
TC0X001347.hg.1	-1.2	5.93	-4.05	8.40E-05	1.86E-03	1.25	TC0X001347.	chrX	-	123509753	124097666	328	TENM1	RefSeq
TC04002830.hg.1	-1.2	5.68	-3.93	1.35E-04	2.65E-03	0.81	TC04002830.	chr4	-	174245758	174247299	30		NONCODE
TC10002541.hg.1	-1.2	6.2	-4.45	1.75E-05	6.27E-04	2.7	TC10002541.	chr10	-	31119045	31124561	40		Rinn
TC17001890.hg.1	-1.2	8.93	-5.17	8.18E-07	1.02E-04	5.55	TC17001890.	chr17	-	73937589	73975515	231	ACOX1	RefSeq
TC17000965.hg.1	-1.2	9.49	-4.64	8.12E-06	3.77E-04	3.41	TC17000965.	chr17	-	289769	295731	30	FAM101B	RefSeq
TC16000519.hg.1	-1.2	7.6	-5.91	2.55E-08	1.54E-05	8.8	TC16000519.	chr16	+	66613351	66622178	40	CMTM2	RefSeq
TC17002426.hg.1	-1.2	8.47	-3.94	1.31E-04	2.59E-03	0.84	TC17002426.	chr17	-	289773	292354	36	FAM101B	NONCODE
TC19002306.hg.1	-1.2	8.13	-6.23	5.47E-09	1.19E-05	10.25	TC19002306.	chr19	-	6661264	6663469	26	TNFSF14	NONCODE
TC06000523.hg.1	-1.2	9.07	-4.97	1.93E-06	1.62E-04	4.75	TC06000523.	chr6	+	35995454	36079013	269	MAPK14	RefSeq
TC01001581.hg.1	-1.2	8.66	-5.88	2.93E-08	1.71E-05	8.67	TC01001581.	chr1	+	182758428	182799519	306	NPL	RefSeq
TC08001855.hg.1	-1.2	5.61	-5.58	1.21E-07	3.40E-05	7.34	TC08001855.	chr8	+	28621266	28622725	30		Rinn
TC01001881.hg.1	-1.2	11.35	-4.95	2.17E-06	1.74E-04	4.64	TC01001881.	chr1	+	228160065	228160098	10		GenBank
TC01003854.hg.1	-1.2	11.35	-4.95	2.17E-06	1.74E-04	4.64	TC01003854.	chr1	-	222646010	222646043	10		GenBank
TC6_apd_hap1000078.hg.1	-1.2	9.23	-4.9	2.67E-06	1.97E-04	4.44	TC6_apd_ha	chr6_apd_hap1	-	2007128	2009922	35	FLOT1	RefSeq
TC05000981.hg.1	-1.2	9.27	-4.79	4.24E-06	2.60E-04	4.01	TC05000981.	chr5	+	175085033	175113245	50	HRH2	RefSeq
TC09002219.hg.1	-1.2	9.8	-4.18	5.26E-05	1.34E-03	1.68	TC09002219.	chr9	+	120466460	120479766	40		NONCODE
TC07001534.hg.1	-1.2	7.95	-3.37	9.86E-04	1.17E-02	-1	TC07001534.	chr7	-	75162619	75368283	389	HIP1	RefSeq
TC0Y000089.hg.1	-1.2	11.45	-5	1.71E-06	1.51E-04	4.86	TC0Y000089.	chrY	+	26422647	26422679	9		GenBank
TC0Y000215.hg.1	-1.2	11.45	-5	1.71E-06	1.51E-04	4.86	TC0Y000215.	chrY	-	27539763	27539795	9		GenBank
TC09002578.hg.1	-1.2	7.82	-3.77	2.37E-04	4.03E-03	0.3	TC09002578.	chr9	-	71158457	71161505	47		Rinn
TC10001313.hg.1	-1.2	4.67	-3.52	5.95E-04	7.94E-03	-0.54	TC10001313.	chr10	-	59998418	59998521	30		ENSEMBL
TC12001269.hg.1	-1.2	10.46	-5.2	7.13E-07	9.30E-05	5.68	TC12001269.	chr12	-	14656595	14721283	140	PLBD1	RefSeq
TC01003624.hg.1	-1.2	10.33	-4.6	9.47E-06	4.17E-04	3.27	TC01003624.	chr1	-	184759858	184943682	279	FAM129A	RefSeq
TC11002345.hg.1	-1.2	8.66	-5.1	1.11E-06	1.18E-04	5.26	TC11002345.	chr11	-	118097409	118123065	120	MPZL3	RefSeq
TC01002163.hg.1	-1.2	6.32	-5.46	2.15E-07	4.63E-05	6.8	TC01002163.	chr1	-	7975931	8003225	155	TNFRSF9	RefSeq
TC14001115.hg.1	-1.2	7.02	-5.2	7.13E-07	9.30E-05	5.68	TC14001115.	chr14	-	50575350	50583318	90	VCPKMT	RefSeq
TC01003209.hg.1	-1.2	5.47	-4.42	2.00E-05	6.85E-04	2.57	TC01003209.	chr1	-	150670535	150693364	224	HORMAD1	RefSeq

TC15000949.hg.1	-1.2	8.96	-5.11	1.07E-06	1.16E-04	5.3	TC15000949.	chr15	+	99192200	99507759	270	IGF1R	RefSeq
TC03003384.hg.1	-1.2	7.29	-5.47	2.05E-07	4.49E-05	6.84	TC03003384.	chr3	-	49306030	49314508	110	C3orf62	RefSeq
TC01001676.hg.1	-1.2	6.07	-5.06	1.32E-06	1.29E-04	5.1	TC01001676.	chr1	+	202573396	202574421	30	RP11-569A11.1	Havana
TC11002690.hg.1	-1.2	3.64	-4.63	8.47E-06	3.87E-04	3.37	TC11002690.	chr11	+	59661570	59663119	30		Rinn
TC12002079.hg.1	-1.2	8.7	-4.16	5.57E-05	1.39E-03	1.63	TC12002079.	chr12	-	123199303	123201439	30	HCAR3	RefSeq
TC14000431.hg.1	-1.2	7.36	-3.32	1.14E-03	1.31E-02	-1.13	TC14000431.	chr14	+	71865054	71865124	30	SNORD56B	RefSeq
TC06001714.hg.1	-1.2	9.33	-5.08	1.20E-06	1.23E-04	5.19	TC06001714.	chr6	-	41157552	41168932	70	TREML2	RefSeq
TC11003242.hg.1	-1.2	7.79	-4.62	8.61E-06	3.91E-04	3.35	TC11003242.	chr11	-	72514717	72524260	30		Rinn
TC15001546.hg.1	-1.2	7.77	-5.55	1.43E-07	3.74E-05	7.18	TC15001546.	chr15	-	64199235	64338635	203	DAPK2	RefSeq
TC01004893.hg.1	-1.2	6.18	-5.24	5.83E-07	8.38E-05	5.86	TC01004893.	chr1	+	185339834	185344123	30		Rinn
TC02004639.hg.1	-1.2	3.74	-2.96	3.67E-03	3.16E-02	-2.17	TC02004639.	chr2	-	152236008	152236378	22	TNFAIP6	NONCODE
TC06000119.hg.1	-1.2	8.03	-3.7	3.14E-04	4.94E-03	0.04	TC06000119.	chr6	+	18367060	18367085	2		GenBank
TC05000604.hg.1	-1.2	8.13	-4.39	2.22E-05	7.37E-04	2.47	TC05000604.	chr5	+	126112315	126172712	267	LMNB1	RefSeq
TC05003141.hg.1	-1.2	9.01	-4.21	4.52E-05	1.21E-03	1.82	TC05003141.	chr5	-	90597138	90610346	293		NONCODE
TC09001215.hg.1	-1.2	7.09	-4.68	6.76E-06	3.38E-04	3.58	TC09001215.	chr9	-	77337411	77503010	518	TRPM6	RefSeq
TC01006237.hg.1	-1.2	8.33	-4.69	6.63E-06	3.35E-04	3.6	TC01006237.	chr1	-	243192814	243215554	72		Broad
TC09000601.hg.1	-1.2	9.5	-4.31	3.05E-05	9.13E-04	2.18	TC09000601.	chr9	+	120466453	120479769	120	TLR4	RefSeq
TC17000118.hg.1	-1.2	8.05	-5.51	1.68E-07	4.07E-05	7.03	TC17000118.	chr17	+	7758384	7759417	30	TMEM88	RefSeq
TC17001474.hg.1	-1.2	7.59	-5.05	1.37E-06	1.33E-04	5.06	TC17001474.	chr17	-	39078948	39093836	176	KRT23	RefSeq
TC03000911.hg.1	-1.2	5.85	-3.82	2.04E-04	3.61E-03	0.44	TC03000911.	chr3	+	169775089	169775217	30	RNU4-38P	ENSEMBL
TC09001187.hg.1	-1.2	7.8	-3.74	2.72E-04	4.43E-03	0.17	TC09001187.	chr9	-	71158457	71161505	47	LOC101927015	ENSEMBL
TC04000581.hg.1	-1.2	8.23	-4.29	3.33E-05	9.71E-04	2.1	TC04000581.	chr4	+	113206665	113363776	452	ALPK1	RefSeq
TC02001723.hg.1	-1.2	7.52	-4.51	1.40E-05	5.39E-04	2.9	TC02001723.	chr2	-	32449518	32490923	118	NLRC4	RefSeq
TC05000982.hg.1	-1.2	5.97	-4.63	8.26E-06	3.80E-04	3.39	TC05000982.	chr5	+	175108464	175111558	30	HRH2	RefSeq
TC01005700.hg.1	-1.2	7.15	-5.15	8.95E-07	1.07E-04	5.46	TC01005700.	chr1	-	111390708	111391552	30		Broad
TC12001826.hg.1	-1.2	8.23	-5.52	1.60E-07	4.01E-05	7.07	TC12001826.	chr12	-	94960900	95044338	110	TMCC3	RefSeq
TC08000489.hg.1	-1.2	6.03	-4.59	9.73E-06	4.24E-04	3.24	TC08000489.	chr8	+	74964575	75012088	30	RP11-6I2.3	Havana
TC07003291.hg.1	-1.2	7.96	-5.45	2.27E-07	4.75E-05	6.75	TC07003291.	chr7	+	112063023	112117258	447	IFRD1	RefSeq
TC18000987.hg.1	-1.2	8.17	-6.93	1.49E-10	3.36E-06	13.64	TC18000987.	chr18	-	74072230	74207146	110	ZNF516	NONCODE
TC10001145.hg.1	-1.2	9.18	-4.46	1.70E-05	6.15E-04	2.72	TC10001145.	chr10	-	29833933	29834026	30	MIR604	RefSeq
TC14000941.hg.1	-1.2	7.66	-4.73	5.61E-06	3.02E-04	3.75	TC14000941.	chr14	-	23415437	23451467	132	HAUS4	RefSeq
TC04000827.hg.1	-1.2	6.8	-3.72	2.91E-04	4.65E-03	0.11	TC04000827.	chr4	+	166173732	166173813	30	RNU4-87P	ENSEMBL
TC06001299.hg.1	-1.2	4.74	-4.85	3.30E-06	2.24E-04	4.25	TC06001299.	chr6	-	17878685	17882609	30		ENSEMBL
TC10001312.hg.1	-1.2	8.78	-4.89	2.74E-06	1.99E-04	4.42	TC10001312.	chr10	-	59951278	60027694	70	IPMK	RefSeq
TC19001784.hg.1	-1.2	9.55	-4.3	3.28E-05	9.59E-04	2.11	TC19001784.	chr19	-	52114781	52150151	220	SIGLEC14	RefSeq
TC03003104.hg.1	-1.2	6.38	-3.61	4.27E-04	6.25E-03	-0.24	TC03003104.	chr3	-	134294594	134299804	30		Rinn
TC01003987.hg.1	-1.2	6.15	-6.14	8.24E-09	1.26E-05	9.86	TC01003987.	chr1	-	235093089	235105809	130	RP11-443B7.1	Havana
TC01001400.hg.1	-1.2	10.04	-3.81	2.12E-04	3.71E-03	0.4	TC01001400.	chr1	+	161492935	161493006	30		UCSC
TC10000550.hg.1	-1.2	8.46	-3.73	2.75E-04	4.47E-03	0.16	TC10000550.	chr10	+	79541135	79541163	5		GenBank
TC04000710.hg.1	-1.2	10.19	-6.01	1.58E-08	1.43E-05	9.25	TC04000710.	chr4	+	146296695	146297405	30	RTN3P1	ENSEMBL
TC06002456.hg.1	-1.2	6.52	-5.46	2.21E-07	4.70E-05	6.77	TC06002456.	chr6	+	2993299	3023034	494	MSH5-SAPCD1	NONCODE
TC10000259.hg.1	-1.2	7.88	-5.05	1.36E-06	1.32E-04	5.07	TC10000259.	chr10	+	38742109	38755311	30	RP11-291L22.4	Havana
TC07000401.hg.1	-1.2	7.69	-4.23	4.23E-05	1.15E-03	1.88	TC07000401.	chr7	+	65279659	65279762	30	RNU6-912P	ENSEMBL
TC12002417.hg.1	-1.2	5.77	-4.53	1.26E-05	5.04E-04	3	TC12002417.	chr12	+	65103238	65104293	30		NONCODE
TC0Y000275.hg.1	-1.2	7.95	-4.38	2.36E-05	7.67E-04	2.42	TC0Y000275.	chrY	+	25537324	25538842	30		Broad
TC16001565.hg.1	-1.2	8.19	-2.86	4.89E-03	3.90E-02	-2.43	TC16001565.	chr16	+	53412402	53423133	30		Rinn
TC05002740.hg.1	-1.2	6.86	-3.63	4.05E-04	6.00E-03	-0.19	TC05002740.	chr5	+	172354584	172356104	30		NONCODE
TC06001298.hg.1	-1.2	7.97	-6.07	1.20E-08	1.43E-05	9.51	TC06001298.	chr6	-	17759414	17987854	562	KIF13A	RefSeq

TC04002143.hg.1	-1.2	8.24	-3.55	5.23E-04	7.19E-03	-0.42	TC04002143.	chr4	+	113242860	113243823	30		NONCODE
TC19001500.hg.1	-1.2	9.78	-6.29	3.98E-09	1.17E-05	10.55	TC19001500.	chr19	-	38899698	38916945	240	RASGRP4	RefSeq
TC08002552.hg.1	-1.2	7.54	-4.63	8.32E-06	3.82E-04	3.39	TC08002552.	chr8	-	142208034	142209262	18		Rinn
TC10002529.hg.1	-1.2	7.24	-3.81	2.05E-04	3.62E-03	0.43	TC10002529.	chr10	-	29835750	29837842	30		NONCODE
TC19001787.hg.1	-1.2	9.66	-5.15	8.84E-07	1.06E-04	5.48	TC19001787.	chr19	-	52249023	52255150	40	FPR1	RefSeq
TC12001178.hg.1	-1.2	10.02	-3.28	1.30E-03	1.44E-02	-1.24	TC12001178.	chr12	-	8685901	8693559	90	CLEC4E	RefSeq
TC11000499.hg.1	-1.2	9.57	-5.55	1.41E-07	3.73E-05	7.2	TC11000499.	chr11	+	59480929	59573355	220	STX3	RefSeq
TC10002543.hg.1	-1.2	6.89	-3.24	1.52E-03	1.61E-02	-1.38	TC10002543.	chr10	-	31215453	31215972	21		NONCODE
TC17002572.hg.1	-1.2	8.6	-4.18	5.20E-05	1.33E-03	1.69	TC17002572.	chr17	-	27971812	27972377	30		NONCODE
TC17002303.hg.1	-1.2	4.51	-3.2	1.68E-03	1.74E-02	-1.48	TC17002303.	chr17	+	57862174	57862396	30		NONCODE
TC10000692.hg.1	-1.2	8.04	-5.01	1.61E-06	1.45E-04	4.91	TC10000692.	chr10	+	99079022	99081672	60	FRAT1	RefSeq
TC05001589.hg.1	-1.2	9.56	-4.15	5.86E-05	1.44E-03	1.58	TC05001589.	chr5	-	90598846	90610219	80	LUCAT1	ENSEMBL
TC02003766.hg.1	-1.2	6.77	-5.14	9.06E-07	1.08E-04	5.45	TC02003766.	chr2	+	202038291	202043285	30		Rinn
TC09000314.hg.1	-1.2	5.44	-3.44	7.71E-04	9.70E-03	-0.77	TC09000314.	chr9	+	71150806	71154670	30	TMEM252	GenBank
TC20001381.hg.1	-1.2	7.22	-5.13	9.67E-07	1.10E-04	5.39	TC20001381.	chr20	-	1754011	1760392	40	LOC100289473	NONCODE
TC15002310.hg.1	-1.2	5.74	-3.93	1.35E-04	2.65E-03	0.82	TC15002310.	chr15	+	80253413	80263461	30	BCL2A1	NONCODE
TC02003443.hg.1	-1.2	6.89	-3.69	3.18E-04	4.99E-03	0.03	TC02003443.	chr2	+	102578518	102603782	107		Broad
TC12003306.hg.1	-1.2	4.38	-4.46	1.67E-05	6.10E-04	2.74	TC12003306.	chr12	+	96883353	96948585	170	C12orf55	GenBank
TC12002526.hg.1	-1.2	4.54	-4.55	1.19E-05	4.84E-04	3.06	TC12002526.	chr12	+	96883385	96934745	140	C12orf55	NONCODE
TC01004314.hg.1	-1.2	8.12	-4.88	2.85E-06	2.03E-04	4.38	TC01004314.	chr1	+	36321683	36323276	30		NONCODE
TC02004277.hg.1	-1.2	4.66	-4.14	6.13E-05	1.48E-03	1.54	TC02004277.	chr2	-	64313484	64315380	30		Rinn
TC19001559.hg.1	-1.2	8.66	-5.39	2.99E-07	5.62E-05	6.49	TC19001559.	chr19	-	42125344	42133442	108	CEACAM4	RefSeq
TC22000270.hg.1	-1.2	10.1	-5.95	2.08E-08	1.43E-05	8.99	TC22000270.	chr22	+	37257030	37274059	140	NCF4	RefSeq
TC10001725.hg.1	-1.2	9.4	-5.48	1.95E-07	4.41E-05	6.89	TC10001725.	chr10	-	125767182	125853206	248	CHST15	RefSeq
TC13000336.hg.1	-1.2	8.56	-5.33	3.89E-07	6.44E-05	6.24	TC13000336.	chr13	+	96329393	96447243	140	DJC3	RefSeq
TC01001014.hg.1	-1.2	7.8	-4.72	5.80E-06	3.07E-04	3.72	TC01001014.	chr1	+	116519119	116612675	189	SLC22A15	RefSeq
TC20001463.hg.1	-1.2	8.06	-5.26	5.35E-07	8.01E-05	5.94	TC20001463.	chr20	-	20370273	20372205	30		NONCODE
TC04002523.hg.1	-1.2	9.88	-5.21	6.75E-07	9.09E-05	5.73	TC04002523.	chr4	-	40434720	40631861	60	RBM47	NONCODE
TC10000293.hg.1	-1.2	10.48	-5.34	3.82E-07	6.38E-05	6.26	TC10000293.	chr10	+	45869624	45941567	240	ALOX5	RefSeq
TC10001946.hg.1	-1.2	7.69	-4.57	1.07E-05	4.52E-04	3.15	TC10001946.	chr10	+	26823287	26824417	30		NONCODE
TC05001588.hg.1	-1.2	8.39	-4.32	3.00E-05	9.00E-04	2.2	TC05001588.	chr5	-	90575914	90576959	30	LOC729040	ENSEMBL
TC13001676.hg.1	-1.2	4.59	-2.94	3.87E-03	3.29E-02	-2.22	TC13001676.	chr13	-	109864391	109869109	30		NONCODE
TC0X001567.hg.1	-1.2	9.87	-5.09	1.13E-06	1.20E-04	5.24	TC0X001567.	chrX	+	1387693	1428828	116		NONCODE
TC14001287.hg.1	-1.2	7.6	-4.72	5.81E-06	3.07E-04	3.72	TC14001287.	chr14	-	73738890	73740789	30		ENSEMBL
TC15000796.hg.1	-1.2	8.27	-6.13	8.71E-09	1.26E-05	9.81	TC15000796.	chr15	+	83776159	83806111	120	TM6SF1	RefSeq
TC01000129.hg.1	-1.2	10.02	-5.73	5.97E-08	2.29E-05	8	TC01000129.	chr1	+	10458649	10480233	251	PGD	RefSeq
TC08000402.hg.1	-1.2	8.61	-5.28	4.84E-07	7.50E-05	6.04	TC08000402.	chr8	+	59323823	59364060	140	UBXN2B	RefSeq
TC06003248.hg.1	-1.2	8.76	-4.81	3.97E-06	2.50E-04	4.07	TC06003248.	chr6	-	130803	148170	100		NONCODE
TC11000575.hg.1	-1.2	9.77	-6	1.63E-08	1.43E-05	9.22	TC11000575.	chr11	+	63448918	63527363	140	RTN3	RefSeq
TC08002046.hg.1	-1.2	6.21	-3.83	1.92E-04	3.46E-03	0.49	TC08002046.	chr8	+	103255837	103265561	30	KB-431C1.4	NONCODE
TC10001146.hg.1	-1.2	5.53	-4.29	3.35E-05	9.74E-04	2.09	TC10001146.	chr10	-	29891193	29891275	30	MIR938	RefSeq
TC06001204.hg.1	-1.2	8.11	-4.15	5.90E-05	1.44E-03	1.57	TC06001204.	chr6	+	171044839	171045633	30	XX-C2158C6.1	Havana
TC01003553.hg.1	-1.2	7.29	-4.37	2.39E-05	7.72E-04	2.41	TC01003553.	chr1	-	175986413	175987896	30	RP11-492I21.1	Havana
TC01006200.hg.1	-1.2	6.31	-4.75	5.03E-06	2.85E-04	3.85	TC01006200.	chr1	-	232730260	232734148	40	RP5-855F14.1	NONCODE
TC01003187.hg.1	-1.2	6.77	-4.14	6.07E-05	1.47E-03	1.55	TC01003187.	chr1	-	149754245	149783928	132	HIST2H2BF	RefSeq
TC19002719.hg.1	-1.2	8.32	-5.82	3.95E-08	1.96E-05	8.39	TC19002719.	chr19	-	54818353	54824409	160	LILRA5	RefSeq
TC04002876.hg.1	-1.2	7.17	-4.38	2.31E-05	7.57E-04	2.44	TC04002876.	chr4	-	185286341	185303530	190		NONCODE
TC02004700.hg.1	-1.2	8.16	-3.56	5.18E-04	7.14E-03	-0.41	TC02004700.	chr2	-	175410028	175411063	30		NONCODE

TC16001726.hg.1	-1.2	11.32	-4.09	7.42E-05	1.70E-03	1.36	TC16001726.	chr16	+	90234331	90235598	27		NONCODE
TC04000460.hg.1	-1.2	7.54	-5.78	4.72E-08	2.13E-05	8.22	TC04000460.	chr4	+	84457067	84527028	230	AGPAT9	RefSeq
TC06003341.hg.1	-1.2	5.95	-3.66	3.55E-04	5.43E-03	-0.07	TC06003341.	chr6	-	2835534	2835745	30		NONCODE
TC01004099.hg.1	-1.2	6.63	-4.08	7.58E-05	1.73E-03	1.34	TC01004099.	chr1	+	150309	151388	30	RP11-34P13.9	NONCODE
TC11001678.hg.1	-1.2	3.27	-3.41	8.51E-04	1.04E-02	-0.86	TC11001678.	chr11	-	45734965	45734994	6		GenBank
TC01000127.hg.1	-1.2	7.59	-4.93	2.31E-06	1.78E-04	4.58	TC01000127.	chr1	+	10270764	10441661	680	KIF1B	RefSeq
TC01001624.hg.1	-1.2	11.16	-5.01	1.64E-06	1.47E-04	4.9	TC01001624.	chr1	+	192778169	192781407	110	RGS2	RefSeq
TC06002785.hg.1	-1.2	8.47	-5.22	6.33E-07	8.78E-05	5.79	TC06002785.	chr6	+	41217115	41217327	30	TREML5P	NONCODE
TC01005397.hg.1	-1.2	8.78	-4.25	3.97E-05	1.10E-03	1.94	TC01005397.	chr1	-	33789228	33789621	30		NONCODE
TC14000434.hg.1	-1.2	7.31	-3.72	2.94E-04	4.69E-03	0.1	TC14000434.	chr14	+	71954578	71956420	30	LOC145474	RefSeq
TC17000349.hg.1	-1.2	9.8	-4.74	5.23E-06	2.92E-04	3.82	TC17000349.	chr17	+	28705942	28796730	276	CPD	RefSeq
TC08002531.hg.1	-1.2	7.11	-4.45	1.78E-05	6.36E-04	2.68	TC08002531.	chr8	-	131307601	131308779	30		NONCODE
TC01002763.hg.1	-1.2	7.62	-2.92	4.11E-03	3.44E-02	-2.27	TC01002763.	chr1	-	68564142	68698420	276	WLS	RefSeq
TC06001152.hg.1	-1.2	10.66	-5.19	7.57E-07	9.63E-05	5.62	TC06001152.	chr6	+	160390131	160534539	579	IGF2R	RefSeq
TC12000195.hg.1	-1.2	6.02	-6.14	8.33E-09	1.26E-05	9.85	TC12000195.	chr12	+	14720684	14772689	65	LOC101928290	ENSEMBL
TC04002168.hg.1	-1.2	8.66	-5.08	1.20E-06	1.23E-04	5.19	TC04002168.	chr4	+	120299287	120326770	30		Broad
TC03000231.hg.1	-1.2	8.63	-4.5	1.46E-05	5.54E-04	2.87	TC03000231.	chr3	+	43731605	43775863	217	ABHD5	RefSeq
TC13000870.hg.1	-1.2	4.18	-3.59	4.65E-04	6.61E-03	-0.32	TC13000870.	chr13	-	109816250	109853831	35	MYO16-AS1	ENSEMBL
TC11000813.hg.1	-1.2	6.8	-5.39	2.93E-07	5.58E-05	6.51	TC11000813.	chr11	+	75486949	75487974	30		ENSEMBL
TC05000101.hg.1	-1.2	6.04	-3.62	4.12E-04	6.08E-03	-0.21	TC05000101.	chr5	+	17444119	17484055	40	RP11-321E2.4	Havana
TC20000582.hg.1	-1.2	9.69	-5.37	3.34E-07	5.89E-05	6.39	TC20000582.	chr20	-	3912068	3996229	110	RNF24	RefSeq
TC06003684.hg.1	-1.2	4.63	-3.54	5.42E-04	7.38E-03	-0.45	TC06003684.	chr6	-	53095403	53096831	30		Rinn
TC19002395.hg.1	-1.2	4.78	-3.01	3.07E-03	2.77E-02	-2.01	TC19002395.	chr19	-	21763358	21765406	30		NONCODE
TC17001311.hg.1	-1.2	9.12	-4.94	2.24E-06	1.76E-04	4.61	TC17001311.	chr17	-	27206353	27224715	160	FLOT2	RefSeq
TC02000618.hg.1	-1.2	7.14	-3.59	4.57E-04	6.54E-03	-0.3	TC02000618.	chr2	+	102578518	102603629	30		NONCODE
TC10002018.hg.1	-1.2	8.17	-4.96	2.08E-06	1.71E-04	4.68	TC10002018.	chr10	+	38742109	38764837	60		Broad
TC20000402.hg.1	-1.2	7.63	-3.98	1.11E-04	2.30E-03	0.99	TC20000402.	chr20	+	48909257	48931459	70	LINC01270	RefSeq
TC20000598.hg.1	-1.2	6.88	-5.06	1.30E-06	1.29E-04	5.12	TC20000598.	chr20	-	5488521	5490648	30	LOC101929207	ENSEMBL
TC02001769.hg.1	-1.2	7.37	-4.63	8.44E-06	3.86E-04	3.37	TC02001769.	chr2	-	40339286	40838193	249	SLC8A1	RefSeq
TC10002447.hg.1	-1.2	5.56	-4.76	4.76E-06	2.77E-04	3.91	TC10002447.	chr10	-	6335137	6335689	30		Broad
TC06001715.hg.1	-1.2	6.96	-3.38	9.55E-04	1.14E-02	-0.97	TC06001715.	chr6	-	41176292	41185685	50	TREML3P	RefSeq
TC19000585.hg.1	-1.2	8.7	-5.5	1.80E-07	4.20E-05	6.96	TC19000585.	chr19	+	42300369	42316352	152	CEACAM3	RefSeq
TC01002060.hg.1	-1.2	8.27	-4.45	1.74E-05	6.24E-04	2.7	TC01002060.	chr1	-	536816	659930	93	RP5-857K21.4	Havana
TC04001137.hg.1	-1.2	8.99	-5.42	2.58E-07	5.07E-05	6.63	TC04001137.	chr4	-	40425272	40632892	344	RBM47	RefSeq
TC04001811.hg.1	-1.2	6.47	-4.29	3.38E-05	9.76E-04	2.09	TC04001811.	chr4	-	185764450	185776905	86	LOC731424	RefSeq
TC10000385.hg.1	-1.2	8.73	-5.77	5.05E-08	2.18E-05	8.16	TC10000385.	chr10	+	64893007	64914786	110	NRBF2	RefSeq
TC22001424.hg.1	-1.2	10.08	-4.22	4.35E-05	1.17E-03	1.86	TC22001424.	chr22	+	39348746	39388783	140	APOBEC3A_B	RefSeq
TC0X000066.hg.1	-1.2	8.29	-5.01	1.63E-06	1.46E-04	4.9	TC0X000066.	chrX	+	14891527	14940449	260	MOSPD2	RefSeq
TC08001644.hg.1	-1.2	7.09	-4.33	2.89E-05	8.82E-04	2.23	TC08001644.	chr8	-	131307601	131308779	30	ASAP1-IT1	RefSeq
TC01005805.hg.1	-1.2	8.84	-3.66	3.64E-04	5.54E-03	-0.09	TC01005805.	chr1	-	149397114	149399229	30		NONCODE
TC07000407.hg.1	-1.2	5.26	-4.94	2.21E-06	1.75E-04	4.62	TC07000407.	chr7	+	65860178	65860273	30	RNU6-96P	ENSEMBL
TC09001537.hg.1	-1.2	10.1	-5.9	2.75E-08	1.63E-05	8.73	TC09001537.	chr9	-	123363091	123476765	158	MEGF9	RefSeq
TC12003038.hg.1	-1.2	5.16	-3.38	9.46E-04	1.13E-02	-0.96	TC12003038.	chr12	-	100447869	100448304	30		NONCODE
TC19001242.hg.1	-1.2	10.39	-4.78	4.52E-06	2.69E-04	3.95	TC19001242.	chr19	-	14843205	14889353	282	EMR2	RefSeq
TC08001643.hg.1	-1.2	6.3	-4.64	7.93E-06	3.72E-04	3.43	TC08001643.	chr8	-	131094984	131097014	30	ASAP1-IT2	RefSeq
TC19001353.hg.1	-1.2	4.67	-2.91	4.23E-03	3.51E-02	-2.3	TC19001353.	chr19	-	21763358	21765406	30		UCSC
TC18000581.hg.1	-1.2	8.07	-6.94	1.40E-10	3.36E-06	13.7	TC18000581.	chr18	-	74069637	74207146	90	ZNF516	RefSeq
TC04000775.hg.1	-1.2	9.33	-3.65	3.66E-04	5.56E-03	-0.1	TC04000775.	chr4	+	154605441	154627242	80	TLR2	RefSeq

TC13000100.hg.1	-1.2	9.04	-5.34	3.81E-07	6.38E-05	6.26	TC13000100.	chr13	+	31287615	31338565	90	ALOX5AP	RefSeq
TC13001511.hg.1	-1.2	8.36	-2.82	5.53E-03	4.30E-02	-2.53	TC13001511.	chr13	-	52387483	52419286	30		NONCODE
TC17000117.hg.1	-1.2	9.36	-5.5	1.80E-07	4.20E-05	6.96	TC17000117.	chr17	+	7743222	7758118	250	KDM6B	RefSeq
TC06000120.hg.1	-1.2	8.66	-4.77	4.56E-06	2.70E-04	3.94	TC06000120.	chr6	+	18368779	18469105	160	RNF144B	RefSeq
TC11000182.hg.1	-1.2	7.37	-4.66	7.52E-06	3.60E-04	3.48	TC11000182.	chr11	+	10326227	10328944	151	ADM	RefSeq
TC11002136.hg.1	-1.2	9.21	-5.14	9.38E-07	1.09E-04	5.42	TC11002136.	chr11	-	77926336	78128868	150	GAB2	RefSeq
TC08000793.hg.1	-1.2	9.15	-5.23	6.10E-07	8.55E-05	5.82	TC08000793.	chr8	+	142127377	142205907	548	DENND3	RefSeq
TC01002052.hg.1	-1.2	8.05	-4.21	4.57E-05	1.22E-03	1.81	TC01002052.	chr1	-	89295	133566	131	RP11-34P13.7	Havana
TC15002774.hg.1	-1.2	8.44	-5.98	1.84E-08	1.43E-05	9.11	TC15002774.	chr15	-	55647421	55700708	238	CCPG1	RefSeq
TC20001739.hg.1	-1.2	8.48	-4.51	1.40E-05	5.38E-04	2.91	TC20001739.	chr20	+	62921738	62944485	70	LINC00266-1	RefSeq
TC14001288.hg.1	-1.2	8.98	-5.72	6.41E-08	2.34E-05	7.93	TC14001288.	chr14	-	73741815	73925323	247	NUMB	RefSeq
TC06003117.hg.1	-1.2	6.81	-4.22	4.41E-05	1.19E-03	1.84	TC06003117.	chr6	+	147708804	147711601	30		NONCODE
TC07000774.hg.1	-1.2	9.53	-4.25	3.85E-05	1.07E-03	1.97	TC07000774.	chr7	+	128043764	128043804	17		GenBank
TC08001586.hg.1	-1.2	9.53	-4.25	3.85E-05	1.07E-03	1.97	TC08001586.	chr8	-	124412918	124412958	17		GenBank
TC09000566.hg.1	-1.2	5.88	-4.43	1.93E-05	6.68E-04	2.61	TC09000566.	chr9	+	115178827	115180063	30	HSDL2	GenBank
TC19001826.hg.1	-1.2	9.11	-5.86	3.23E-08	1.80E-05	8.58	TC19001826.	chr19	-	54296855	54327648	152	NLRP12	RefSeq
TC02002279.hg.1	-1.2	9.41	-5.32	4.08E-07	6.62E-05	6.2	TC02002279.	chr2	-	128056245	128146041	230	MAP3K2	RefSeq
TC01003638.hg.1	-1.2	7.81	-4.29	3.33E-05	9.71E-04	2.1	TC01003638.	chr1	-	186640923	186649559	150	PTGS2	RefSeq
TC01002819.hg.1	-1.2	8.79	-5.64	9.48E-08	2.96E-05	7.57	TC01002819.	chr1	-	85018804	85040163	101	CTBS	RefSeq
TC14001116.hg.1	-1.2	10.02	-5.95	2.13E-08	1.44E-05	8.97	TC14001116.	chr14	-	50583846	50698276	274	SOS2	RefSeq
TC13001006.hg.1	-1.2	7.35	-3.61	4.31E-04	6.29E-03	-0.25	TC13001006.	chr13	+	32612375	32614415	30	FRY	NONCODE
TC20001465.hg.1	-1.2	6.36	-4.34	2.75E-05	8.49E-04	2.28	TC20001465.	chr20	-	20650317	20651034	30		NONCODE
TC20000557.hg.1	-1.2	6.14	-4.19	4.89E-05	1.28E-03	1.75	TC20000557.	chr20	-	1784662	1798252	40	RP5-968J1.1	Havana
TC03000637.hg.1	-1.2	7.3	-3.75	2.56E-04	4.24E-03	0.23	TC03000637.	chr3	+	122605360	122611263	30	LOC100129550	RefSeq
TC0X000052.hg.1	-1.2	9.67	-4.81	3.92E-06	2.48E-04	4.09	TC0X000052.	chrX	+	12924739	12941288	50	TLR8	RefSeq
TC10000115.hg.1	-1.2	3.96	-4.31	3.06E-05	9.14E-04	2.18	TC10000115.	chr10	+	15074226	15115851	189	OLAH	RefSeq
TC19001593.hg.1	-1.2	8.72	-5.83	3.73E-08	1.89E-05	8.44	TC19001593.	chr19	-	44150247	44174502	100	PLAUR	RefSeq
TC20000387.hg.1	-1.2	6.75	-3.34	1.07E-03	1.24E-02	-1.07	TC20000387.	chr20	+	47895477	47895565	30	SNORD12C	RefSeq
TC03002233.hg.1	-1.2	7.67	-4.64	7.97E-06	3.72E-04	3.43	TC03002233.	chr3	+	14460408	14461631	30		NONCODE
TC16000137.hg.1	-1.2	8.7	-5.39	2.95E-07	5.58E-05	6.5	TC16000137.	chr16	+	4896666	4932363	240	UBN1	RefSeq
TC15002652.hg.1	-1.2	9.14	-4.97	1.95E-06	1.63E-04	4.74	TC15002652.	chr15	-	80191182	80216096	20	ST20	NONCODE
TC20001650.hg.1	-1.2	6.42	-5.55	1.41E-07	3.73E-05	7.19	TC20001650.	chr20	-	52491042	52492248	30		NONCODE
TC06001154.hg.1	-1.2	7.33	-5.21	6.80E-07	9.09E-05	5.72	TC06001154.	chr6	+	160542805	160579750	154	SLC22A1	RefSeq
TC09002220.hg.1	-1.2	5.16	-4.53	1.29E-05	5.11E-04	2.98	TC09002220.	chr9	+	120521861	120565310	50		Rinn
TC16000375.hg.1	-1.2	10.67	-5.17	8.01E-07	1.01E-04	5.57	TC16000375.	chr16	+	31366509	31394318	370	ITGAX	RefSeq
TC11001649.hg.1	-1.2	6.12	-5.52	1.64E-07	4.06E-05	7.05	TC11001649.	chr11	-	45721045	45721082	14		GenBank
TC04002917.hg.1	-1.2	9.35	-4.56	1.12E-05	4.65E-04	3.11	TC04002917.	chr4	-	38825329	38858438	112	TLR6	RefSeq
TC17001530.hg.1	-1.2	7.66	-3.76	2.48E-04	4.15E-03	0.25	TC17001530.	chr17	-	40423352	40424701	30	AC003104.1	Havana
TC19001977.hg.1	-1.2	7.68	-4.84	3.51E-06	2.33E-04	4.19	TC19001977.	chr19	+	10396161	10396374	30	ICAM1	NONCODE
TC19001825.hg.1	-1.2	6.35	-6.65	6.32E-10	5.34E-06	12.28	TC19001825.	chr19	-	54290931	54290996	30	MIR371B	RefSeq
TC01000352.hg.1	-1.2	9.8	-5.6	1.12E-07	3.24E-05	7.41	TC01000352.	chr1	+	27153201	27183406	160	ZDHHC18	RefSeq
TC10000216.hg.1	-1.2	6.68	-5.67	8.22E-08	2.67E-05	7.7	TC10000216.	chr10	+	30981203	31005942	120	SVILP1	ENSEMBL
TC11000753.hg.1	-1.2	7.65	-3.75	2.64E-04	4.34E-03	0.2	TC11000753.	chr11	+	71883483	71889086	40		ENSEMBL
TC06003642.hg.1	-1.2	6.57	-3.03	2.90E-03	2.65E-02	-1.96	TC06003642.	chr6	-	41176294	41185685	50		NONCODE
TC0X002184.hg.1	-1.2	6.22	-3.34	1.09E-03	1.26E-02	-1.09	TC0X002184.	chrX	-	108975214	108975708	30		NONCODE
TC09000857.hg.1	-1.2	11.24	-4.75	5.09E-06	2.87E-04	3.84	TC09000857.	chr9	+	141106637	141149300	147	FAM157B	RefSeq
TC06001326.hg.1	-1.2	5.79	-6.43	1.98E-09	9.53E-06	11.21	TC06001326.	chr6	-	24544331	24646383	310	KIAA0319	RefSeq
TC0Y000339.hg.1	-1.2	7.62	-4.37	2.39E-05	7.72E-04	2.41	TC0Y000339.	chrY	-	27524447	27540866	30		Broad

TC22000937.hg.1	-1.2	9.54	-4.96	2.02E-06	1.67E-04	4.7	TC22000937.	chr22	+	17593918	17596583	60		NONCODE
TC0X000206.hg.1	-1.2	8.48	-4.35	2.66E-05	8.31E-04	2.31	TC0X000206.	chrX	+	43137549	43137585	13		GenBank
TC03002524.hg.1	-1.2	9.34	-5.61	1.06E-07	3.18E-05	7.46	TC03002524.	chr3	+	127391781	127399769	114	ABTB1	NONCODE
TC01001315.hg.1	-1.2	8.23	-5.73	5.98E-08	2.29E-05	8	TC01001315.	chr1	+	156163730	156182587	192	SLC25A44	RefSeq
TC20001279.hg.1	-1.2	7.1	-4.1	6.95E-05	1.62E-03	1.42	TC20001279.	chr20	+	48909257	48932000	112		Rinn
TC07001901.hg.1	-1.2	9.53	-4.17	5.32E-05	1.35E-03	1.67	TC07001901.	chr7	-	138125547	138125585	15		GenBank
TC20000685.hg.1	-1.2	8.55	-5.02	1.60E-06	1.44E-04	4.92	TC20000685.	chr20	-	20370196	20693266	542	RALGAPA2	RefSeq
TC20001464.hg.1	-1.2	7.79	-3.88	1.63E-04	3.06E-03	0.64	TC20001464.	chr20	-	20389471	20390912	30	RALGAPA2	NONCODE
TC01002990.hg.1	-1.2	7.73	-5.52	1.64E-07	4.06E-05	7.05	TC01002990.	chr1	-	112264686	112298446	73	FAM212B	RefSeq
TC19000228.hg.1	-1.2	8.91	-5.3	4.45E-07	7.00E-05	6.12	TC19000228.	chr19	+	12902310	12904125	30	JUNB	RefSeq
TC20001488.hg.1	-1.2	8.08	-2.99	3.31E-03	2.93E-02	-2.08	TC20001488.	chr20	-	23046042	23052105	30		Rinn
TC20000527.hg.1	-1.2	7.4	-5.22	6.34E-07	8.78E-05	5.79	TC20000527.	chr20	+	62507484	62512243	30	LOC100505815	RefSeq
TC07001738.hg.1	-1.2	10.46	-4.97	1.92E-06	1.62E-04	4.75	TC07001738.	chr7	-	105888731	105926772	329	MPT	RefSeq
TC02003275.hg.1	-1.2	6.11	-3.1	2.38E-03	2.28E-02	-1.79	TC02003275.	chr2	+	64566991	64581542	30	AC008074.4	NONCODE
TC08002026.hg.1	-1.2	9.53	-4.6	9.51E-06	4.18E-04	3.26	TC08002026.	chr8	+	97797356	97847419	30	CPQ	NONCODE
TC08002487.hg.1	-1.2	7.27	-3.55	5.20E-04	7.16E-03	-0.42	TC08002487.	chr8	-	119097734	119100498	30		NONCODE
TC01003774.hg.1	-1.2	4.82	-3.9	1.48E-04	2.84E-03	0.73	TC01003774.	chr1	-	207725270	207726324	30	RP11-78B10.2	Havana
TC10000360.hg.1	-1.2	9.34	-3.94	1.30E-04	2.58E-03	0.85	TC10000360.	chr10	+	60094735	60130513	111	UBE2D1	RefSeq
TC0X000356.hg.1	-1.2	10.04	-4.54	1.22E-05	4.92E-04	3.03	TC0X000356.	chrX	+	65235303	65240264	50	MIR223	RefSeq
TC07002619.hg.1	-1.2	8.9	-3.7	3.09E-04	4.87E-03	0.06	TC07002619.	chr7	+	139639478	139646962	30		NONCODE
TC02001749.hg.1	-1.2	7.05	-2.87	4.78E-03	3.84E-02	-2.41	TC02001749.	chr2	-	38294116	38297840	30	CYP1B1	Havana
TC08001690.hg.1	-1.2	8.37	-4.98	1.91E-06	1.61E-04	4.76	TC08001690.	chr8	-	142217265	142318404	220	SLC45A4	RefSeq
TC20001675.hg.1	-1.2	7.15	-4.92	2.39E-06	1.83E-04	4.55	TC20001675.	chr20	-	56807833	56884495	160	PPP4R1L	NONCODE
TC05000398.hg.1	-1.2	7.42	-4.87	3.08E-06	2.13E-04	4.31	TC05000398.	chr5	+	79703832	79775498	380	ZFYVE16	RefSeq
TC16001724.hg.1	-1.2	11.45	-4.7	6.26E-06	3.24E-04	3.65	TC16001724.	chr16	+	90168702	90204399	90		Broad
TC11002623.hg.1	-1.2	5.74	-2.87	4.73E-03	3.81E-02	-2.4	TC11002623.	chr11	+	32875249	32879666	30	PRRG4	NONCODE
TC6_mcf_hap5000121.hg.1	-1.2	9.04	-5.22	6.44E-07	8.81E-05	5.77	TC6_mcf_ha	chr6_mcf_hap5	-	2077369	2091199	167	FLOT1	RefSeq
TC16002057.hg.1	-1.2	6.24	-3.52	5.90E-04	7.89E-03	-0.53	TC16002057.	chr16	+	72088508	72094955	98	HP	RefSeq
TC06001941.hg.1	-1.2	7.31	-6.41	2.19E-09	9.87E-06	11.11	TC06001941.	chr6	-	90074335	90121995	130	RRAGD	RefSeq
TC07002428.hg.1	-1.2	5.93	-4.17	5.27E-05	1.34E-03	1.68	TC07002428.	chr7	+	77313168	77314645	30		NONCODE
TC19000470.hg.1	-1.2	9.61	-3.57	4.96E-04	6.93E-03	-0.38	TC19000470.	chr19	+	35939203	35942669	40	FFAR2	RefSeq
TC02003718.hg.1	-1.2	6.8	-3.65	3.78E-04	5.70E-03	-0.13	TC02003718.	chr2	+	179419288	179423693	30		NONCODE
TC01002322.hg.1	-1.2	9.3	-5.56	1.35E-07	3.68E-05	7.23	TC01002322.	chr1	-	21543740	21672034	376	ECE1	RefSeq
TC15002546.hg.1	-1.2	6.61	-6.54	1.10E-09	6.72E-06	11.76	TC15002546.	chr15	-	55647421	55790782	160	DYX1C1-CCPG1	NONCODE
TC05002297.hg.1	-1.2	6.68	-3.82	2.02E-04	3.58E-03	0.44	TC05002297.	chr5	+	17444119	17485937	60		Rinn
TC13001007.hg.1	-1.2	6.85	-4.06	8.12E-05	1.81E-03	1.28	TC13001007.	chr13	+	32632895	32633647	60		NONCODE
TC20000556.hg.1	-1.2	7.27	-4.71	6.07E-06	3.17E-04	3.68	TC20000556.	chr20	-	1754011	1760392	40	LOC100289473	RefSeq
TC10002943.hg.1	-1.2	8.59	-4.39	2.24E-05	7.39E-04	2.47	TC10002943.	chr10	+	97454774	97637023	405	ENTPD1	RefSeq
TC10002659.hg.1	-1.2	6.52	-3.86	1.75E-04	3.24E-03	0.57	TC10002659.	chr10	-	59960595	59961847	30		NONCODE
TC10000541.hg.1	-1.2	8.74	-5.67	8.17E-08	2.66E-05	7.71	TC10000541.	chr10	+	79540542	79540571	6		GenBank
TC04001740.hg.1	-1.2	6.41	-3.71	3.04E-04	4.80E-03	0.07	TC04001740.	chr4	-	174243357	174250845	30	RP11-798M19.3	Havana
TC19001180.hg.1	-1.2	9.69	-3.67	3.45E-04	5.32E-03	-0.05	TC19001180.	chr19	-	11411545	11431999	26		GenBank
TC16000717.hg.1	-1.2	10.89	-4.61	9.00E-06	4.01E-04	3.31	TC16000717.	chr16	+	90237092	90238407	29		GenBank
TC10002542.hg.1	-1.2	8.09	-3.78	2.36E-04	4.02E-03	0.3	TC10002542.	chr10	-	31133565	31320866	70	ZNF438	NONCODE
TC11001192.hg.1	-1.2	7.47	-5.48	2.00E-07	4.45E-05	6.86	TC11001192.	chr11	+	129685714	129729898	114	TMEM45B	RefSeq
TC08000591.hg.1	-1.2	8.42	-5.14	9.34E-07	1.09E-04	5.42	TC08000591.	chr8	+	97657455	98161882	195	CPQ	RefSeq
TC10001444.hg.1	-1.2	10.43	-5.41	2.70E-07	5.24E-05	6.59	TC10001444.	chr10	-	79541030	79541072	19		GenBank
TC10001144.hg.1	-1.2	8.51	-5.37	3.29E-07	5.89E-05	6.4	TC10001144.	chr10	-	29746267	30025864	635	SVIL	RefSeq

TC16001663.hg.1	-1.2	7.07	-4.3	3.22E-05	9.46E-04	2.13	TC16001663.	chr16	+	81994217	81996288	30	PLCG2	NONCODE
TC0X000188.hg.1	-1.2	9.43	-3.97	1.14E-04	2.34E-03	0.97	TC0X000188.	chrX	+	40219848	40219877	6		GenBank
TC08001550.hg.1	-1.2	7.27	-3.54	5.50E-04	7.46E-03	-0.47	TC08001550.	chr8	-	119097733	119100498	30		GenBank
TC0X000956.hg.1	-1.2	6.53	-3.95	1.27E-04	2.54E-03	0.87	TC0X000956.	chrX	-	38167365	38167494	30		ENSEMBL
TC12001157.hg.1	-1.2	8.16	-5.53	1.58E-07	3.97E-05	7.09	TC12001157.	chr12	-	7246303	7261869	162	C1RL	RefSeq
TC17001943.hg.1	-1.2	6.97	-5.99	1.74E-08	1.43E-05	9.16	TC17001943.	chr17	-	79099683	79099749	30	MIR338	RefSeq
TC11000544.hg.1	-1.2	8.08	-5.75	5.56E-08	2.25E-05	8.07	TC11000544.	chr11	+	61717293	61732987	266	BEST1	RefSeq
TC02003717.hg.1	-1.2	6.94	-3.66	3.54E-04	5.43E-03	-0.07	TC02003717.	chr2	+	179413611	179415408	30	TTN	NONCODE
TC0Y000006.hg.1	-1.2	9.51	-5.09	1.18E-06	1.21E-04	5.21	TC0Y000006.	chrY	+	1337693	1379274	186	CSF2RA	RefSeq
TC04000612.hg.1	-1.2	6.41	-3.72	2.94E-04	4.69E-03	0.1	TC04000612.	chr4	+	120288717	120288801	30	RNU4-33P	ENSEMBL
TC11000310.hg.1	-1.2	7.95	-3.35	1.04E-03	1.22E-02	-1.05	TC11000310.	chr11	+	32851481	32879669	80	PRRG4	RefSeq
TC01003550.hg.1	-1.2	5.11	-3.88	1.60E-04	3.02E-03	0.66	TC01003550.	chr1	-	175873898	175889649	30	RP11-318C24.2	Havana
TC13000110.hg.1	-1.2	6.4	-4.51	1.40E-05	5.39E-04	2.9	TC13000110.	chr13	+	32872291	32872849	30	RP11-37E23.5	NONCODE
TC01005732.hg.1	-1.2	8	-5.62	1.03E-07	3.13E-05	7.48	TC01005732.	chr1	-	117057156	117113715	70	CD58	NONCODE
TC16000812.hg.1	-1.2	9.42	-5.37	3.23E-07	5.85E-05	6.42	TC16000812.	chr16	-	3292027	3306627	158	MEFV	RefSeq
TC03002165.hg.1	-1.2	8.53	-4.66	7.53E-06	3.60E-04	3.48	TC03002165.	chr3	-	196433148	196439164	50	CEP19	RefSeq
TC0X000006.hg.1	-1.2	9.22	-5.14	9.16E-07	1.08E-04	5.44	TC0X000006.	chrX	+	1387693	1429274	288	CSF2RA	RefSeq
TC08000488.hg.1	-1.2	6.34	-4.58	1.05E-05	4.45E-04	3.17	TC08000488.	chr8	+	74960912	74961019	30	RNU6-1300P	ENSEMBL
TC10000182.hg.1	-1.2	9.13	-4.32	2.92E-05	8.86E-04	2.22	TC10000182.	chr10	+	26798518	26798633	30	R5SP307	ENSEMBL
TC16000714.hg.1	-1.2	11.43	-4.53	1.26E-05	5.03E-04	3	TC16000714.	chr16	+	90168702	90204399	90		UCSC
TC02002145.hg.1	-1.2	10.06	-4.57	1.09E-05	4.58E-04	3.13	TC02002145.	chr2	-	101887681	101925178	173	RNF149	RefSeq
TC20001365.hg.1	-1.2	8.59	-4.87	3.07E-06	2.13E-04	4.31	TC20001365.	chr20	+	62921738	62944485	60		Broad
TC01005955.hg.1	-1.2	5.13	-4.02	9.53E-05	2.05E-03	1.13	TC01005955.	chr1	-	175873898	175889649	30		Rinn
TC02004970.hg.1	-1.2	11.17	-5.53	1.54E-07	3.92E-05	7.11	TC02004970.	chr2	+	70142173	70170077	213	MXD1	RefSeq
TC15000951.hg.1	-1.2	5.9	-4.49	1.47E-05	5.58E-04	2.86	TC15000951.	chr15	+	99441845	99443609	30	IGF1R	GenBank
TC01000462.hg.1	-1.2	9.85	-5.55	1.39E-07	3.72E-05	7.2	TC01000462.	chr1	+	36273773	36323491	260	AGO4	RefSeq
TC19001102.hg.1	-1.2	8.54	-5.77	5.00E-08	2.18E-05	8.17	TC19001102.	chr19	-	6663148	6670599	60	TNFSF14	RefSeq
TC20000391.hg.1	-1.2	8.84	-5.11	1.05E-06	1.15E-04	5.32	TC20000391.	chr20	+	48429250	48508779	240	SLC9A8	RefSeq
TC09002052.hg.1	-1.2	5.78	-3.98	1.10E-04	2.28E-03	1.01	TC09002052.	chr9	+	88358355	88401763	80		Broad
TC15002747.hg.1	-1.2	5.3	-3.76	2.53E-04	4.21E-03	0.24	TC15002747.	chr15	-	101709636	101711509	30		Rinn
TC10002194.hg.1	-1.2	7.45	-3.57	5.01E-04	6.98E-03	-0.38	TC10002194.	chr10	+	89706925	89708694	30	PTEN	NONCODE
TC03000087.hg.1	-1.2	9.63	-5.13	9.73E-07	1.10E-04	5.39	TC03000087.	chr3	+	14444076	14530857	279	SLC6A6	RefSeq
TC15002712.hg.1	-1.2	4.65	-3.98	1.11E-04	2.29E-03	0.99	TC15002712.	chr15	-	93323486	93324722	30		NONCODE
TC02002767.hg.1	-1.2	10.87	-4.68	6.94E-06	3.42E-04	3.56	TC02002767.	chr2	-	219027568	219031725	99	CXCR1	RefSeq
TC01005312.hg.1	-1.2	6.71	-4.63	8.55E-06	3.90E-04	3.36	TC01005312.	chr1	-	21602543	21604868	30		NONCODE
TC01001276.hg.1	-1.2	9.47	-4.67	9.71E-06	4.63E-04	3.27	TC01001276.	chr1	+	154377669	154441926	227	IL6R	RefSeq
TC19002293.hg.1	1.2	10.64	5.1	1.70E-06	1.58E-04	4.9	TC19002293.	chr19	-	3053866	3061276	60	AES	NONCODE
TC08000529.hg.1	1.2	4.94	5.52	2.90E-07	6.64E-05	6.56	TC08000529.	chr8	+	86089460	86129387	232	E2F5	RefSeq
TC12001544.hg.1	1.2	8.52	6.27	1.00E-08	1.94E-05	9.72	TC12001544.	chr12	-	53585107	53601091	235	ITGB7	RefSeq
TC6_apd_hap1000118.hg.1	1.2	9.08	4.74	7.50E-06	3.88E-04	3.51	TC6_apd_ha	chr6_apd_hap1	-	4203376	4207888	125	HLA-DMA	RefSeq
TC01005671.hg.1	1.2	9.05	4.65	7.69E-06	3.64E-04	3.46	TC01005671.	chr1	-	102251817	102252854	30		Broad
TC11002439.hg.1	1.2	9.3	5.81	4.12E-08	2.00E-05	8.35	TC11002439.	chr11	-	128328656	128457453	190	ETS1	RefSeq
TC15000045.hg.1	1.2	6.76	4.06	8.36E-05	1.85E-03	1.25	TC15000045.	chr15	+	25230007	25233379	50	SNORD108	RefSeq
TC01004246.hg.1	1.2	7.73	3.91	1.45E-04	2.81E-03	0.74	TC01004246.	chr1	+	24865207	24865735	30		NONCODE
TC0X000427.hg.1	1.2	7.4	3.75	2.63E-04	4.33E-03	0.2	TC0X000427.	chrX	+	78200829	78217451	89	P2RY10	RefSeq
TC06001577.hg.1	1.2	7.18	5.99	1.71E-08	1.43E-05	9.18	TC06001577.	chr6	-	32971955	32977389	207	HLA-DOA	RefSeq
TC01002663.hg.1	1.2	6.23	4.43	1.95E-05	6.75E-04	2.6	TC01002663.	chr1	-	53458868	53459251	30		ENSEMBL
TC0X000428.hg.1	1.2	8.22	3.41	8.53E-04	1.05E-02	-0.87	TC0X000428.	chrX	+	78426469	78427726	30	GPR174	RefSeq

TC02002490.hg.1	1.2	4.4	3.63	3.99E-04	5.94E-03	-0.18	TC02002490.	chr2	-	165752696	165812035	287	SLC38A11	RefSeq
TC01003767.hg.1	1.2	8.92	4.72	5.71E-06	3.04E-04	3.74	TC01003767.	chr1	-	207076631	207095378	180	FAIM3	RefSeq
TC17001637.hg.1	1.2	7.77	5.15	8.71E-07	1.06E-04	5.49	TC17001637.	chr17	-	46210802	46507594	146	SKAP1	RefSeq
TC14002189.hg.1	1.2	8.09	3.11	2.28E-03	2.21E-02	-1.75	TC14002189.	chr14	-	106068003	107218968	1076	IGHG1	NONCODE
TC02004724.hg.1	1.2	6.91	4.48	1.53E-05	5.72E-04	2.82	TC02004724.	chr2	-	179537189	179544381	60	TTN	NONCODE
TC14001478.hg.1	1.2	7.28	3.88	1.64E-04	3.08E-03	0.63	TC14001478.	chr14	-	96176304	96180533	80	TCL1A	RefSeq
TC14001441.hg.1	1.2	9.02	4.71	5.92E-06	3.11E-04	3.7	TC14001441.	chr14	-	92246095	92333880	218	TC2N	RefSeq
TC06001094.hg.1	1.2	5.66	5.44	2.35E-07	4.86E-05	6.71	TC06001094.	chr6	+	150920999	151164799	315	PLEKHG1	RefSeq
TC02002478.hg.1	1.2	7.06	4.62	8.58E-06	3.90E-04	3.36	TC02002478.	chr2	-	162848751	162931052	410	DPP4	RefSeq
TC15000049.hg.1	1.2	5.71	2.92	4.08E-03	3.41E-02	-2.27	TC15000049.	chr15	+	25299356	25299452	30	SNORD116-2	RefSeq
TC02000038.hg.1	1.2	6.22	2.94	3.88E-03	3.29E-02	-2.22	TC02000038.	chr2	+	7865932	7870836	30	LOC101929531	ENSEMBL
TC06004125.hg.1	1.2	6.96	3.27	1.35E-03	1.48E-02	-1.28	TC06004125.	chr6	-	32780540	32784825	148	HLA-DOB	RefSeq
TC01004517.hg.1	1.2	4.58	2.81	5.61E-03	4.35E-02	-2.55	TC01004517.	chr1	+	89647181	89647394	30		NONCODE
TC15000436.hg.1	1.2	7.1	4.29	3.35E-05	9.74E-04	2.1	TC15000436.	chr15	+	57592563	57599967	72	LINC00926	RefSeq
TC04002090.hg.1	1.2	8.6	4.68	6.70E-06	3.37E-04	3.59	TC04002090.	chr4	+	84009930	84010394	33		Rinn
TC10001863.hg.1	1.2	6.24	3.01	3.11E-03	2.79E-02	-2.03	TC10001863.	chr10	+	6660597	6667308	66		Rinn
TC03000563.hg.1	1.2	8.84	4.68	6.67E-06	3.36E-04	3.59	TC03000563.	chr3	+	111260926	111384597	248	CD96	RefSeq
TC0X000666.hg.1	1.2	7.32	3.68	3.31E-04	5.14E-03	-0.01	TC0X000666.	chrX	+	135730336	135742549	70	CD40LG	RefSeq
TC14000101.hg.1	1.2	6.69	3.74	2.66E-04	4.36E-03	0.19	TC14000101.	chr14	+	22336980	22733726	30	TRAV13-1	ENSEMBL
TC12001578.hg.1	1.2	8.69	4.66	7.35E-06	3.55E-04	3.5	TC12001578.	chr12	-	55341802	55378456	210	TESPA1	RefSeq
TC16000386.hg.1	1.2	7.18	2.91	4.20E-03	3.49E-02	-2.29	TC16000386.	chr16	+	32063311	32063601	30		ENSEMBL
TC08002364.hg.1	1.2	7.79	2.71	7.63E-03	5.47E-02	-2.82	TC08002364.	chr8	-	67476954	67525175	160	MYBL1	NONCODE
TC04001092.hg.1	1.2	7.51	6	1.65E-08	1.43E-05	9.21	TC04001092.	chr4	-	25749049	25865382	437	SEL1L3	RefSeq
TC6_mcf_hap5000170.hg.1	1.2	7.72	4.3	3.24E-05	9.51E-04	2.13	TC6_mcf_ha	chr6_mcf_hap5	-	3961419	3970467	100	HLA-DQB1	GenBank
TC15000064.hg.1	1.2	8.73	3.23	1.55E-03	1.63E-02	-1.4	TC15000064.	chr15	+	25330531	25330624	30	SNORD116-18	RefSeq
TC14001586.hg.1	1.2	6.13	3.43	7.99E-04	9.96E-03	-0.81	TC14001586.	chr14	+	22180592	22181058	17		NONCODE
TC15000051.hg.1	1.2	5.31	3.53	5.65E-04	7.63E-03	-0.49	TC15000051.	chr15	+	25307479	25307575	30	SNORD116-5	RefSeq
TC15000053.hg.1	1.2	5.31	3.53	5.65E-04	7.63E-03	-0.49	TC15000053.	chr15	+	25312934	25313030	30	SNORD116-5	RefSeq
TC15002563.hg.1	1.2	7.39	3.58	4.81E-04	6.76E-03	-0.35	TC15002563.	chr15	-	60780483	60783123	30	RORA	NONCODE
TC05000865.hg.1	1.2	8.39	4.84	3.49E-06	2.32E-04	4.2	TC05000865.	chr5	+	156569944	156682201	323	ITK	RefSeq
TC02005047.hg.1	1.2	6.41	4.48	1.57E-05	5.83E-04	2.8	TC02005047.	chr2	-	179390716	179672150	3708	TTN	RefSeq
TC11001061.hg.1	1.2	9.36	4.38	2.33E-05	7.61E-04	2.43	TC11001061.	chr11	+	118215059	118225876	126	CD3G	RefSeq
TC03001052.hg.1	1.2	6.81	4.87	3.09E-06	2.13E-04	4.31	TC03001052.	chr3	+	191046866	191116459	160	CCDC50	RefSeq
TC15000052.hg.1	1.2	5.68	3.15	2.01E-03	2.00E-02	-1.64	TC15000052.	chr15	+	25310172	25310269	30	SNORD116-2	RefSeq
TC01001758.hg.1	1.2	6.93	5.76	5.31E-08	2.18E-05	8.11	TC01001758.	chr1	+	211499957	211548288	226	TRAF5	RefSeq
TC12001426.hg.1	1.2	9.12	6.02	1.52E-08	1.43E-05	9.28	TC12001426.	chr12	-	46576841	46663208	267	SLC38A1	RefSeq
TC01005698.hg.1	1.2	6.9	3.95	1.24E-04	2.50E-03	0.89	TC01005698.	chr1	-	111196209	111197947	30		NONCODE
TC14000126.hg.1	1.2	8.35	3.29	1.29E-03	1.43E-02	-1.24	TC14000126.	chr14	+	22999278	22999329	28	TRAJ14	ENSEMBL
TC18000554.hg.1	1.2	8.62	5.24	5.86E-07	8.38E-05	5.86	TC18000554.	chr18	-	60790579	60987361	109	BCL2	RefSeq
TC15001217.hg.1	1.2	8.63	5.16	8.63E-07	1.05E-04	5.5	TC15001217.	chr15	-	38780302	38857776	230	RASGRP1	RefSeq
TC15002091.hg.1	1.2	6.82	4.46	1.72E-05	6.19E-04	2.71	TC15002091.	chr15	+	25230007	25233381	60	PWAR5	NONCODE
TC6_dbb_hap3000180.hg.1	1.2	11.25	3.99	1.09E-04	2.27E-03	1.01	TC6_dbb_hap	chr6_dbb_hap3	-	3794488	3794590	8	HLA-DRB1	GenBank
TC14001850.hg.1	1.2	8.06	5.71	6.56E-08	2.36E-05	7.91	TC14001850.	chr14	+	100610216	100610573	30		NONCODE
TC02001083.hg.1	1.2	9.01	5.24	5.84E-07	8.38E-05	5.86	TC02001083.	chr2	+	182321619	182402468	480	ITGA4	RefSeq
TC6_dbb_hap3000084.hg.1	1.2	7.27	3.74	2.70E-04	4.41E-03	0.18	TC6_dbb_hap	chr6_dbb_hap3	+	3877688	3900041	185	HLA-DQA1	GenBank
TC6_mann_hap4000075.hg.1	1.2	7.27	3.74	2.70E-04	4.41E-03	0.18	TC6_mann_h	chr6_mann_hap4	+	4052121	4074486	185	HLA-DQA1	GenBank
TC01001974.hg.1	1.2	5.6	4.42	2.01E-05	6.89E-04	2.57	TC01001974.	chr1	+	241695434	241758949	280	KMO	RefSeq
TC05002628.hg.1	1.2	9.08	4.09	7.38E-05	1.69E-03	1.37	TC05002628.	chr5	+	133451350	133483920	80	TCF7	NONCODE

TC14002280.hg.1	1.2	7.5	2.8	5.90E-03	4.52E-02	-2.59	TC14002280.	chr14	-	107169931	107170428	22	IGHV1-69	ENSEMBL
TC16001073.hg.1	1.2	7.06	2.89	4.44E-03	3.64E-02	-2.34	TC16001073.	chr16	-	33740806	33741090	30		ENSEMBL
TC15000061.hg.1	1.2	9.23	2.96	3.62E-03	3.13E-02	-2.16	TC15000061.	chr15	+	25326433	25326526	30	SNORD116-15	RefSeq
TC11003270.hg.1	1.2	5.71	4.23	4.31E-05	1.17E-03	1.86	TC11003270.	chr11	-	82686479	82688475	30	RAB30	NONCODE
TC14002284.hg.1	1.2	6.98	2.98	3.36E-03	2.96E-02	-2.09	TC14002284.	chr14	-	107218676	107219365	30	IGHV3-74	ENSEMBL
TC11000956.hg.1	1.2	7.97	4.44	1.80E-05	6.40E-04	2.67	TC11000956.	chr11	+	102188181	102210135	160	BIRC3	RefSeq
TC01001339.hg.1	1.2	6.33	5	1.74E-06	1.53E-04	4.84	TC01001339.	chr1	+	158259563	158264564	120	CD1C	RefSeq
TC01001022.hg.1	1.2	8.36	4.39	2.25E-05	7.41E-04	2.47	TC01001022.	chr1	+	117297007	117311851	90	CD2	RefSeq
TC15000060.hg.1	1.2	6.79	2.83	5.28E-03	4.14E-02	-2.49	TC15000060.	chr15	+	25325288	25325381	30	SNORD116-14	RefSeq
TC08000023.hg.1	1.2	5.97	4.34	2.75E-05	8.49E-04	2.28	TC08000023.	chr8	+	6602685	6602765	30	MIR4659A	RefSeq
TC02002827.hg.1	1.2	9.22	5.27	5.25E-07	7.99E-05	5.96	TC02002827.	chr2	-	225629807	225907330	718	DOCK10	RefSeq
TC16001860.hg.1	1.2	7.99	2.94	3.83E-03	3.27E-02	-2.21	TC16001860.	chr16	-	33647308	33661699	30		Broad
TC15000050.hg.1	1.2	6.97	3.63	3.96E-04	5.90E-03	-0.17	TC15000050.	chr15	+	25302006	25302102	30	SNORD116-3	RefSeq
TC15000055.hg.1	1.2	6.97	3.63	3.96E-04	5.90E-03	-0.17	TC15000055.	chr15	+	25318253	25318349	30	SNORD116-3	RefSeq
TC01003403.hg.1	1.2	8.79	4.45	1.78E-05	6.37E-04	2.68	TC01003403.	chr1	-	160454820	160493052	155	SLAMF6	RefSeq
TC05000227.hg.1	1.2	7.01	2.78	6.15E-03	4.66E-02	-2.63	TC05000227.	chr5	+	54320081	54330398	83	GZMK	RefSeq
TC11001707.hg.1	1.2	3.31	3.5	6.19E-04	8.19E-03	-0.58	TC11001707.	chr11	-	45743090	45743119	6		GenBank
TC09002409.hg.1	1.2	5.35	4.47	1.60E-05	5.91E-04	2.78	TC09002409.	chr9	-	14606167	14609996	30		NONCODE
TC16000158.hg.1	1.2	8.53	5.24	6.02E-07	8.49E-05	5.83	TC16000158.	chr16	+	10971039	11023624	260	CIITA	RefSeq
TC15000063.hg.1	1.2	8.72	3.19	1.78E-03	1.82E-02	-1.53	TC15000063.	chr15	+	25328734	25328827	30	SNORD116-19	RefSeq
TC01005699.hg.1	1.2	8.61	4.62	8.64E-06	3.92E-04	3.35	TC01005699.	chr1	-	111197952	111199662	30		NONCODE
TC10000060.hg.1	1.2	6.22	3.09	2.42E-03	2.31E-02	-1.8	TC10000060.	chr10	+	6660678	6667308	36	LOC101928150	ENSEMBL
TC14002247.hg.1	1.2	6.97	2.74	7.01E-03	5.12E-02	-2.74	TC14002247.	chr14	-	106518400	106518932	30	IGHV3-7	ENSEMBL
TC6_mcf_hap5000076.hg.1	1.2	8.24	3.31	1.20E-03	1.35E-02	-1.17	TC6_mcf_ha	chr6_mcf_hap5	+	3934836	3955581	123	HLA-DQA1	GenBank
TC6_mann_hap4000170.hg.1	1.2	9.15	4.14	6.05E-05	1.47E-03	1.55	TC6_mann_h	chr6_mann_hap4	-	4489886	4506101	230	HLA-DPA1	RefSeq
TC6_qbl_hap6000190.hg.1	1.2	9.15	4.14	6.05E-05	1.47E-03	1.55	TC6_qbl_hap	chr6_qbl_hap6	-	4264797	4281012	230	HLA-DPA1	RefSeq
TC12001392.hg.1	1.2	6.81	4.34	2.70E-05	8.40E-04	2.3	TC12001392.	chr12	-	39943835	40013843	140	ABCD2	RefSeq
TC09002474.hg.1	1.2	7.31	4.54	1.19E-05	4.86E-04	3.05	TC09002474.	chr9	-	36840556	37026572	63	PAX5	NONCODE
TC01000834.hg.1	1.2	4.7	2.72	7.44E-03	5.37E-02	-2.79	TC01000834.	chr1	+	89726265	89735437	30	RP4-620F22.2	Havana
TC15000024.hg.1	1.2	6.35	2.94	3.91E-03	3.31E-02	-2.23	TC15000024.	chr15	+	22711945	22711970	2		GenBank
TC15000148.hg.1	1.2	6.35	2.94	3.91E-03	3.31E-02	-2.23	TC15000148.	chr15	+	28953531	28953556	2		GenBank
TC15000207.hg.1	1.2	6.35	2.94	3.91E-03	3.31E-02	-2.23	TC15000207.	chr15	+	32691459	32691484	2		GenBank
TC15000238.hg.1	1.2	6.35	2.94	3.91E-03	3.31E-02	-2.23	TC15000238.	chr15	+	34677951	34677976	2		GenBank
TC15000243.hg.1	1.2	6.35	2.94	3.91E-03	3.31E-02	-2.23	TC15000243.	chr15	+	34824172	34824197	2		GenBank
TC15001014.hg.1	1.2	6.35	2.94	3.91E-03	3.31E-02	-2.23	TC15001014.	chr15	-	20771430	20771455	2		GenBank
TC15001053.hg.1	1.2	6.35	2.94	3.91E-03	3.31E-02	-2.23	TC15001053.	chr15	-	23259259	23259284	2		GenBank
TC15001115.hg.1	1.2	6.35	2.94	3.91E-03	3.31E-02	-2.23	TC15001115.	chr15	-	30379164	30379189	2		GenBank
TC15001129.hg.1	1.2	6.35	2.94	3.91E-03	3.31E-02	-2.23	TC15001129.	chr15	-	30431264	30431289	2		GenBank
TC15001157.hg.1	1.2	6.35	2.94	3.91E-03	3.31E-02	-2.23	TC15001157.	chr15	-	31087687	31087712	2		GenBank
TC15001673.hg.1	1.2	6.35	2.94	3.91E-03	3.31E-02	-2.23	TC15001673.	chr15	-	76072677	76072702	2		GenBank
TC6_cox_hap2000202.hg.1	1.2	9.3	4.13	6.35E-05	1.52E-03	1.51	TC6_cox_ha	chr6_cox_hap2	-	4476526	4492734	219	HLA-DPA1	RefSeq
TC6_apd_hap1000107.hg.1	1.2	9.19	4.19	4.97E-05	1.29E-03	1.73	TC6_apd_ha	chr6_apd_hap1	-	4319165	4335362	230	HLA-DPA1	RefSeq
TC6_mcf_hap5000177.hg.1	1.2	9.19	4.19	4.97E-05	1.29E-03	1.73	TC6_mcf_ha	chr6_mcf_hap5	-	4369219	4385416	230	HLA-DPA1	RefSeq
TC15002209.hg.1	1.2	7.12	4.48	1.56E-05	5.82E-04	2.8	TC15002209.	chr15	+	57592039	57600717	268		NONCODE
TC02004912.hg.1	1.2	7.85	3.33	1.12E-03	1.28E-02	-1.11	TC02004912.	chr2	-	242961960	242968327	40		Broad
TC06001578.hg.1	1.2	9.19	4.19	5.02E-05	1.30E-03	1.72	TC06001578.	chr6	-	33032346	33048555	230	HLA-DPA1	RefSeq
TC6_dbb_hap3000188.hg.1	1.2	9.19	4.19	5.02E-05	1.30E-03	1.72	TC6_dbb_ha	chr6_dbb_hap3	-	4313685	4329894	230	HLA-DPA1	RefSeq
TC6_ssto_hap7000172.hg.1	1.2	9.21	4.2	4.74E-05	1.25E-03	1.78	TC6_ssto_ha	chr6_ssto_hap7	-	4512850	4529059	226	HLA-DPA1	RefSeq

TC02001199.hg.1	1.2	8.18	4.71	6.00E-06	3.14E-04	3.69	TC02001199.	chr2	+	204571198	204603636	160	CD28	RefSeq
TC0X000337.hg.1	1.2	4.83	3.79	2.21E-04	3.82E-03	0.36	TC0X000337.	chrX	+	56100757	56102014	30		ENSEMBL
TC02004860.hg.1	1.2	8.68	5.05	1.40E-06	1.35E-04	5.05	TC02004860.	chr2	-	235401690	235403788	30		NONCODE
TC14000123.hg.1	1.2	8.75	3.87	1.70E-04	3.16E-03	0.6	TC14000123.	chr14	+	22971215	22971276	30	TRAJ38	ENSEMBL
TC18000555.hg.1	1.2	6.21	3.49	6.40E-04	8.40E-03	-0.61	TC18000555.	chr18	-	60861822	60861898	30		ENSEMBL
TC07000918.hg.1	1.2	9.03	3.44	7.80E-04	9.77E-03	-0.78	TC07000918.	chr7	+	142448391	142448743	30		UCSC
TC01002849.hg.1	1.2	8.53	2.92	4.14E-03	3.45E-02	-2.28	TC01002849.	chr1	-	89646831	89664633	175	GBP4	RefSeq
TC14002250.hg.1	1.2	7.18	2.87	4.71E-03	3.80E-02	-2.39	TC14002250.	chr14	-	106573233	106573800	30	LOC100293211	ENSEMBL
TC19001116.hg.1	1.2	8.1	4.11	6.76E-05	1.59E-03	1.45	TC19001116.	chr19	-	7753643	7767032	150	FCER2	RefSeq
TC03000549.hg.1	1.2	6.5	5.13	9.61E-07	1.10E-04	5.4	TC03000549.	chr3	+	108541545	108573852	120	TRAT1	RefSeq
TC6_dbb_hap3000181.hg.1	1.2	8.1	4.75	5.10E-06	2.87E-04	3.84	TC6_dbb_ha	chr6_dbb_hap3	-	3905881	3915822	105	HLA-DQB1	GenBank
TC05003035.hg.1	1.2	7.75	4.22	4.38E-05	1.18E-03	1.85	TC05003035.	chr5	-	66478116	66492612	60	CD180	NONCODE
TC15002019.hg.1	1.2	6.24	3.32	1.17E-03	1.32E-02	-1.15	TC15002019.	chr15	-	102293649	102293678	6		GenBank
TC15002022.hg.1	1.2	6.24	3.32	1.17E-03	1.32E-02	-1.15	TC15002022.	chr15	-	102293993	102294022	6		GenBank
TC15002026.hg.1	1.2	6.24	3.32	1.17E-03	1.32E-02	-1.15	TC15002026.	chr15	-	102294519	102294548	6		GenBank
TC15002036.hg.1	1.2	6.24	3.32	1.17E-03	1.32E-02	-1.15	TC15002036.	chr15	-	102296006	102296035	6		GenBank
TC15002039.hg.1	1.2	6.24	3.32	1.17E-03	1.32E-02	-1.15	TC15002039.	chr15	-	102296341	102296370	6		GenBank
TC15002041.hg.1	1.2	6.24	3.32	1.17E-03	1.32E-02	-1.15	TC15002041.	chr15	-	102296958	102296987	6		GenBank
TC15002044.hg.1	1.2	6.24	3.32	1.17E-03	1.32E-02	-1.15	TC15002044.	chr15	-	102297484	102297513	6		GenBank
TC15002047.hg.1	1.2	6.24	3.32	1.17E-03	1.32E-02	-1.15	TC15002047.	chr15	-	102298348	102298377	6		GenBank
TC15002050.hg.1	1.2	6.24	3.32	1.17E-03	1.32E-02	-1.15	TC15002050.	chr15	-	102299219	102299248	6		GenBank
TC15002051.hg.1	1.2	6.24	3.32	1.17E-03	1.32E-02	-1.15	TC15002051.	chr15	-	102299836	102299865	6		GenBank
TC01003404.hg.1	1.2	6.33	4.12	6.52E-05	1.54E-03	1.48	TC01003404.	chr1	-	160473166	160473246	30		ENSEMBL
TC6_ssto_hap7000165.hg.1	1.2	8.05	4.57	1.08E-05	4.54E-04	3.15	TC6_ssto_ha	chr6_ssto_hap7	-	4055996	4063243	76	HLA-DQB1	GenBank
TC02001886.hg.1	1.2	6.77	4.26	3.72E-05	1.04E-03	2	TC02001886.	chr2	-	60751624	60755350	30		GenBank
TC03002724.hg.1	1.2	4.79	4.78	4.54E-06	2.69E-04	3.95	TC03002724.	chr3	+	191113180	191116458	30	CCDC50	NONCODE
TC02004397.hg.1	1.2	8.88	2.9	4.36E-03	3.59E-02	-2.32	TC02004397.	chr2	-	89416834	89417284	22		NONCODE
TC16001512.hg.1	1.2	6.42	2.93	4.01E-03	3.38E-02	-2.25	TC16001512.	chr16	+	32926412	32926831	20		Broad
TC02002962.hg.1	1.2	8.02	3.56	5.06E-04	7.03E-03	-0.39	TC02002962.	chr2	-	242967334	242968327	30	AC093642.4	Havana
TC01003366.hg.1	1.2	6	4.1	7.16E-05	1.66E-03	1.4	TC01003366.	chr1	-	157715521	157746922	189	FCRL2	RefSeq
TC17001455.hg.1	1.2	8.79	5.25	5.65E-07	8.19E-05	5.89	TC17001455.	chr17	-	37913968	38020441	126	IKZF3	RefSeq
TC12000599.hg.1	1.2	6.03	4.24	4.01E-05	1.10E-03	1.93	TC12000599.	chr12	+	68383225	68415107	40	IFNG-AS1	ENSEMBL
TC02000850.hg.1	1.2	7.43	4.12	6.43E-05	1.53E-03	1.49	TC02000850.	chr2	+	132143133	132143204	30		UCSC
TC03002723.hg.1	1.2	5.12	5.27	5.05E-07	7.73E-05	6	TC03002723.	chr3	+	191089936	191090896	30		NONCODE
TC13001576.hg.1	1.2	6.98	4.13	6.22E-05	1.50E-03	1.53	TC13001576.	chr13	-	74700603	74702042	30		NONCODE
TC14002191.hg.1	1.2	6.69	3.32	1.16E-03	1.32E-02	-1.14	TC14002191.	chr14	+	22918107	22934912	140	TRDJ1	ENSEMBL
TC14002265.hg.1	1.2	8.48	3.1	2.36E-03	2.26E-02	-1.78	TC14002265.	chr14	-	106845323	106845789	47	IGHV3-35	ENSEMBL
TC12002850.hg.1	1.3	7.26	5.03	1.48E-06	1.39E-04	4.99	TC12002850.	chr12	-	46581574	46581823	30	SLC38A1	NONCODE
TC04000524.hg.1	1.3	6.88	5.12	1.01E-06	1.13E-04	5.35	TC04000524.	chr4	+	102332443	102995969	318	BANK1	RefSeq
TC14002143.hg.1	1.3	7	3.84	1.90E-04	3.43E-03	0.5	TC14002143.	chr14	-	96176396	96176829	30		NONCODE
TC03000633.hg.1	1.3	8.04	4.6	9.54E-06	4.18E-04	3.26	TC03000633.	chr3	+	122296449	122357894	243	PARP15	RefSeq
TC22001005.hg.1	1.3	8.74	3.3	1.24E-03	1.39E-02	-1.2	TC22001005.	chr22	+	22735428	23247213	35		NONCODE
TC16000388.hg.1	1.3	9.68	2.9	4.33E-03	3.57E-02	-2.32	TC16000388.	chr16	+	32077386	32077674	30	IGHV3OR16-9	ENSEMBL
TC05001419.hg.1	1.3	7.6	4.37	2.45E-05	7.82E-04	2.38	TC05001419.	chr5	-	66478103	66492627	70	CD180	RefSeq
TC15001710.hg.1	1.3	9.88	3.65	3.73E-04	5.64E-03	-0.12	TC15001710.	chr15	-	79042403	79042433	7		GenBank
TC03001649.hg.1	1.3	7.1	5.27	5.06E-07	7.73E-05	6	TC03001649.	chr3	-	112182813	112218408	100	BTLA	RefSeq
TC15002028.hg.1	1.3	6.22	3.59	4.61E-04	6.57E-03	-0.31	TC15002028.	chr15	-	102294739	102294770	8		GenBank
TC15002031.hg.1	1.3	6.22	3.59	4.61E-04	6.57E-03	-0.31	TC15002031.	chr15	-	102295174	102295205	8		GenBank

TC15002034.hg.1	1.3	6.22	3.59	4.61E-04	6.57E-03	-0.31	TC15002034.	chr15	-	102295700	102295731	8		GenBank
TC15002043.hg.1	1.3	6.22	3.59	4.61E-04	6.57E-03	-0.31	TC15002043.	chr15	-	102297178	102297209	8		GenBank
TC15002046.hg.1	1.3	6.22	3.59	4.61E-04	6.57E-03	-0.31	TC15002046.	chr15	-	102297698	102297729	8		GenBank
TC15002049.hg.1	1.3	6.22	3.59	4.61E-04	6.57E-03	-0.31	TC15002049.	chr15	-	102298569	102298600	8		GenBank
TC02004258.hg.1	1.3	6.74	4.27	3.67E-05	1.03E-03	2.01	TC02004258.	chr2	-	60751633	60755350	30		NONCODE
TC10001555.hg.1	1.3	6.86	5.82	4.04E-08	1.98E-05	8.37	TC10001555.	chr10	-	97951455	98031333	280	BLNK	RefSeq
TC14002274.hg.1	1.3	7.62	3.67	3.42E-04	5.27E-03	-0.04	TC14002274.	chr14	-	107048672	107049341	30	IGHV3-53	ENSEMBL
TC14002256.hg.1	1.3	7.08	3.94	1.32E-04	2.60E-03	0.84	TC14002256.	chr14	-	106691673	106692203	20	IGHV3-21	ENSEMBL
TC16000385.hg.1	1.3	10.41	3.01	3.11E-03	2.79E-02	-2.02	TC16000385.	chr16	+	31973409	31985682	30		ENSEMBL
TC09001627.hg.1	1.3	5.92	2.85	5.03E-03	3.99E-02	-2.45	TC09001627.	chr9	-	131012390	131012416	3		GenBank
TC14002252.hg.1	1.3	7.68	2.79	6.09E-03	4.62E-02	-2.62	TC14002252.	chr14	-	106610313	106610852	30	IGHV3-15	ENSEMBL
TC01003367.hg.1	1.3	7.91	3.8	2.15E-04	3.75E-03	0.39	TC01003367.	chr1	-	157764193	157789940	226	FCRL1	RefSeq
TC12002432.hg.1	1.3	6.07	4.9	2.71E-06	1.98E-04	4.43	TC12002432.	chr12	+	68383225	68628466	150	IFNG-AS1	NONCODE
TC13000829.hg.1	1.3	8.35	4.75	5.13E-06	2.89E-04	3.84	TC13000829.	chr13	-	99946784	99959749	40	GPR183	RefSeq
TC14002272.hg.1	1.3	6.71	3.38	9.41E-04	1.13E-02	-0.95	TC14002272.	chr14	-	107012938	107013477	35	IGHV3-49	ENSEMBL
TC14002282.hg.1	1.3	6.17	2.76	6.50E-03	4.85E-02	-2.68	TC14002282.	chr14	-	107198932	107199471	20	IGHV3-72	ENSEMBL
TC14002228.hg.1	1.3	6.08	3.16	1.92E-03	1.93E-02	-1.59	TC14002228.	chr14	-	106361492	106361528	8	IGHD3-16	ENSEMBL
TC16001518.hg.1	1.3	8.17	3.04	2.86E-03	2.63E-02	-1.95	TC16001518.	chr16	+	33629698	33630084	17		Broad
TC14002263.hg.1	1.3	9.55	3.55	5.21E-04	7.18E-03	-0.42	TC14002263.	chr14	-	106815722	106816253	30	EPC1	ENSEMBL
TC12001202.hg.1	1.3	8	4.83	3.57E-06	2.35E-04	4.17	TC12001202.	chr12	-	9747147	9760497	70	KLRB1	RefSeq
TC0M000019.hg.1	1.3	8.51	3.64	3.83E-04	5.75E-03	-0.14	TC0M00001[illegible]	chrM	-	15960	16024	30		GenBank
TC17001269.hg.1	1.3	5.43	4.31	3.15E-05	9.31E-04	2.15	TC17001269.	chr17	-	22026337	22026405	30		GenBank
TC14002255.hg.1	1.3	6.04	2.88	4.62E-03	3.74E-02	-2.38	TC14002255.	chr14	-	106667581	106668095	23	IGHV3-20	ENSEMBL
TC16000387.hg.1	1.3	8.52	2.75	6.73E-03	4.98E-02	-2.71	TC16000387.	chr16	+	32070548	32070635	30		UCSC
TC14002279.hg.1	1.3	7.68	3.36	9.94E-04	1.17E-02	-1	TC14002279.	chr14	-	107131033	107131560	30	IGHV3-66	ENSEMBL
TC22001009.hg.1	1.3	9.18	2.75	6.81E-03	5.01E-02	-2.72	TC22001009.	chr22	+	23261707	23262023	30		NONCODE
TC02004432.hg.1	1.3	3.9	3.84	1.85E-04	3.36E-03	0.52	TC02004432.	chr2	-	100705115	100705776	30	AC092168.4	NONCODE
TC02003402.hg.1	1.3	7.9	2.84	5.18E-03	4.08E-02	-2.48	TC02003402.	chr2	+	90198907	90199183	30		NONCODE
TC04000362.hg.1	1.3	6.01	5.21	6.75E-07	9.09E-05	5.73	TC04000362.	chr4	+	68424446	68473055	120	STAP1	RefSeq
TC19002611.hg.1	1.3	8.63	4.79	4.35E-06	2.63E-04	3.99	TC19002611.	chr19	+	35820072	35838264	190	CD22	RefSeq
TC16000396.hg.1	1.3	8.92	3.28	1.33E-03	1.47E-02	-1.27	TC16000396.	chr16	+	33006369	33006839	30	IGHV3OR16-10	ENSEMBL
TC14002213.hg.1	1.3	9.76	4.43	1.90E-05	6.62E-04	2.62	TC14002213.	chr14	-	106320349	106322323	50	IGHM	ENSEMBL
TC14002268.hg.1	1.3	7.55	3.14	2.05E-03	2.03E-02	-1.65	TC14002268.	chr14	-	106926188	106926724	30	IGHV3-43	ENSEMBL
TC6_ssto_hap7000077.hg.1	1.3	8.74	2.8	5.84E-03	4.49E-02	-2.58	TC6_ssto_ha	chr6_ssto_hap7	+	4038586	4044828	63	HLA-DQA1	GenBank
TC05003326.hg.1	1.3	4.69	4.82	3.69E-06	2.40E-04	4.14	TC05003326.	chr5	-	158470618	158472793	30		NONCODE
TC14002261.hg.1	1.3	8.64	3.26	1.42E-03	1.54E-02	-1.32	TC14002261.	chr14	-	106791005	106791536	40	IGHV3-30	ENSEMBL
TC19000588.hg.1	1.3	9.03	4.79	4.25E-06	2.60E-04	4.01	TC19000588.	chr19	+	42381190	42385439	80	CD79A	RefSeq
TC22001449.hg.1	1.3	9.39	2.75	6.81E-03	5.02E-02	-2.72	TC22001449.	chr22	+	23264765	23265085	10	IGLC7	ENSEMBL
TC14002257.hg.1	1.3	8.58	2.84	5.13E-03	4.06E-02	-2.47	TC14002257.	chr14	-	106725201	106725733	30	IGHV3-23	ENSEMBL
TC01002064.hg.1	1.3	8.32	3.67	3.48E-04	5.35E-03	-0.05	TC01002064.	chr1	-	566187	566265	29		ENSEMBL
TC22000467.hg.1	1.4	9	2.74	6.91E-03	5.08E-02	-2.73	TC22000467.	chr22	-	17385315	17385395	30		UCSC
TC14002234.hg.1	1.4	5.88	3.32	1.14E-03	1.31E-02	-1.13	TC14002234.	chr14	-	106370355	106370385	6	IGHD3-10	ENSEMBL
TC14002214.hg.1	1.4	8.15	4.79	4.25E-06	2.60E-04	4.01	TC14002214.	chr14	-	106326591	106329064	60	IGHA1	UCSC
TC11000510.hg.1	1.4	8.29	5.26	5.44E-07	8.10E-05	5.93	TC11000510.	chr11	+	60223282	60238233	168	MS4A1	RefSeq
TC14002241.hg.1	1.5	4.07	3.15	1.99E-03	1.99E-02	-1.63	TC14002241.	chr14	-	106382685	106382715	6	IGHD2-2	ENSEMBL
TC14002240.hg.1	1.5	5.49	3.47	7.03E-04	9.02E-03	-0.69	TC14002240.	chr14	-	106380218	106380248	6	IGHD3-3	ENSEMBL
