# Supplementary material for: Transcriptome-wide study of TNF-inhibitor therapy in rheumatoid arthritis reveals early signature of successful treatment
Source: Arthritis Res Ther. 2021 Mar 10;23:80. doi: 10.1186/s13075-021-02451-9 (PMC7948368; doi:10.1186/s13075-021-02451-9)
Supplement: Supplementary file 2 — Additional file 2: Table S2. The differentially expressed genes in adalimumab good-responders (baseline versus 3-months) as analysed using Ingenuity Pathway Analysis (IPA) software. The -log(p-value) for each pathway association, the ratio of the number of differentially expressed genes within each pathway, and a list of the differentially expressed genes (molecules) within each pathway are shown. [file 13075_2021_2451_MOESM2_ESM.pdf]

Ingenuity Canonical Pathways	-LOG(p-value)	Ratio	Molecules
Altered T Cell and B Cell Signaling in Rheumatoid Arthritis	9.85	0.173	TLR2,CD28,TLR4,CD40LG,HLA-DOA,HLA-DRB1,TLR5,TLR6,TLR8,HLA-DQA1,HLA-DOB,IL1B,CD79A,HLA-DQB1
TREM1 Signaling	9.6	0.186	TREM1,ICAM1,CIITA,TLR8,TLR2,TLR4,TLR5,NLRP12,PLCG2,TLR6,IL1B,NLRC4,ITGAX
Dendritic Cell Maturation	7.25	0.0947	HLA-DOA,CD40LG,ICAM1,HLA-DQA1,CD58,IGHG1,HLA-DQB1,CREB5,TLR2,TLR4,MAPK14,HLA-DRB1,PLCG2,IL1B,HLA-DOB,CD1C
Allograft Rejection Signaling	6.86	0.188	CD28,HLA-DOA,CD40LG,HLA-DRB1,HLA-DQA1,HLA-DOB,IGHG1,HLA-DQB1,HLA-DPA1
Communication between Innate and Adaptive Immune Cells	6.69	0.134	TLR2,CD28,TLR4,CD40LG,HLA-DRB1,TLR5,TLR6,TLR8,IL1B,IGHA1,IGHG1
Nur77 Signaling in T Lymphocytes	6.62	0.176	CD3G,CD28,HLA-DOA,HLA-DRB1,HLA-DQA1,HLA-DOB,HLA-DQB1,BCL2,MAP3K2
phagosome formation	6.6	0.118	TLR2,FCAR,MARCKS,TLR4,TLR5,PLCG2,TLR6,FCER1A,TLR8,IGHG1,ITGA4,FCER2
iCOS-iCOSL Signaling in T Helper Cells	6.55	0.117	CD3G,GAB2,CD28,HLA-DOA,CD40LG,HLA-DRB1,TRAT1,HLA-DQA1,HLA-DOB,HLA-DQB1,ITK,PTEN
B Cell Development	6.48	0.259	HLA-DOA,HLA-DRB1,IGH,HLA-DQA1,HLA-DOB,CD79A,HLA-DQB1
B Cell Receptor Signaling	6.4	0.0877	ETS1,BLNK,GAB2,SOS2,CD79A,IGHG1,BCL6,CREB5,PTEN,PAX5,MAPK14,PLCG2,CD22,BCL2A1,MAP3K2
OX40 Signaling Pathway	6.4	0.167	CD3G,HLA-DOA,HLA-DRB1,HLA-DQA1,HLA-DOB,TRAF5,HLA-DQB1,HLA-DPA1,BCL2
Autoimmune Thyroid Disease Signaling	6.21	0.19	CD28,HLA-DOA,CD40LG,HLA-DRB1,HLA-DQA1,HLA-DOB,IGHG1,HLA-DQB1
NF-_B Signaling	5.71	0.0828	CD40LG,TLR8,IRAK3,IL1R1,IGF2R,TLR2,IL1R2,TLR4,TLR5,PLCG2,TLR6,IGF1R,IL1B,TRAF5
Type I Diabetes Mellitus Signaling	5.55	0.104	CD3G,CD28,HLA-DOA,HLA-DRB1,MAPK14,HLA-DQA1,HLA-DOB,IL1B,HLA-DQB1,IL1R1,BCL2
Toll-like Receptor Signaling	5.26	0.123	TLR2,TLR4,LY96,MAPK14,TLR5,TLR6,TLR8,IL1B,IRAK3
Granulocyte Adhesion and Diapedesis	5.11	0.0788	IL1R2,HRH2,ICAM1,C5AR1,MMP8,FPR2,IL1B,IL1R1,MMP25,MMP9,ITGA4,IL18RAP,FPR1
Role of NFAT in Regulation of the Immune Response	5.08	0.0783	BLNK,CD28,CD3G,HLA-DOA,HLA-DRB1,PLCG2,SOS2,FCER1A,HLA-DQA1,HLA-DOB,CD79A,HLA-DQB1,ITK
Primary Immunodeficiency Signaling	4.95	0.159	BLNK,CD40LG,IGH,CIITA,CD79A,IGHA1,IGHG1
Graft-versus-Host Disease Signaling	4.95	0.159	CD28,HLA-DOA,HLA-DRB1,HLA-DQA1,HLA-DOB,IL1B,HLA-DQB1
Role of Macrophages, Fibroblasts and Endothelial Cells in Rheumatoid Arthritis	4.82	0.0592	ICAM1,C5AR1,TLR8,IGHG1,IRAK3,IL1R1,CREB5,IL18RAP,TLR2,IL1R2,TLR4,MAPK14,TLR5,PLCG2,TLR6,IL1B,TRAF5
Cdc42 Signaling	4.73	0.0853	CD3G,HLA-DOA,HLA-DRB1,MAPK14,HLA-DQA1,HLA-DOB,LIMK2,HLA-DQB1,HLA-DPA1,ITGA4,ITK
T Helper Cell Differentiation	4.63	0.119	CD28,HLA-DOA,CD40LG,HLA-DRB1,HLA-DQA1,HLA-DOB,HLA-DQB1,BCL6
PKC_ Signaling in T Lymphocytes	4.49	0.0885	CD3G,CD28,HLA-DOA,HLA-DRB1,PLCG2,SOS2,HLA-DQA1,HLA-DOB,HLA-DQB1,MAP3K2
IL-4 Signaling	4.4	0.111	HLA-DOA,HLA-DRB1,IGH,SOS2,HLA-DQA1,HLA-DOB,HLA-DQB1,FCER2
Antigen Presentation Pathway	4.37	0.162	HLA-DOA,HLA-DRB1,CIITA,HLA-DQA1,HLA-DOB,HLA-DPA1
Role of Pattern Recognition Receptors in Recognition of Bacteria and Viruses	4.29	0.084	TLR2,TLR4,C5AR1,OAS2,TLR5,PLCG2,TLR6,TLR8,IL1B,NLRC4

LXR/RXR Activation	4.23	0.0826	IL1R2,TLR4,LY96,ORM1,ORM2,IL1B,IL1R1,ABCA1,MMP9,IL18RAP
Hepatic Fibrosis / Hepatic Stellate Cell Activation	4.02	0.0663	IL1R2,TLR4,CD40LG,LY96,ICAM1,IGF1R,IL1B,ECE1,IL1R1,MMP9,IL18RAP,BCL2
Atherosclerosis Signaling	3.52	0.0744	CD40LG,ICAM1,ORM1,ORM2,IL1B,ALOX5,TNFSF14,MMP9,ITGA4
Calcium-induced T Lymphocyte Apoptosis	3.26	0.103	CD3G,HLA-DOA,HLA-DRB1,HLA-DQA1,HLA-DOB,HLA-DQB1
Leukocyte Extravasation Signaling	3.17	0.057	ICAM1,MAPK14,PLCG2,RASGRP1,MMP8,BMX,NCF4,MMP25,MMP9,ITGA4,ITK
CD28 Signaling in T Helper Cells	3.04	0.0708	CD3G,CD28,HLA-DOA,HLA-DRB1,HLA-DQA1,HLA-DOB,HLA-DQB1,ITK
LPS/IL-1 Mediated Inhibition of RXR Function	2.91	0.0529	IL1R2,TLR4,LY96,ACOX1,IL1B,IL1R1,CHST15,SULT1B1,ABCA1,ACSL1,IL18RAP
_-tocopherol Degradation	2.74	0.5	CYP4F3,CYP4F2
CDP-diacylglycerol Biosynthesis I	2.59	0.188	ABHD5,MBOAT2,GPAT3
Triacylglycerol Biosynthesis	2.58	0.121	ABHD5,DGAT2,MBOAT2,GPAT3
PPAR_/RXR_ Activation	2.56	0.0545	IL1R2,MAPK14,PLCG2,ACOX1,SOS2,IL1B,IL1R1,ABCA1,IL18RAP
Protein Citrullination	2.52	0.4	PADI4,PADI2
Complement System	2.44	0.111	SERPING1,CR1,C5AR1,ITGAX
Phosphatidylglycerol Biosynthesis II (Non-plastidic)	2.43	0.167	ABHD5,MBOAT2,GPAT3
Systemic Lupus Erythematosus Signaling	2.42	0.0485	CD3G,CD28,CD40LG,PLCG2,IGH,SOS2,CD22,IL1B,CD79A,IGHG1
Agranulocyte Adhesion and Diapedesis	2.39	0.0514	ICAM1,C5AR1,MMP8,IL1B,CXCR1,IL1R1,MMP25,MMP9,ITGA4
IL-6 Signaling	2.34	0.0603	IL1R2,MAPK14,TNFAIP6,SOS2,IL1B,IL1R1,IL18RAP
p38 MAPK Signaling	2.32	0.0598	IL1R2,MAPK14,IL1B,IRAK3,IL1R1,CREB5,IL18RAP
Role of Osteoblasts, Osteoclasts and Chondrocytes in Rheumatoid Arthritis	2.31	0.0467	IL1R2,MAPK14,MMP8,IL1B,TRAF5,IL1R1,BIRC3,ALPL,IL18RAP,BCL2
CD40 Signaling	2.26	0.0781	CD40LG,ICAM1,MAPK14,TRAF5,FCER2
PI3K Signaling in B Lymphocytes	2.2	0.0569	BLNK,TLR4,CD180,PLCG2,CD79A,IRS2,PTEN
Hematopoiesis from Pluripotent Stem Cells	2.2	0.0952	CD3G,IGH,IGHA1,IGHG1
iNOS Signaling	2.16	0.093	TLR4,LY96,MAPK14,IRAK3
IL-10 Signaling	2.15	0.0735	IL1R2,MAPK14,IL1B,IL1R1,IL18RAP
T Cell Receptor Signaling	2.14	0.0625	CD3G,CD28,RASGRP1,SOS2,BMX,ITK
Airway Pathology in Chronic Obstructive Pulmonary Disease	2.09	0.25	MMP8,MMP9
Salvage Pathways of Pyrimidine Deoxyribonucleotides	2.09	0.25	CDA,APOBEC3A

Phospholipase C Signaling	2.08	0.0433	BLNK,MARCKS,CD3G,PLCG2,SOS2,CD79A,IGHG1,CREB5,ITGA4,ITK
Caveolar-mediated Endocytosis Signaling	2.07	0.0704	FLOT2,FLOT1,ITGA4,ITGAX,MAP3K2
Acute Phase Response Signaling	1.99	0.0476	SERPING1,HP,MAPK14,ORM1,SOS2,ORM2,IL1B,IL1R1
Production of Nitric Oxide and Reactive Oxygen Species in Macrophages	1.84	0.0447	TLR2,TLR4,MAPK14,ORM1,PLCG2,ORM2,NCF4,MAP3K2
IL-8 Signaling	1.78	0.0437	ICAM1,DEFA1 (includes others),LIMK2,IRAK3,CXCR1,MMP9,ITGAX,BCL2
Glycogen Degradation III	1.74	0.167	PYGL,MGAM
PTEN Signaling	1.73	0.0508	SOS2,IGF1R,IGF2R,ITGA4,BCL2,PTEN
Tec Kinase Signaling	1.69	0.0452	TLR4,PLCG2,IGH,FCER1A,BMX,ITGA4,ITK
Crosstalk between Dendritic Cells and Natural Killer Cells	1.68	0.0562	CD28,TLR4,CD40LG,KLRC4-KLRK1/KLRK1,HLA-DRB1
PPAR Signaling	1.66	0.0556	IL1R2,SOS2,IL1B,IL1R1,IL18RAP
Colorectal Cancer Metastasis Signaling	1.65	0.039	TLR2,TLR4,TLR5,MMP8,TLR6,SOS2,TLR8,MMP25,MMP9
Hepatic Cholestasis	1.65	0.0443	IL1R2,TLR4,LY96,IL1B,IRAK3,IL1R1,IL18RAP
GM-CSF Signaling	1.62	0.0645	ETS1,CSF2RA,SOS2,BCL2A1
Glioma Signaling	1.54	0.0515	PLCG2,SOS2,IGF1R,IGF2R,PTEN
IL-12 Signaling and Production in Macrophages	1.54	0.0458	TLR2,TLR4,CD40LG,MAPK14,ORM1,ORM2
Inhibition of Matrix Metalloproteases	1.53	0.0789	MMP8,MMP25,MMP9
Docosahexaenoic Acid (DHA) Signaling	1.5	0.0769	IL1B,BCL2A1,BCL2
HIF1_ Signaling	1.49	0.05	MAPK14,MMP8,MMP25,SLC2A3,MMP9
Neuroprotective Role of THOP1 in Alzheimer's Disease	1.48	0.075	MME,ECE1,MMP9
Fc_RIIB Signaling in B Lymphocytes	1.45	0.0732	BLNK,PLCG2,CD79A
FLT3 Signaling in Hematopoietic Progenitor Cells	1.43	0.0563	GAB2,MAPK14,SOS2,CREB5
G-Protein Coupled Receptor Signaling	1.43	0.0354	HCAR3,HRH2,RGS2,RASGRP1,SOS2,FPR2,CREB5,HCAR2,FPR1
HGF Signaling	1.43	0.0481	ETS1,PLCG2,SOS2,ITGA4,MAP3K2
STAT3 Pathway	1.39	0.0548	MAPK14,IGF1R,IGF2R,BCL2
Role of Tissue Factor in Cancer	1.38	0.0467	MAPK14,PLAUR,IL1B,LIMK2,PTEN
Fc Epsilon RI Signaling	1.37	0.0463	MAPK14,PLCG2,IGH,SOS2,FCER1A
G_s Signaling	1.37	0.0463	HCAR3,HRH2,RGS2,CREB5,HCAR2

D-myo-inositol (1,3,4)-trisphosphate Biosynthesis	1.36	0.105	IPMK,PTEN
Regulation of IL-2 Expression in Activated and Anergic T Lymphocytes	1.31	0.0513	CD3G,CD28,PLCG2,SOS2
1D-myo-inositol Hexakisphosphate Biosynthesis V (from Ins(1,3,4)P3)	1.28	0.333	IPMK
Prostate Cancer Signaling	1.27	0.05	SOS2,CREB5,BCL2,PTEN
HMGB1 Signaling	1.23	0.0424	TLR4,ICAM1,MAPK14,IL1B,IL1R1
Melanocyte Development and Pigmentation Signaling	1.21	0.0476	PLCG2,SOS2,CREB5,BCL2
PI3K/AKT Signaling	1.19	0.0413	GAB2,SOS2,ITGA4,BCL2,PTEN
Neuregulin Signaling	1.18	0.0465	PLCG2,SOS2,ITGA4,PTEN
FAK Signaling	1.18	0.0465	PLCG2,SOS2,ITGA4,PTEN
RANK Signaling in Osteoclasts	1.18	0.0465	MAPK14,TRAF5,BIRC3,MAP3K2
Superpathway of D-myo-inositol (1,4,5)-trisphosphate Metabolism	1.18	0.0833	IPMK,PTEN
Pentose Phosphate Pathway (Oxidative Branch)	1.16	0.25	PGD
Arginine Degradation I (Arginase Pathway)	1.16	0.25	ARG1
NAD Biosynthesis III	1.16	0.25	NAMPT
Acetate Conversion to Acetyl-CoA	1.16	0.25	ACSL1
Apoptosis Signaling	1.15	0.0455	PLCG2,BCL2A1,BIRC3,BCL2
Thrombopoietin Signaling	1.13	0.0545	GAB2,PLCG2,IRS2
Salvage Pathways of Pyrimidine Ribonucleotides	1.11	0.044	LIMK2,MAK,CDA,APOBEC3A
Myc Mediated Apoptosis Signaling	1.08	0.0517	SOS2,IGF1R,BCL2
Antioxidant Action of Vitamin C	1.06	0.0421	MAPK14,CSF2RA,PLCG2,SLC2A3
cAMP-mediated signaling	1.05	0.0324	HCAR3,HRH2,RGS2,FPR2,CREB5,HCAR2,FPR1
Paxillin Signaling	1.02	0.0408	MAPK14,SOS2,ITGA4,ITGAX
Eicosanoid Signaling	1.01	0.0484	FPR2,ALOX5AP,ALOX5
D-myo-inositol-5-phosphate Metabolism	1.01	0.0362	PIP4P2,PLCG2,PPP4R1,ALPL,PTEN
Hypoxia Signaling in the Cardiovascular System	0.997	0.0476	CREB5,UBE2D1,PTEN
Proline Biosynthesis II (from Arginine)	0.993	0.167	PADI4
Urea Cycle	0.993	0.167	ARG1

Arginine Degradation VI (Arginase 2 Pathway)	0.993	0.167	ARG1
Cytotoxic T Lymphocyte-mediated Apoptosis of Target Cells	0.984	0.0645	CD3G,BCL2
4-1BB Signaling in T Lymphocytes	0.984	0.0645	TNFRSF9,MAPK14
Inhibition of Angiogenesis by TSP1	0.961	0.0625	MAPK14,MMP9
Glucocorticoid Receptor Signaling	0.955	0.0294	IL1R2,CD3G,ICAM1,MAPK14,SOS2,SLPI,IL1B,BCL2
Superpathway of Inositol Phosphate Compounds	0.946	0.0323	IPMK,PIP4P2,PLCG2,PPP4R1,ALPL,PTEN
MIF-mediated Glucocorticoid Regulation	0.938	0.0606	TLR4,LY96
CCR5 Signaling in Macrophages	0.938	0.0448	CD3G,MAPK14,PLCG2
Inositol Pyrophosphates Biosynthesis	0.93	0.143	IPMK
Tryptophan Degradation to 2-amino-3-carboxymuconate Semialdehyde	0.93	0.143	KMO
NGF Signaling	0.925	0.0377	PLCG2,SOS2,CREB5,MAP3K2
GDNF Family Ligand-Receptor Interactions	0.923	0.0441	PLCG2,SOS2,IRS2
Chemokine Signaling	0.923	0.0441	MAPK14,PLCG2,LIMK2
Natural Killer Cell Signaling	0.904	0.037	KLRC4-KLRK1/KLRK1,PLCG2,KLRB1,SOS2
Coagulation System	0.896	0.0571	F5,PLAUR
Small Cell Lung Cancer Signaling	0.883	0.0423	TRAF5,BCL2,PTEN
PEDF Signaling	0.883	0.0423	MAPK14,TCF7,BCL2
autophagy	0.876	0.0556	W DFY3,BCL2
Histidine Degradation III	0.876	0.125	HAL
Citrulline Biosynthesis	0.876	0.125	ARG1
Prolactin Signaling	0.857	0.0411	PLCG2,SOS2,TCF7
Integrin Signaling	0.84	0.03	PLCG2,SOS2,TTN,ITGA4,ITGAX,PTEN
April Mediated Signaling	0.838	0.0526	MAPK14,TRAF5
Role of PKR in Interferon Induction and Antiviral Response	0.803	0.05	MAPK14,TRAF5
B Cell Activating Factor Signaling	0.803	0.05	MAPK14,TRAF5
Pentose Phosphate Pathway	0.786	0.1	PGD
Glycogen Degradation II	0.786	0.1	PYGL

Histidine Degradation VI	0.786	0.1	HAL
G_i Signaling	0.786	0.0333	SOS2,FPR2,HCAR2,FPR1
MIF Regulation of Innate Immunity	0.786	0.0488	TLR4,LY96
Role of IL-17F in Allergic Inflammatory Airway Diseases	0.786	0.0488	IL1B,CREB5
Dermatan Sulfate Biosynthesis (Late Stages)	0.77	0.0476	CHST15,SULT1B1
D-myo-inositol (1,4,5,6)-Tetrakisphosphate Biosynthesis	0.768	0.0328	IPMK,PPP4R1,ALPL,PTEN
D-myo-inositol (3,4,5,6)-tetrakisphosphate Biosynthesis	0.768	0.0328	IPMK,PPP4R1,ALPL,PTEN
Chondroitin Sulfate Biosynthesis (Late Stages)	0.738	0.0455	CHST15,SULT1B1
GNRH Signaling	0.726	0.0315	MAPK14,SOS2,CREB5,MAP3K2
_-Adrenergic Signaling	0.72	0.0353	PLCG2,PYGL,SLC8A1
ErbB Signaling	0.72	0.0353	MAPK14,PLCG2,SOS2
FGF Signaling	0.72	0.0353	MAPK14,SOS2,CREB5
Bladder Cancer Signaling	0.71	0.0349	MMP8,MMP25,MMP9
MSP-RON Signaling Pathway	0.709	0.0435	TLR2,TLR4
CTLA4 Signaling in Cytotoxic T Lymphocytes	0.7	0.0345	CD3G,CD28,TRAT1
UVA-Induced MAPK Signaling	0.7	0.0345	MAPK14,PARP15,PLCG2
TGF-_ Signaling	0.7	0.0345	MAPK14,SOS2,BCL2
Role of NFAT in Cardiac Hypertrophy	0.695	0.0284	MAPK14,PLCG2,SOS2,IGF1R,SLC8A1
PAK Signaling	0.69	0.0341	SOS2,LIMK2,ITGA4
Fatty Acid Activation	0.684	0.0769	ACSL1
NAD biosynthesis II (from tryptophan)	0.684	0.0769	KMO
Heparan Sulfate Biosynthesis (Late Stages)	0.681	0.0417	CHST15,SULT1B1
VEGF Signaling	0.68	0.0337	PLCG2,SOS2,BCL2
IL-1 Signaling	0.662	0.033	MAPK14,IRAK3,IL1R1
Death Receptor Signaling	0.662	0.033	PARP15,BIRC3,BCL2
Superpathway of Citrulline Metabolism	0.655	0.0714	ARG1
Leukotriene Biosynthesis	0.655	0.0714	ALOX5

_-glutamyl Cycle	0.655	0.0714	ANPEP
Vitamin-C Transport	0.655	0.0714	SLC2A3
CD27 Signaling in Lymphocytes	0.642	0.0392	TRAF5,MAP3K2
3-phosphoinositide Degradation	0.634	0.0288	PIP4P2,PPP4R1,ALPL,PTEN
ERK/MAPK Signaling	0.631	0.0269	ETS1,PLCG2,SOS2,CREB5,ITGA4
Endometrial Cancer Signaling	0.63	0.0385	SOS2,PTEN
Chondroitin Sulfate Biosynthesis	0.63	0.0385	CHST15,SULT1B1
Oxidative Ethanol Degradation III	0.629	0.0667	ACSL1
Unfolded protein response	0.618	0.0377	DNAJC3,BCL2
Regulation of eIF4 and p70S6K Signaling	0.613	0.0282	MAPK14,SOS2,AGO4,ITGA4
IGF-1 Signaling	0.609	0.0309	SOS2,IGF1R,IRS2
Lymphotoxin _ Receptor Signaling	0.606	0.037	TRAF5,TNFSF14
Granzyme B Signaling	0.605	0.0625	LMNB1
Extrinsic Prothrombin Activation Pathway	0.605	0.0625	F5
Parkinson's Signaling	0.605	0.0625	MAPK14
p53 Signaling	0.601	0.0306	MAPK14,BCL2,PTEN
Heparan Sulfate Biosynthesis	0.595	0.0364	CHST15,SULT1B1
Dermatan Sulfate Biosynthesis	0.595	0.0364	CHST15,SULT1B1
Wnt/Ca+ pathway	0.595	0.0364	PLCG2,CREB5
Glioblastoma Multiforme Signaling	0.593	0.0276	PLCG2,SOS2,IGF1R,PTEN
Actin Nucleation by ARP-WASP Complex	0.584	0.0357	SOS2,ITGA4
ErbB2-ErbB3 Signaling	0.584	0.0357	SOS2,PTEN
EGF Signaling	0.584	0.0357	MAPK14,SOS2
_-linolenate Biosynthesis II (Animals)	0.582	0.0588	ACSL1
Mitochondrial L-carnitine Shuttle Pathway	0.582	0.0588	ACSL1
Cholecystokinin/Gastrin-mediated Signaling	0.577	0.0297	MAPK14,SOS2,IL1B
Glioma Invasiveness Signaling	0.573	0.0351	PLAUR,MMP9

ErbB4 Signaling	0.562	0.0345	PLCG2,SOS2
Differential Regulation of Cytokine Production in Macrophages and T Helper Cells by IL-17A and IL-17F	0.561	0.0556	IL1B
Induction of Apoptosis by HIV1	0.552	0.0339	BIRC3,BCL2
ATM Signaling	0.552	0.0339	MAPK14,CREB5
Role of JAK1 and JAK3 in _c Cytokine Signaling	0.542	0.0333	BLNK,IRS2
1D-myo-inositol Hexakisphosphate Biosynthesis II (Mammalian)	0.541	0.0526	IPMK
Ethanol Degradation IV	0.541	0.0526	ACSL1
Corticotropin Releasing Hormone Signaling	0.532	0.028	MAPK14,PLCG2,CREB5
Renin-Angiotensin Signaling	0.525	0.0278	MAPK14,PLCG2,SOS2
Tryptophan Degradation III (Eukaryotic)	0.522	0.05	KMO
Xenobiotic Metabolism Signaling	0.522	0.0234	MAPK14,NQO2,IL1B,CHST15,SULT1B1,MAP3K2
Dopamine-DARPP32 Feedback in cAMP Signaling	0.52	0.0255	KCNJ2,PLCG2,KCNJ15,CREB5
ERK5 Signaling	0.513	0.0317	CREB5,MAP3K2
Estrogen-Dependent Breast Cancer Signaling	0.513	0.0317	IGF1R,CREB5
Pyridoxal 5'-phosphate Salvage Pathway	0.513	0.0317	LIMK2,MAK
Endoplasmic Reticulum Stress Pathway	0.505	0.0476	DNAJC3
IL-17A Signaling in Airway Cells	0.504	0.0312	MAPK14,PTEN
Actin Cytoskeleton Signaling	0.502	0.0238	TIAM2,SOS2,LIMK2,TTN,ITGA4
Polyamine Regulation in Colon Cancer	0.488	0.0455	MXD1
Dopamine Degradation	0.488	0.0455	SULT1B1
Erythropoietin Signaling	0.478	0.0299	PLCG2,SOS2
Role of MAPK Signaling in the Pathogenesis of Influenza	0.478	0.0299	MAPK14,BCL2
Neurotrophin/TRK Signaling	0.478	0.0299	SOS2,CREB5
Differential Regulation of Cytokine Production in Intestinal Epithelial Cells by IL-17A and IL-17F	0.473	0.0435	IL1B
Melatonin Signaling	0.47	0.0294	RORA,PLCG2
Growth Hormone Signaling	0.461	0.029	PLCG2,IGF1R
Renal Cell Carcinoma Signaling	0.461	0.029	ETS1,SOS2

p70S6K Signaling	0.459	0.0254	PLCG2,SOS2,CD79A
IL-22 Signaling	0.458	0.0417	MAPK14
Lipid Antigen Presentation by CD1	0.458	0.0417	CD1C
Tumoricidal Function of Hepatic Natural Killer Cells	0.458	0.0417	ICAM1
Glutathione-mediated Detoxification	0.458	0.0417	ANPEP
RhoA Signaling	0.447	0.025	IGF1R,LIMK2,TTN
IL-17A Signaling in Gastric Cells	0.444	0.04	MAPK14
Role of JAK family kinases in IL-6-type Cytokine Signaling	0.444	0.04	MAPK14
Ephrin Receptor Signaling	0.441	0.0233	SOS2,LIMK2,CREB5,ITGA4
Sertoli Cell-Sertoli Cell Junction Signaling	0.436	0.0231	MAPK14,ITGA4,MAP3K2,PTEN
LPS-stimulated MAPK Signaling	0.43	0.0274	TLR4,MAPK14
D-myo-inositol (1,4,5)-Trisphosphate Biosynthesis	0.43	0.0385	PLCG2
Pyrimidine Ribonucleotides Interconversion	0.43	0.0385	ENTPD1
FXR/RXR Activation	0.419	0.024	ORM1,ORM2,IL1B
NRF2-mediated Oxidative Stress Response	0.417	0.0226	MAPK14,NQO2,DNAJC3,JUNB
AMPK Signaling	0.417	0.0226	PFKFB3,MAPK14,IRS2,CREB5
VEGF Family Ligand-Receptor Interactions	0.409	0.0263	PLCG2,SOS2
TNFR2 Signaling	0.405	0.0357	BIRC3
Intrinsic Prothrombin Activation Pathway	0.405	0.0357	F5
Pyrimidine Ribonucleotides De Novo Biosynthesis	0.405	0.0357	ENTPD1
Insulin Receptor Signaling	0.403	0.0234	SOS2,IRS2,PTEN
PDGF Signaling	0.402	0.026	PLCG2,SOS2
ILK Signaling	0.4	0.0221	IRS2,CREB5,MMP9,PTEN
Acute Myeloid Leukemia Signaling	0.395	0.0256	CSF2RA,SOS2
Thyroid Hormone Metabolism II (via Conjugation and/or Degradation)	0.393	0.0345	SULT1B1
Ovarian Cancer Signaling	0.393	0.0231	MMP9,BCL2,PTEN
Clathrin-mediated Endocytosis Signaling	0.387	0.0217	ORM1,NUMB,ORM2,HIP1

Ethanol Degradation II	0.382	0.0333	ACSL1
Fatty Acid _-oxidation I	0.382	0.0333	ACSL1
G Protein Signaling Mediated by Tubby	0.371	0.0323	PLCG2
GPCR-Mediated Nutrient Sensing in Enteroendocrine Cells	0.363	0.0241	PLCG2,FFAR2
Role of JAK2 in Hormone-like Cytokine Signaling	0.361	0.0312	IRS2
Circadian Rhythm Signaling	0.351	0.0303	CREB5
TWEAK Signaling	0.351	0.0303	BIRC3
Molecular Mechanisms of Cancer	0.346	0.0195	GAB2,MAPK14,RASGRP1,SOS2,BIRC3,ITGA4,BCL2
IL-9 Signaling	0.342	0.0294	IRS2
G Beta Gamma Signaling	0.334	0.0227	PLCG2,SOS2
IL-17A Signaling in Fibroblasts	0.333	0.0286	MAPK14
Stearate Biosynthesis I (Animals)	0.333	0.0286	ACSL1
Virus Entry via Endocytic Pathways	0.329	0.0225	PLCG2,ITGA4
Interferon Signaling	0.324	0.0278	BCL2
Notch Signaling	0.315	0.027	NUMB
Chronic Myeloid Leukemia Signaling	0.313	0.0217	GAB2,SOS2
Fc_ Receptor-mediated Phagocytosis in Macrophages and Monocytes	0.308	0.0215	GAB2,PTEN
SAPK/JNK Signaling	0.308	0.0215	SOS2,MAP3K2
3-phosphoinositide Biosynthesis	0.308	0.0201	PPP4R1,ALPL,PTEN
Mouse Embryonic Stem Cell Pluripotency	0.303	0.0213	MAPK14,SOS2
Aldosterone Signaling in Epithelial Cells	0.3	0.0199	PLCG2,SOS2,DNAJC3
Gap Junction Signaling	0.3	0.0199	PLCG2,SOS2,MAP3K2
Telomerase Signaling	0.294	0.0208	ETS1,SOS2
Amyotrophic Lateral Sclerosis Signaling	0.289	0.0206	BIRC3,BCL2
Mechanisms of Viral Exit from Host Cells	0.285	0.0244	LMNB1
Role of Hypercytokinemia/hyperchemokinemia in the Pathogenesis of Influenza	0.285	0.0244	IL1B
Germ Cell-Sertoli Cell Junction Signaling	0.281	0.0192	MAPK14,LIMK2,MAP3K2

Melanoma Signaling	0.277	0.0238	PTEN
UVC-Induced MAPK Signaling	0.277	0.0238	MAPK14
Rac Signaling	0.262	0.0194	LIMK2,ITGA4
Cardiac Hypertrophy Signaling	0.261	0.0182	MAPK14,PLCG2,IGF1R,MAP3K2
Pancreatic Adenocarcinoma Signaling	0.25	0.0189	MMP9,BCL2
fMLP Signaling in Neutrophils	0.246	0.0187	FPR2,FPR1
TNFR1 Signaling	0.245	0.0213	BIRC3
Huntington's Disease Signaling	0.245	0.0177	SOS2,IGF1R,HIP1,CREB5
Endothelin-1 Signaling	0.242	0.0179	MAPK14,PLCG2,ECE1
Role of NANOG in Mammalian Embryonic Stem Cell Pluripotency	0.239	0.0183	SOS2,TCL1A
CREB Signaling in Neurons	0.238	0.0178	PLCG2,SOS2,CREB5
Melatonin Degradation I	0.229	0.02	SULT1B1
Amyloid Processing	0.229	0.02	MAPK14
CCR3 Signaling in Eosinophils	0.224	0.0177	MAPK14,LIMK2
Role of Cytokines in Mediating Communication between Immune Cells	0.218	0.0192	IL1B
Semaphorin Signaling in Neurons	0.218	0.0192	LIMK2
UVB-Induced MAPK Signaling	0.213	0.0189	MAPK14
IL-2 Signaling	0.213	0.0189	SOS2
Role of IL-17A in Arthritis	0.208	0.0185	MAPK14
Phospholipases	0.204	0.0182	PLCG2
Superpathway of Melatonin Degradation	0.204	0.0182	SULT1B1
Regulation of Cellular Mechanics by Calpain Protease	0.204	0.0182	ITGA4
Serotonin Degradation	0.199	0.0179	SULT1B1
Glutamate Receptor Signaling	0.199	0.0179	SLC38A1
Synaptic Long Term Potentiation	0	0.0172	PLCG2,CREB5
Axonal Guidance Signaling	0	0.0164	PLXNC1,PLCG2,MMP8,SOS2,LIMK2,MMP9,ITGA4
Synaptic Long Term Depression	0	0.0147	PLCG2,IGF1R

Aryl Hydrocarbon Receptor Signaling	0	0.0148	NQO2,IL1B
Mitochondrial Dysfunction	0	0.00606	BCL2
VDR/RXR Activation	0	0.013	MXD1
Ceramide Signaling	0	0.0125	BCL2
Tight Junction Signaling	0	0.00602	PTEN
TR/RXR Activation	0	0.0118	HP
Regulation of Actin-based Motility by Rho	0	0.0115	ITGA4
RAR Activation	0	0.0107	MAPK14,PTEN
14-3-3-mediated Signaling	0	0.00862	PLCG2
NF-_B Activation by Viruses	0	0.0137	ITGA4
IL-3 Signaling	0	0.0141	GAB2
IL-17 Signaling	0	0.0139	MAPK14
IL-15 Signaling	0	0.0159	MAPK14
Reelin Signaling in Neurons	0	0.0127	ITGA4
Cellular Effects of Sildenafil (Viagra)	0	0.00806	PLCG2
Neuropathic Pain Signaling In Dorsal Horn Neurons	0	0.0101	PLCG2
Relaxin Signaling	0	0.00758	MMP9
Factors Promoting Cardiogenesis in Vertebrates	0	0.0112	MAPK14
Agrin Interactions at Neuromuscular Junction	0	0.0149	ITGA4
Thrombin Signaling	0	0.0107	MAPK14,PLCG2
CDK5 Signaling	0	0.0102	MAPK14
Type II Diabetes Mellitus Signaling	0	0.0175	IRS2,ACSL1
Non-Small Cell Lung Cancer Signaling	0	0.0154	SOS2
Sphingosine-1-phosphate Signaling	0	0.00926	PLCG2
EIF2 Signaling	0	0.0116	SOS2,AGO4
Retinoic acid Mediated Apoptosis Signaling	0	0.0172	PARP15
Hereditary Breast Cancer Signaling	0	0.00794	PTEN

HER-2 Signaling in Breast Cancer	0	0.0132	SOS2
Protein Kinase A Signaling	0	0.0135	PLCG2,PYGL,CREB5,TTN,PTEN
Leptin Signaling in Obesity	0	0.0137	PLCG2
Breast Cancer Regulation by Stathmin1	0	0.0105	SOS2,LIMK2
P2Y Purigenic Receptor Signaling Pathway	0	0.0169	PLCG2,CREB5
Signaling by Rho Family GTPases	0	0.00858	LIMK2,ITGA4
RhoGDI Signaling	0	0.0116	LIMK2,ITGA4
eNOS Signaling	0	0.0148	AQP9,PLCG2
Epithelial Adherens Junction Signaling	0	0.00699	PTEN
G_q Signaling	0	0.0138	RGS2,PLCG2
Regulation of the Epithelial-Mesenchymal Transition Pathway	0	0.0165	ETS1,SOS2,MMP9
Sperm Motility	0	0.00877	PLCG2
Adipogenesis pathway	0	0.00758	TCF7
PCP pathway	0	0.0161	JUNB
Estrogen Receptor Signaling	0	0.00781	SOS2
Cardiac _-adrenergic Signaling	0	0.00758	SLC8A1
Protein Ubiquitination Pathway	0	0.0118	DNAJC3,BIRC3,UBE2D1
JAK/Stat Signaling	0	0.0139	SOS2
Wnt/_-catenin Signaling	0	0.012	FRAT1,KREMEN1
BMP signaling pathway	0	0.0135	MAPK14
GPCR-Mediated Integration of Enteroendocrine Signaling Exemplified by an L Cell	0	0.0143	PLCG2
Gustation Pathway	0	0.00847	P2RY10
phagosome maturation	0	0.00847	HLA-DRB1
Macropinocytosis Signaling	0	0.0147	PLCG2
Calcium Signaling	0	0.0176	ASPH,SLC8A1,CREB5
