# Supplementary figures and images for: Transcriptome-wide study of TNF-inhibitor therapy in rheumatoid arthritis reveals early signature of successful treatment
Source: Arthritis Res Ther. 2021 Mar 10;23:80. doi: 10.1186/s13075-021-02451-9 (PMC7948368; doi:10.1186/s13075-021-02451-9)

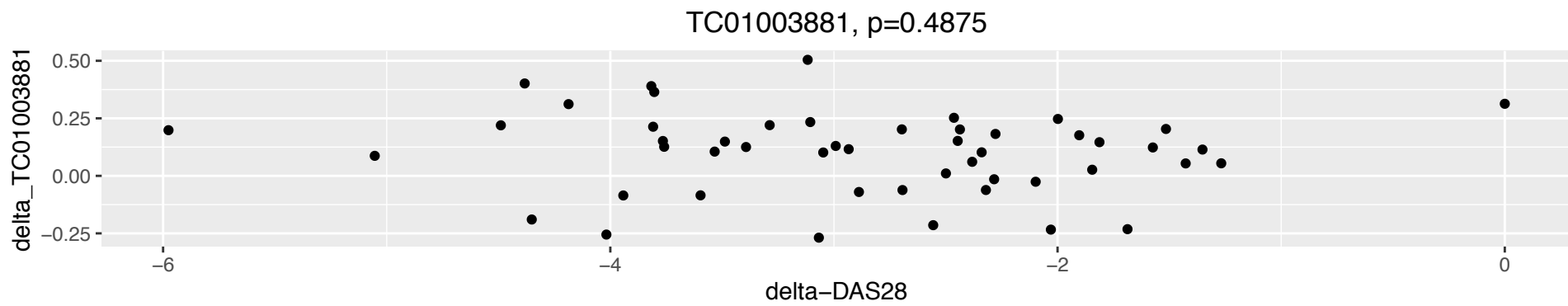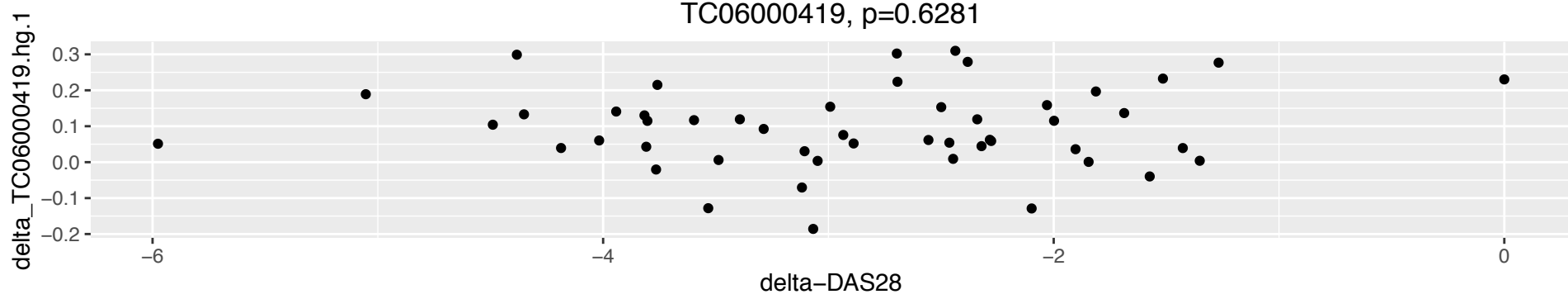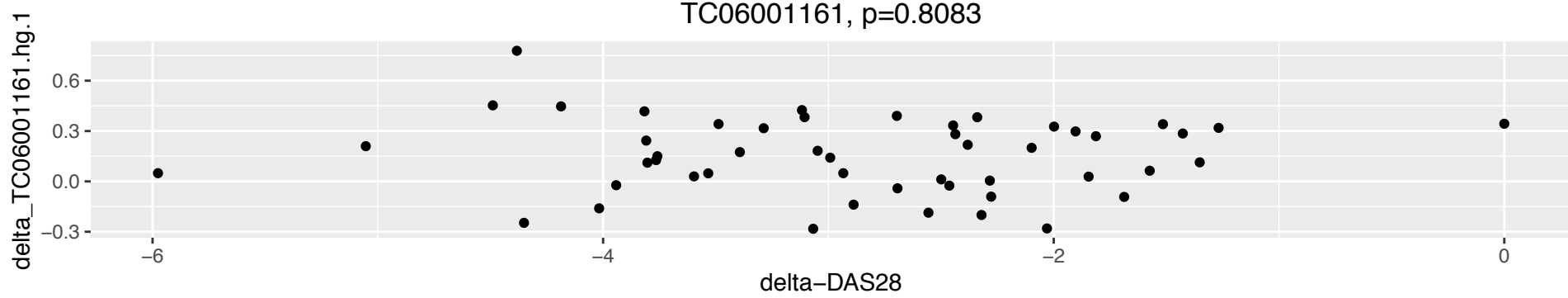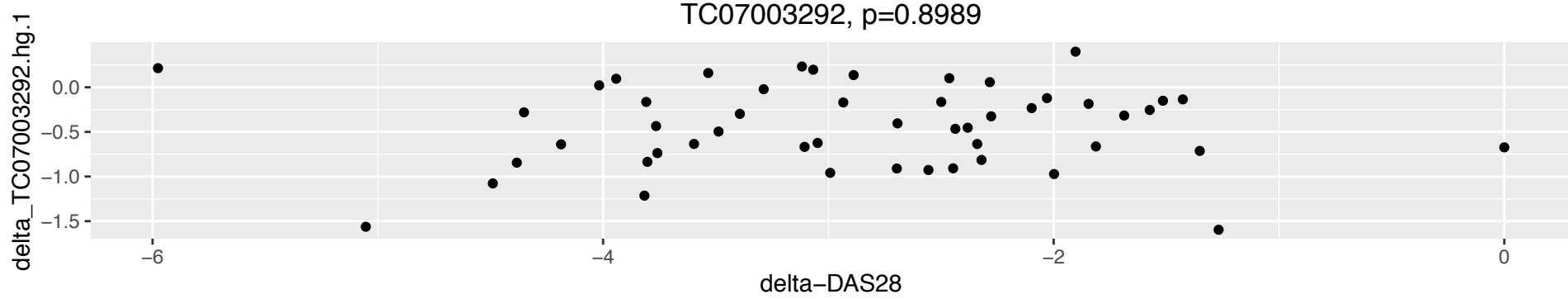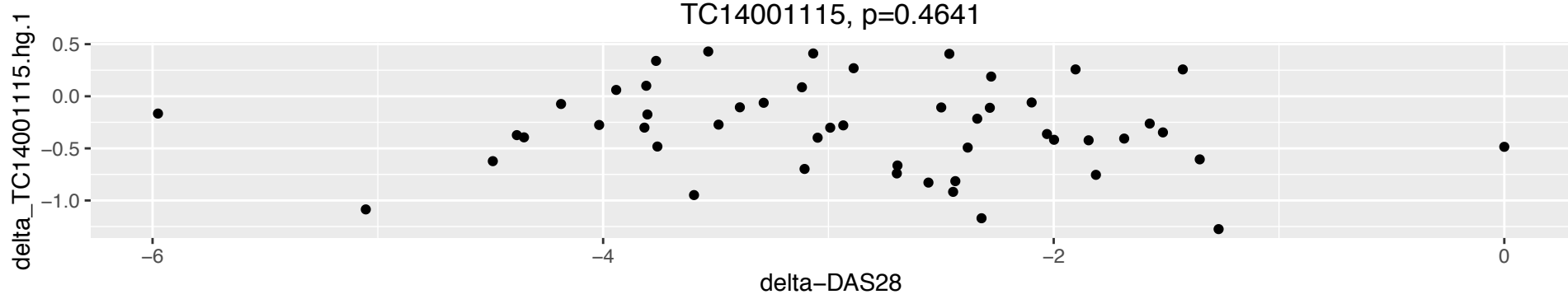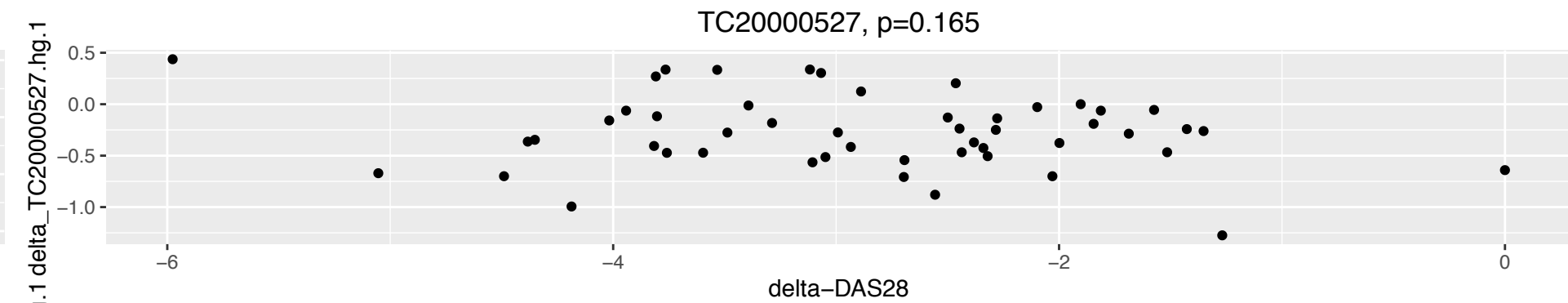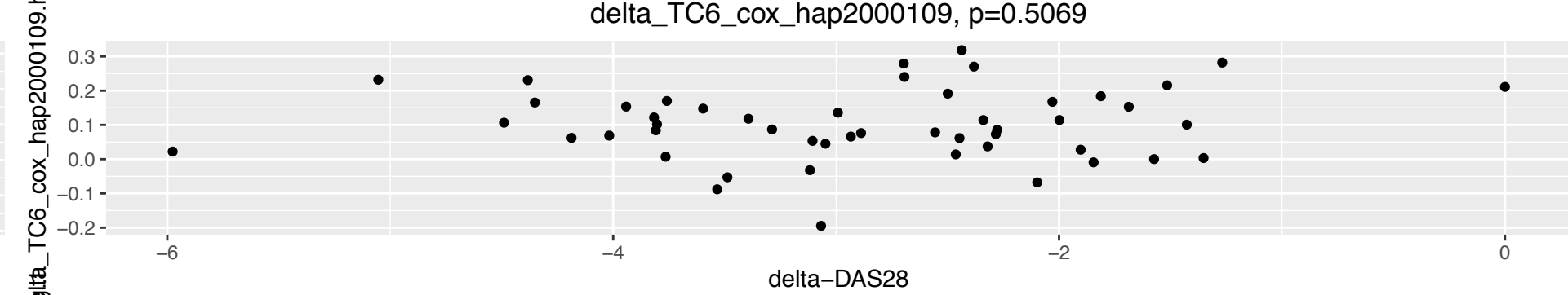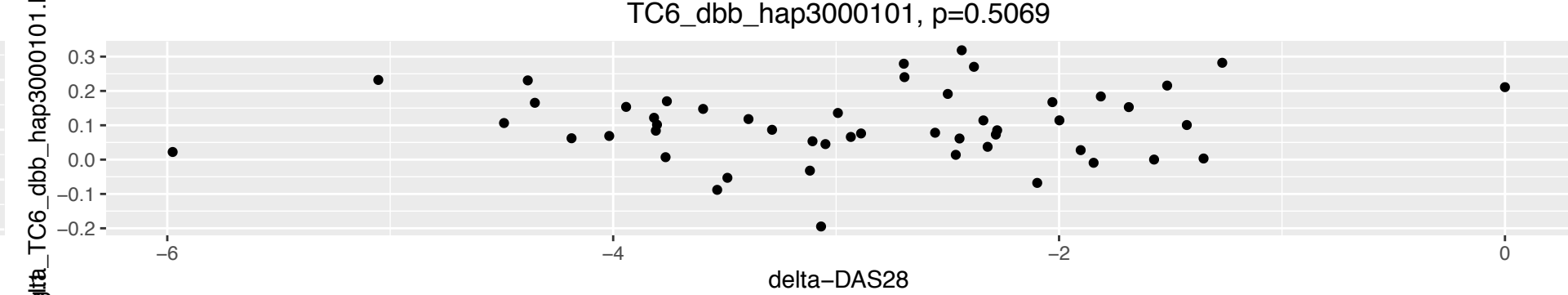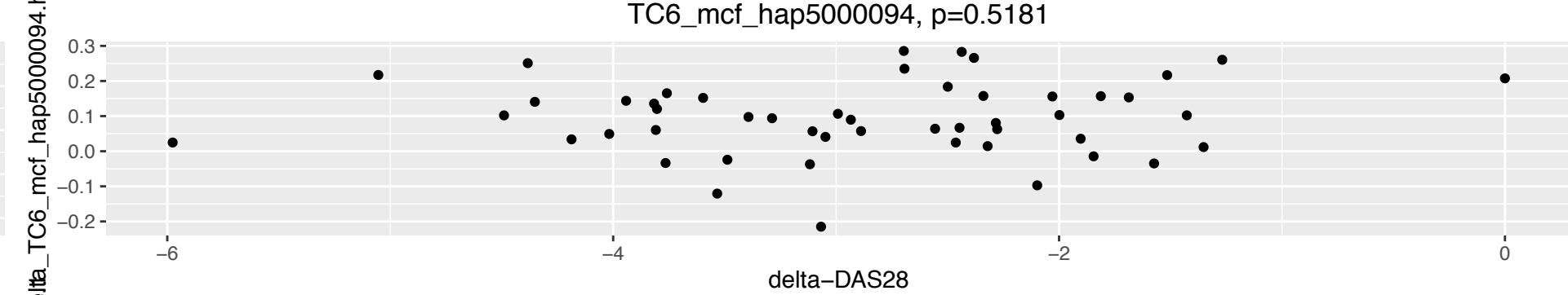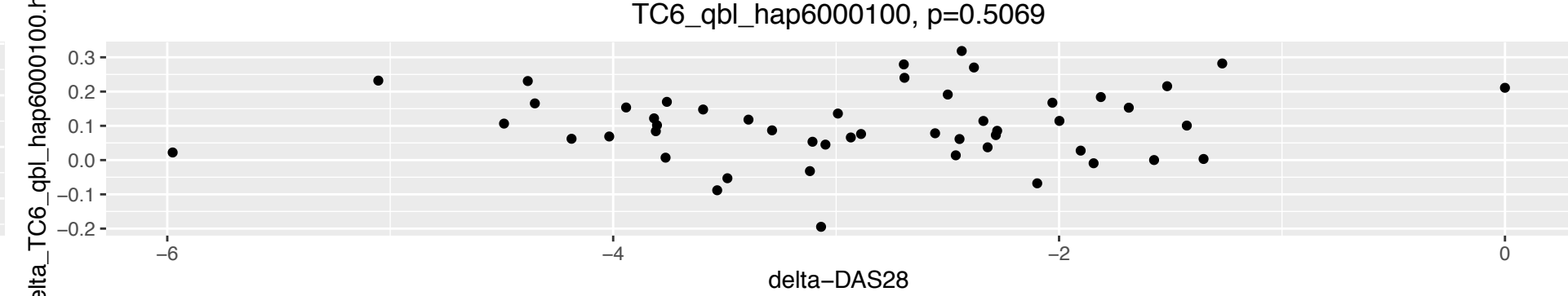

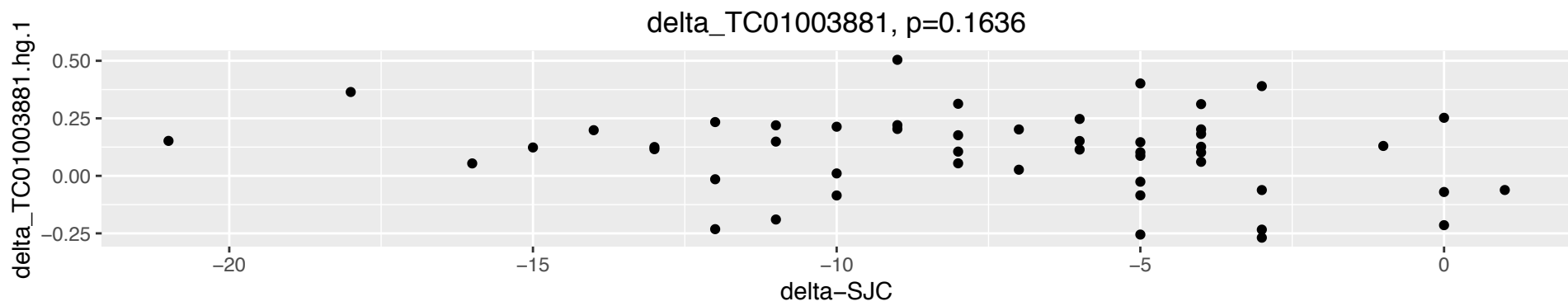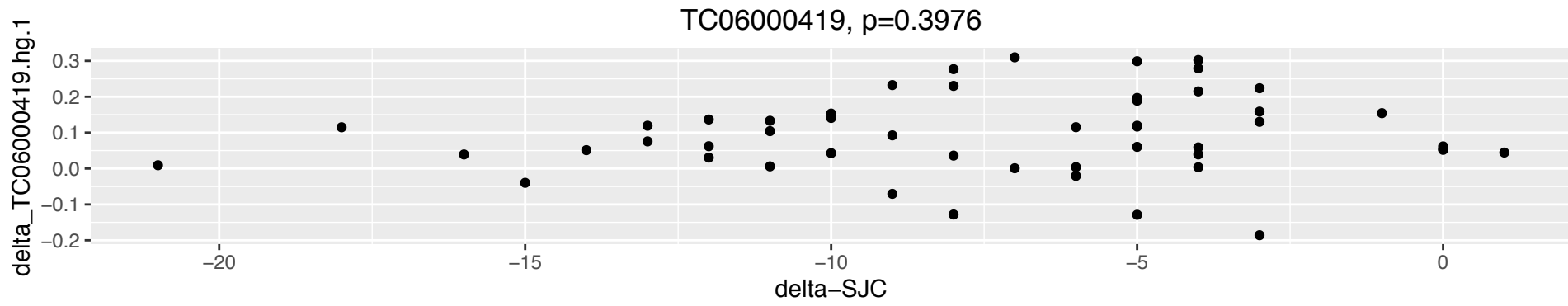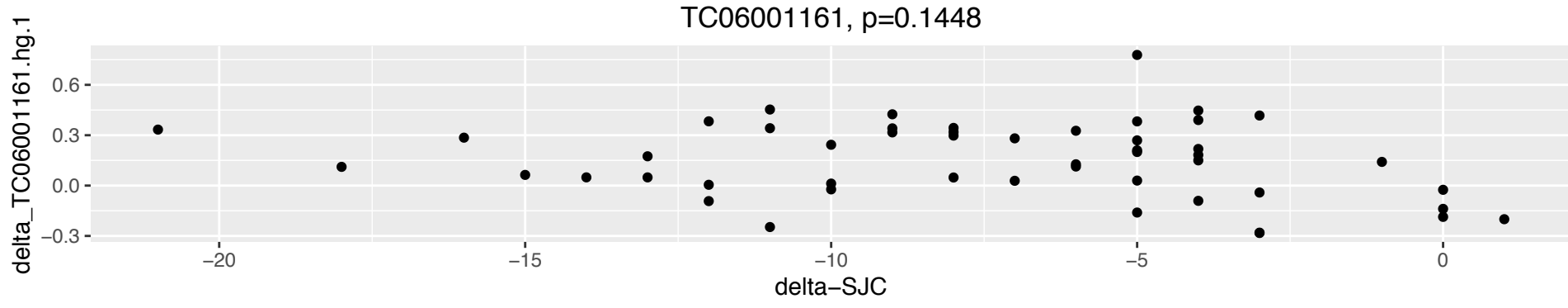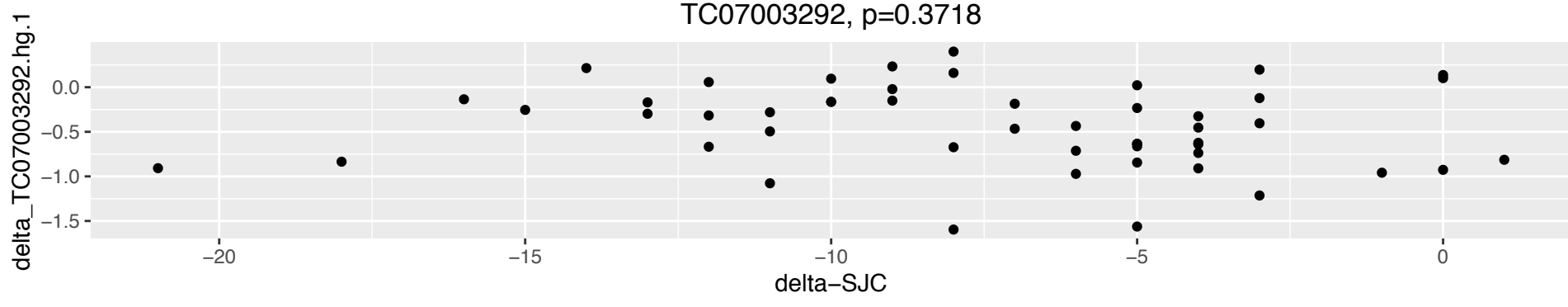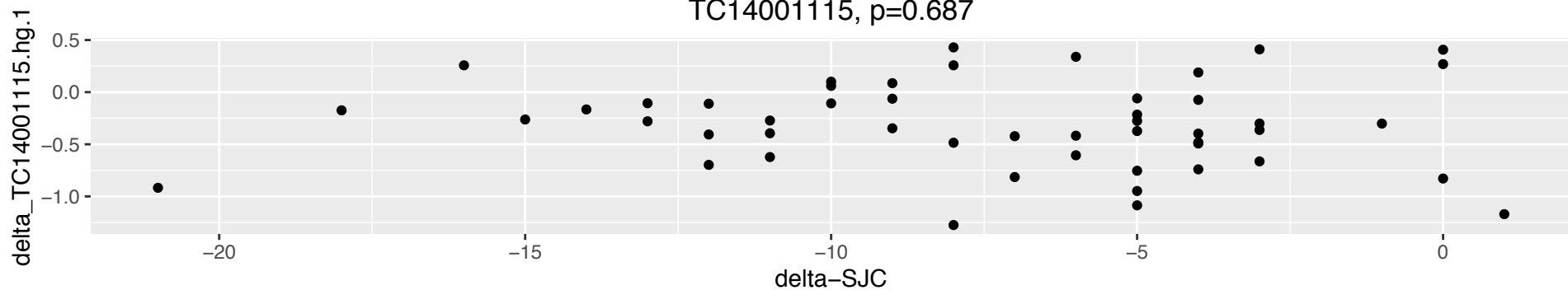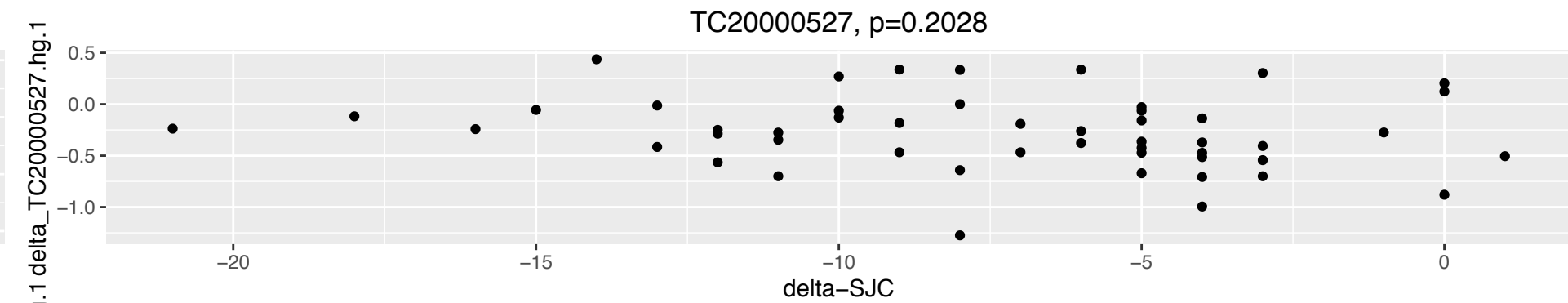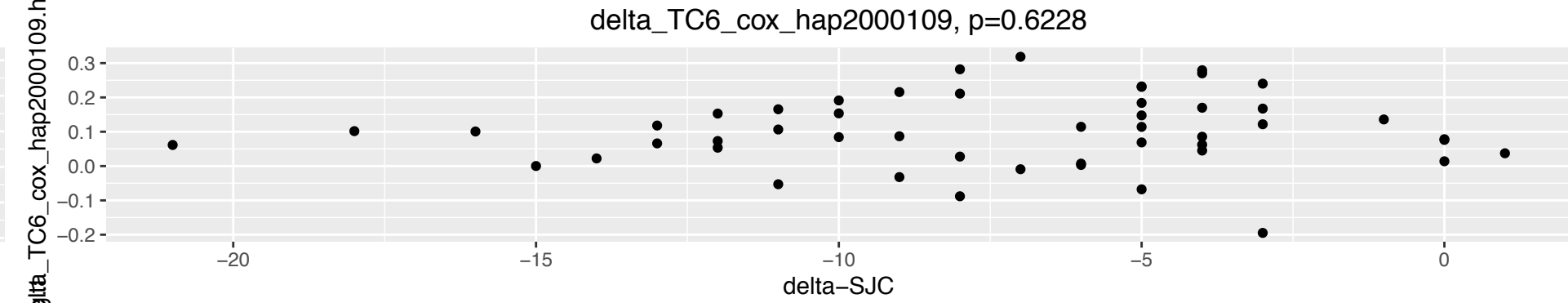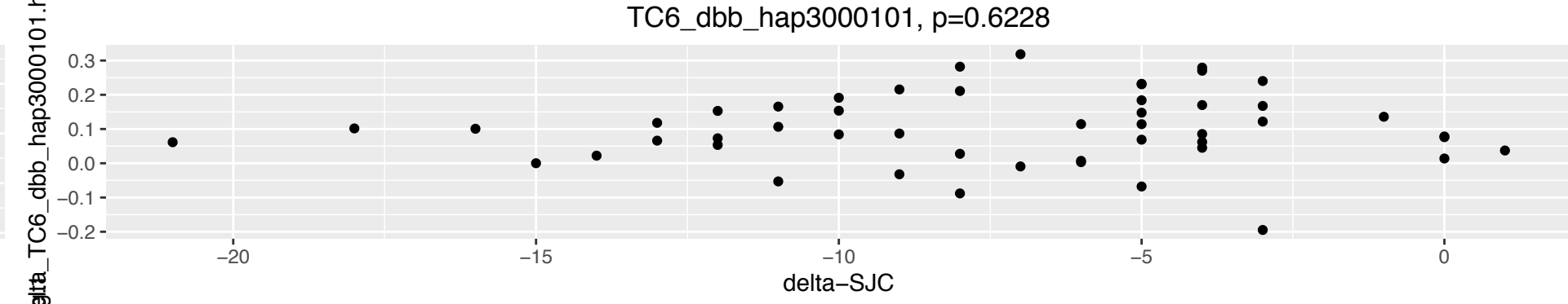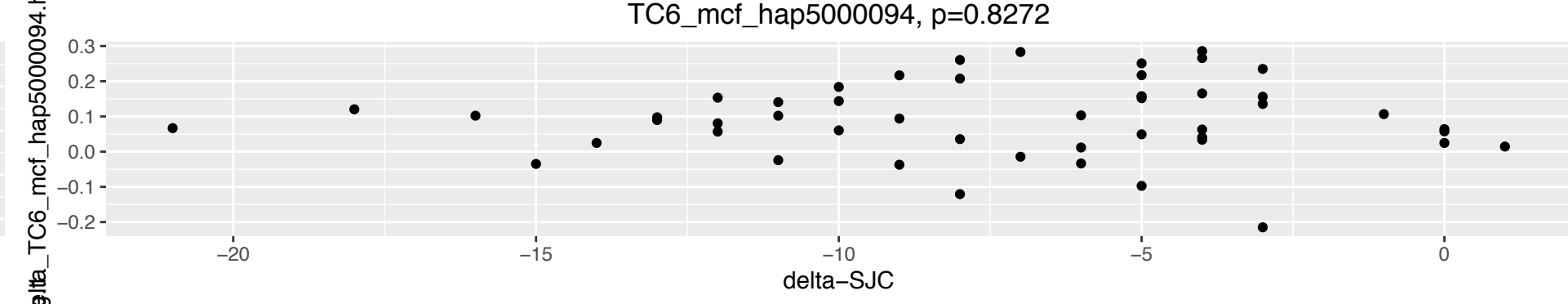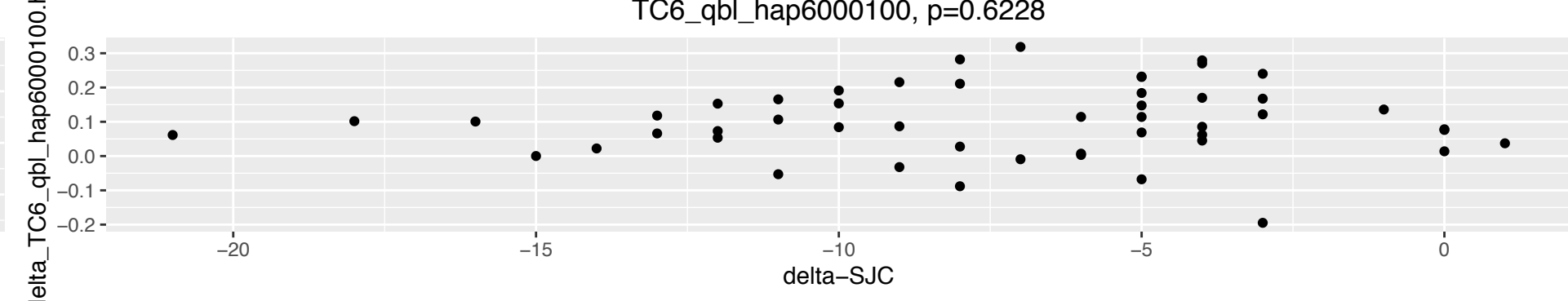

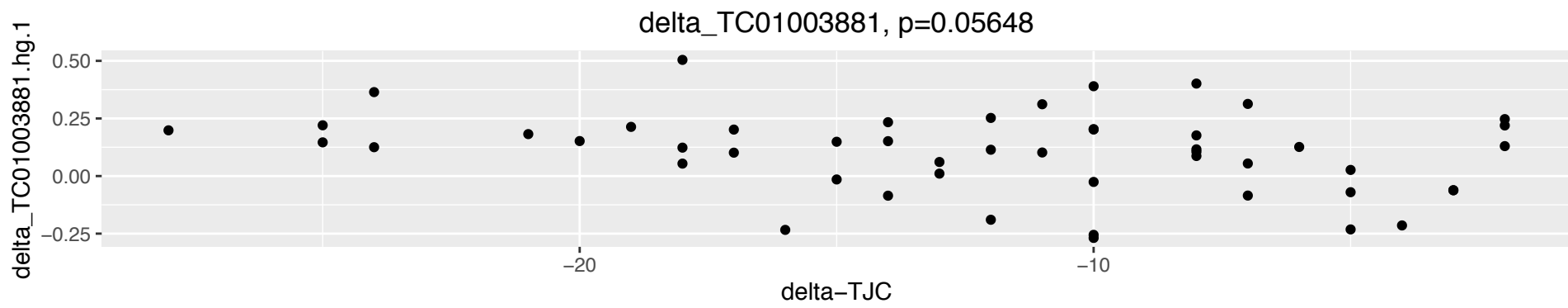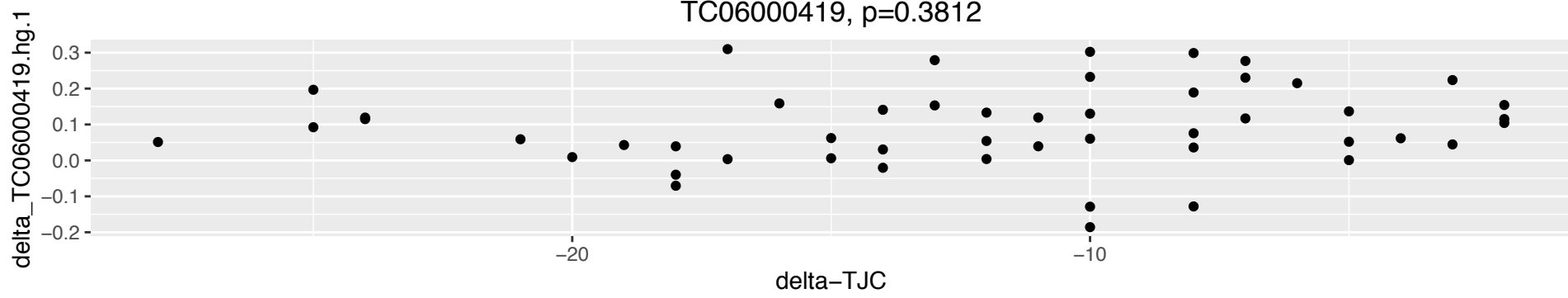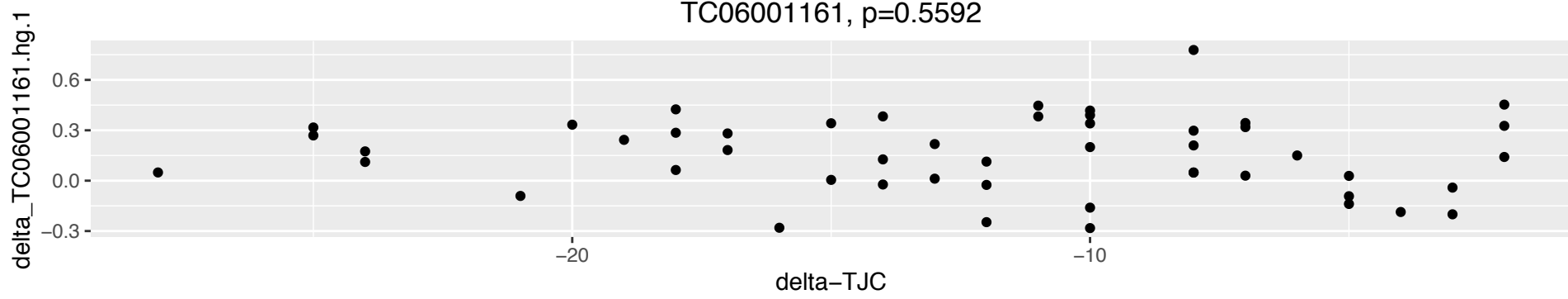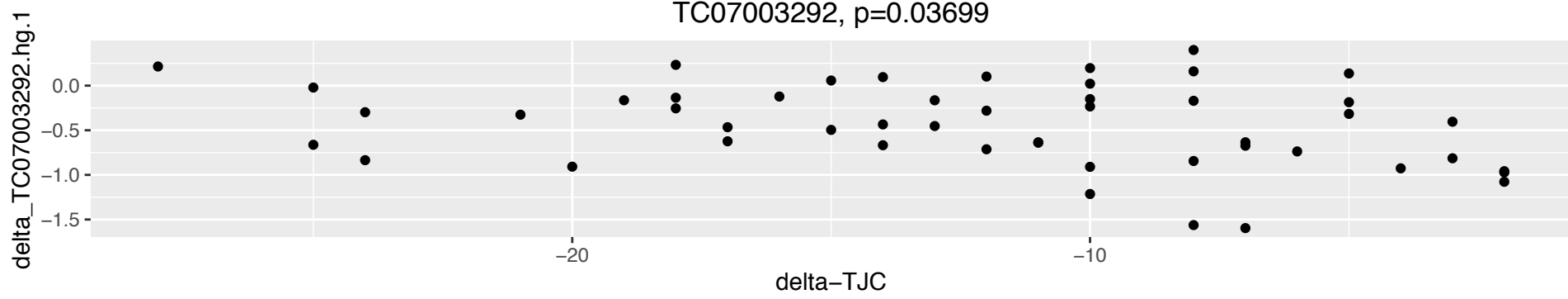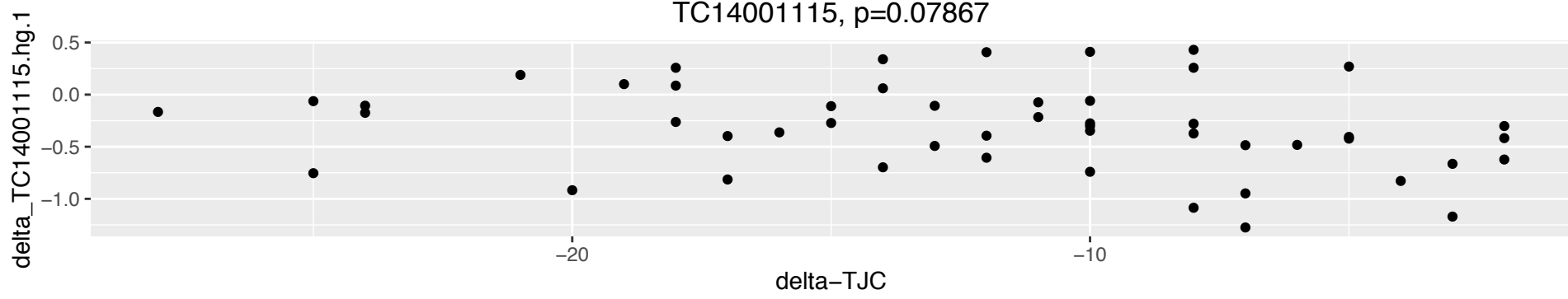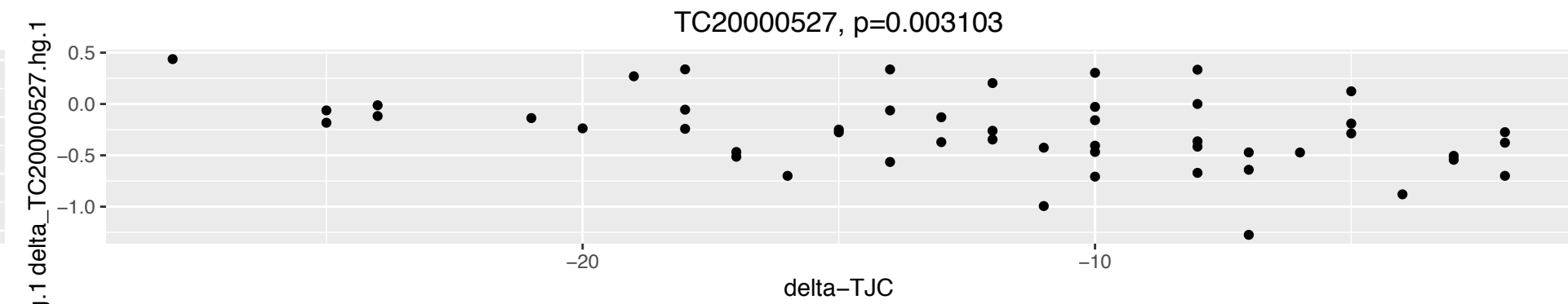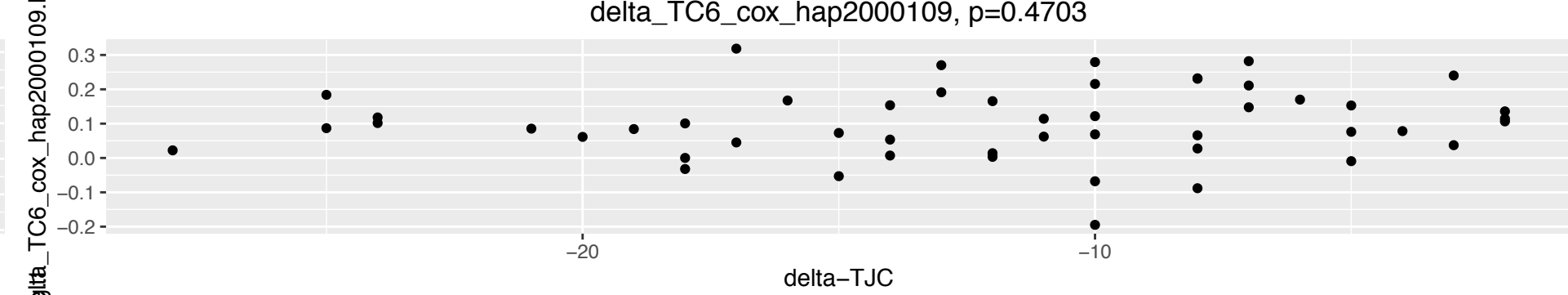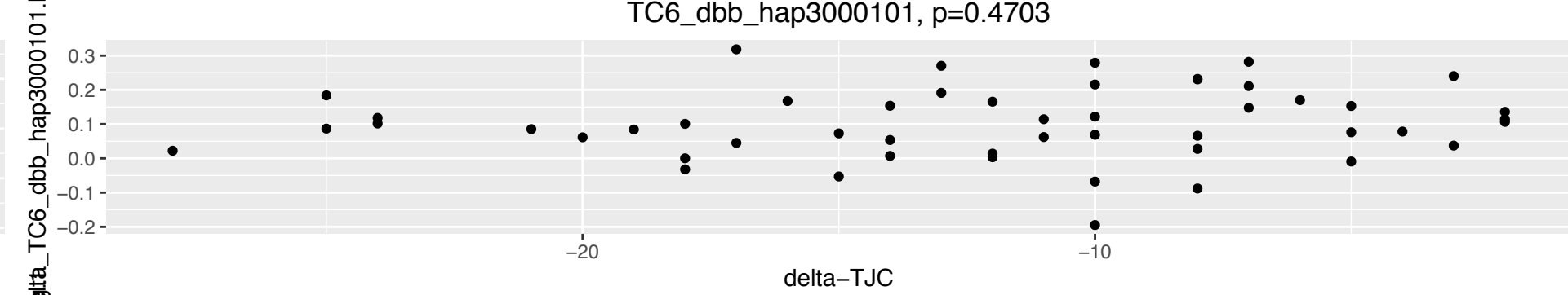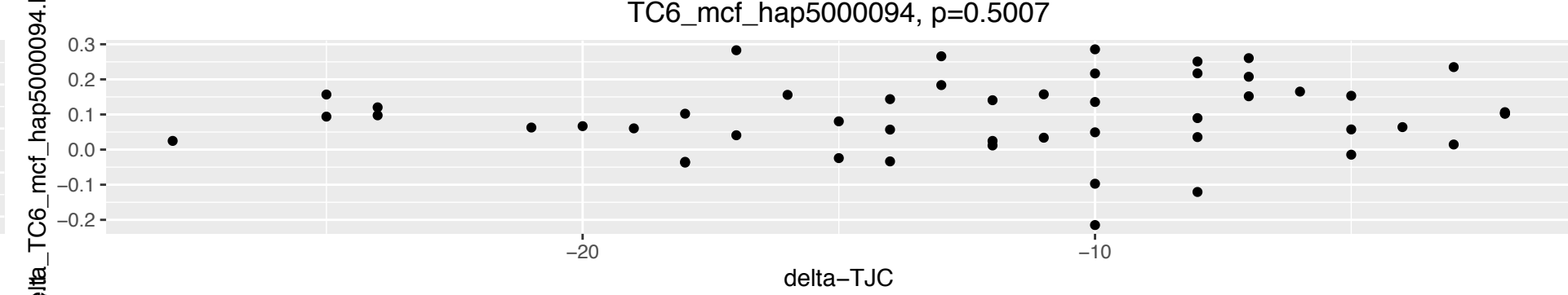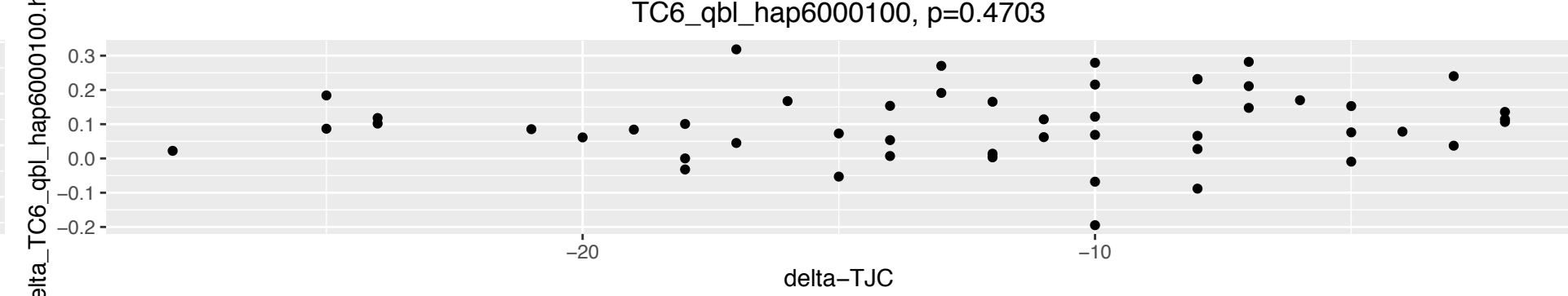

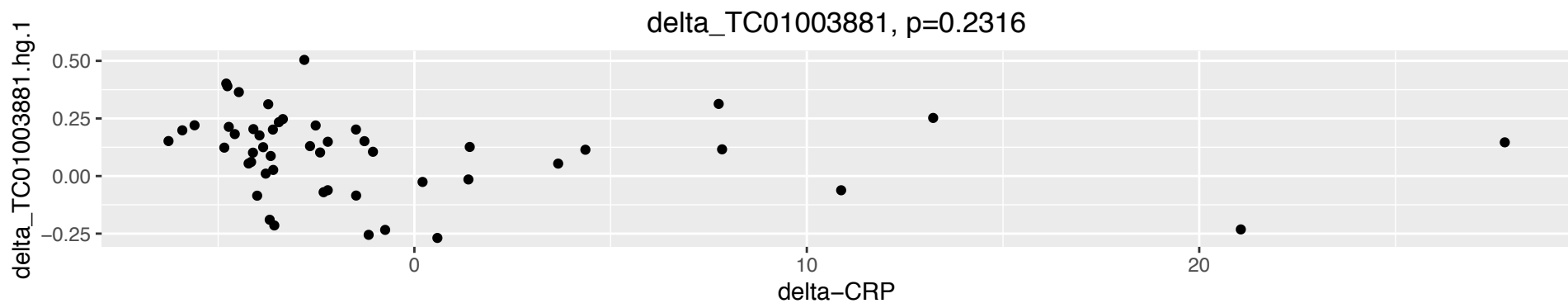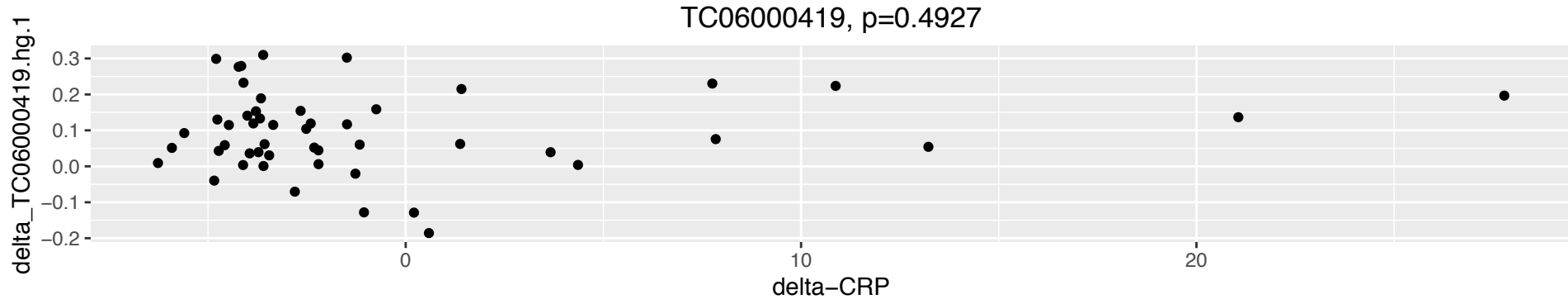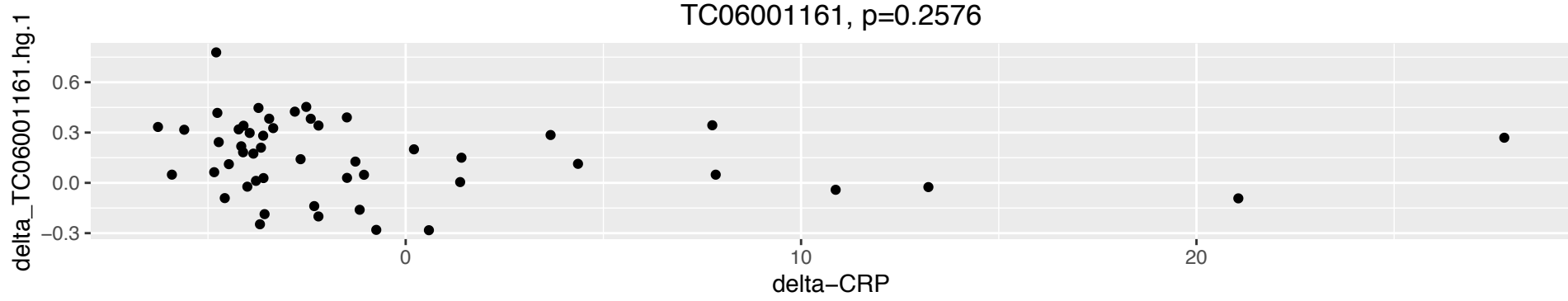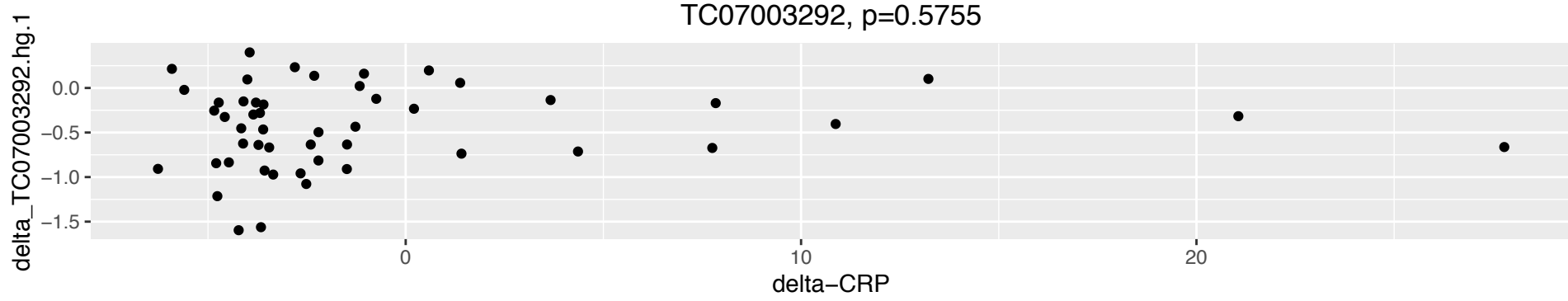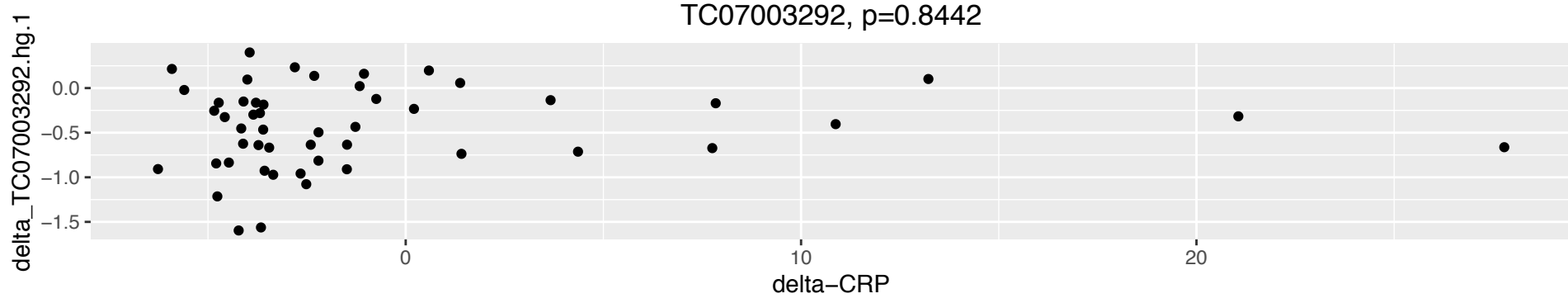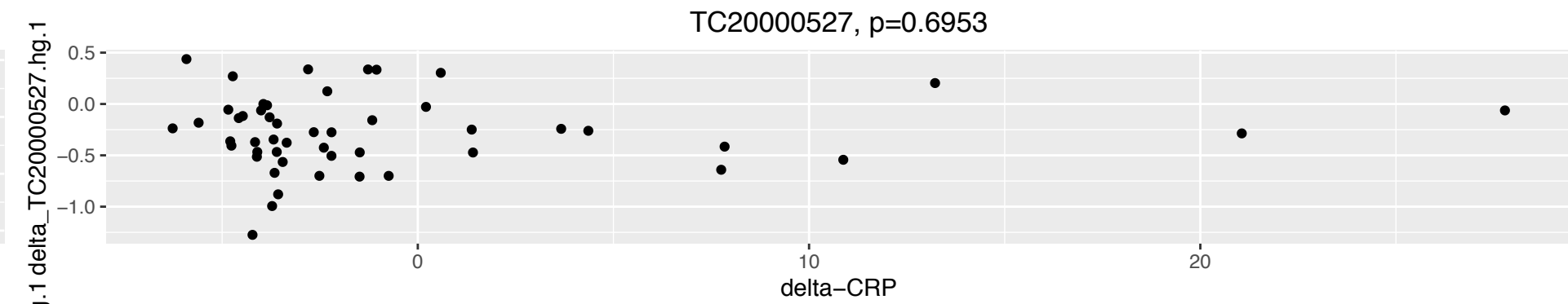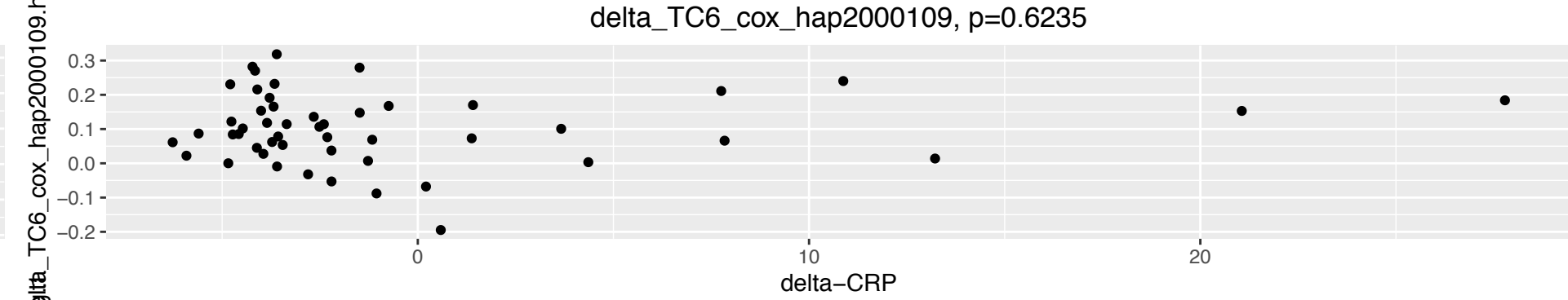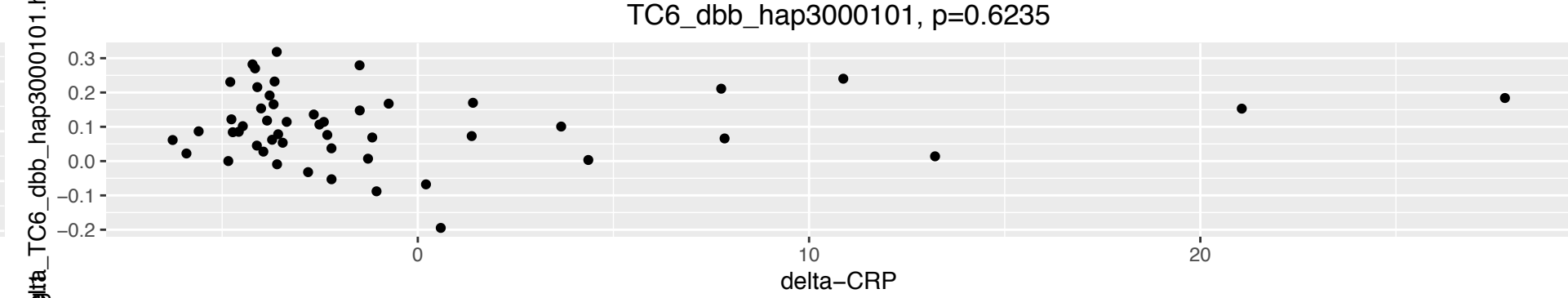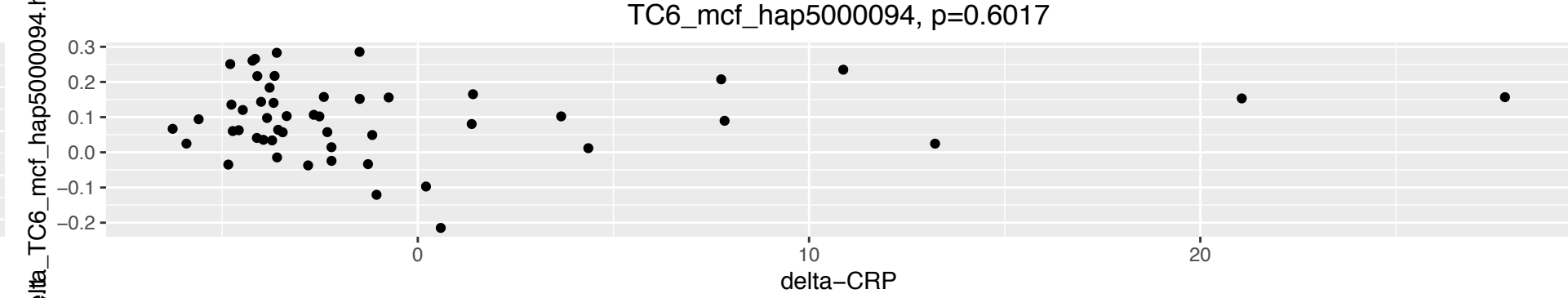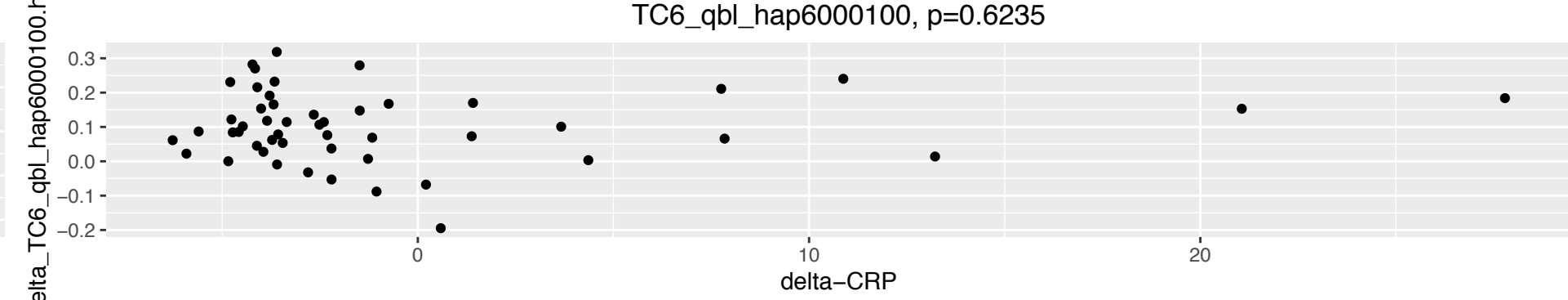

Supplement: Supplementary file 3 — Additional file 3: Figure S1. Correlation between change in transcript expression level and change in DAS28 score in good responders for the top 10 differentially expressed transcripts according to p-value. Figure S2. Correlation between change in transcript expression level and change in swollen joint count (SJC) in good responders for the top 10 differentially expressed transcripts according to p-value. Figure S3. Correlation between change in transcript expression level and change in tender joint count (TJC) in good responders for the top 10 differentially expressed transcripts according to p-value. Figure S4. Correlation between change in transcript expression level and change in C-reactive protein (CRP) levels in good responders for the top 10 differentially expressed transcripts according to p-value. [file 13075_2021_2451_MOESM3_ESM.pdf]
